# Mapping Integrative Decision-Making Frameworks in Advanced Cancer Care: A Scoping Review of Diagnostic, Supportive-Care, and Therapeutic Integration

**DOI:** 10.3390/cancers18193245

**Published:** 2026-10-08

**Authors:** Kazuyuki Murase, Yusuke Sugama, Yohei Arihara, Kohichi Takada, Kunihiko Ishitani

**Affiliations:** 1 Division of Medical Oncology, Department of Internal Medicine, Sapporo Medical University School of Medicine, S-1 W-16, Chuo-ku, Sapporo 060-8543, Hokkaido, Japan; 2 Higashi Sapporo Hospital, 3-3-7-35 Higashi Sapporo, Shiroishi-ku, Sapporo 003-8585, Hokkaido, Japanishitani@hsh.or.jp (K.I.)

**Keywords:** scoping review, advanced cancer, anticancer treatment, palliative care, shared decision-making, prognostic communication, PRISMA-ScR, CFIR

## Abstract

Background/Objectives: Decision-making in advanced cancer care is intertwined with diagnostic profiling, prognostic communication, supportive care, and anticancer treatment selection. An author-developed D-S-T × B/C/E conceptual mapping scaffold—three decision/action domains (Diagnosis, Supportive care, Therapy) and three evaluative dimensions (Biological, Clinical, Existential)—was used to organise the literature, to identify coverage gaps and recurring framework categories, and, as a secondary conceptual synthesis objective, to derive a candidate integrative framework. Methods: The review was conducted according to the Joanna Briggs Institute (JBI) methodology for scoping reviews, pre-registered on OSF, and reported per PRISMA-ScR. PubMed was searched on 21 June 2026, and the final evidence set was derived solely from this search. Eligible records concerning adults with advanced cancer were dual-screened and charted using a structured form. Results: Eighty-two records (2010–2026) were included. Under the strict D-S-T × B/C/E matrix, Supportive care showed the highest joint coverage, particularly S × C (59/82, 72.0%), whereas D × B and D × E were the least represented cells (13/82 each, 15.9%). Eight recurring framework categories were inductively derived from the charted framework descriptors, led by early integrated palliative care (30/82) and prognosis-informed shared decision-making (27/82). Twenty-two records met an emergence-candidate screen defined at the synthesis stage, of which three received the maximum cross-axis coupling score of 6/6 from both reviewers. Conclusions: The advanced-cancer decision-framework literature clusters around a small number of architectures with uneven D-S-T × B/C/E coverage. Diagnosis-centred frameworks coupled with biological or existential dimensions were relatively under-represented within our mapping and represent priority targets for future primary research; the proposed Adaptive framework provides one structure in which this research could be conducted.

## Simple Summary

Patients with advanced cancer face closely linked decisions about diagnostic testing, supportive care, and anticancer treatment. Many decision-making frameworks have been proposed to help clinicians and patients navigate these choices, but it has been difficult to see the overall pattern across the field. In this scoping review we mapped 82 published records against a simple grid that considers three decision areas—Diagnosis, Supportive care, and Therapy—alongside three evaluative dimensions—biological, clinical, and existential. We identified eight recurring framework categories, of which early integrated palliative care and prognosis-informed shared decision-making were the most common. Within our mapping framework, two relatively under-represented combinations were identified: frameworks that link diagnostic or prognostic tools to biological dimensions, and to existential dimensions such as dignity and meaning. Our map is intended to help clinicians, researchers, and policy makers design more coordinated approaches to advanced-cancer care.

## 1. Introduction

Decision-making about anticancer treatment in adults with advanced or metastatic cancer has grown increasingly complex over the past two decades. Curative intent is often not feasible, prognostic uncertainty is substantial, and the therapeutic menu—cytotoxics, molecularly targeted agents, immunotherapies, antibody-drug conjugates, CAR-T cells, and bispecific engagers—continues to outpace the decision-support evidence base [1]. In parallel, the same patients face diagnostic decisions (biomarker profiling, prognostic scoring, prognostic disclosure) and supportive-care decisions (palliative-care integration, symptom management [2], advance care planning). Previous systematic reviews have addressed information provision and participation in treatment decisions in advanced cancer [3], the effects of geriatric assessment on oncologic decisions and non-oncologic interventions in older adults with cancer [4], and experiences of shared decision-making in palliative cancer care [5]. Building on these complementary syntheses, the present scoping review maps how diagnostic, supportive-care, and therapeutic decision structures are represented across the literature.

Integrative frameworks for serious illness have a long lineage: Engel’s biopsychosocial model [6], Saunders’ Total Pain concept [7], Wagner’s Chronic Care Model [8], the WHO palliative-care framework [9], and Bruera and Hui’s cancer-trajectory conceptualisation [10] articulated the multidimensional and longitudinal nature of care for people with serious illness. The D-S-T × B/C/E scaffold used in the present review does not replace these older frameworks. It is a domain-specific mapping scaffold for the more recent, decision-focused literature on advanced cancer care, in which the diagnostic, supportive, and therapeutic axes are increasingly discussed as coupled rather than sequential.

Within that recent literature, several architectures unify the three decision families. Early integrated palliative care, geriatric-assessment-driven oncology [11], shared decision-making decision aids informed by prognostic disclosure, and trigger- or criterion-based palliative-care integration [12] are among the more widely reported architectures. Whether these frameworks are structurally similar or address distinct decision problems has not, to our knowledge, been mapped in a formal scoping review.

The two facets serve different conceptual roles. The D-S-T axes define decision/action domains: D captures diagnostic and prognostic information generation, interpretation, and disclosure; S captures supportive-care assessment and intervention; and T captures anticancer-treatment selection, modification, continuation, or cessation. In contrast, B/C/E should not be interpreted as a uniform set of outcomes; they are broader evaluative dimensions that capture the types of signals or constructs brought into, generated by, or used to assess decisions. B includes biological disease-state variables and outcomes (e.g., molecular status, response, progression, and survival); C includes clinical state and clinical or patient-reported outcomes (e.g., toxicity, symptoms, function, quality of life, and healthcare use); and E includes patient-defined existential and preference-related constructs (e.g., meaning, dignity, hope, values, priorities, acceptable burden, information preferences, and prognostic awareness), which may function as decision inputs, process variables, or existential outcomes depending on the study. Advance care planning (ACP) and goals-of-care (GOC) documentation and goal concordance are treated as process indicators rather than proxies for existential well-being. Implementation constructs such as feasibility, acceptability, adoption, fidelity, and reach are considered separately from B/C/E; CFIR is used to organise implementation determinants. Accordingly, a D-S-T × B/C/E matrix–cell denotes co-representation or coupling between a decision/action domain and a B/C/E dimension, not necessarily a causal decision-to-outcome relationship. The 3 × 3 matrix is an author-developed conceptual mapping scaffold only; it is not a validated instrument and is not used for quality grading. Consistent with scoping-review methodology [13,14,15], the primary objectives of this review were descriptive: (i) to map the extent, range, and nature of the literature; (ii) to identify recurring framework categories by inductive consolidation of charted framework descriptors; (iii) to map facilitators and barriers to the CFIR 2.0 domains [16]; and (iv) to identify coverage gaps within the D-S-T × B/C/E matrix. As a secondary conceptual synthesis objective, we additionally sought to integrate the mapped findings into a candidate framework for prospective development and testing.

## 2. Materials and Methods

### 2.1. Protocol and Registration

The protocol followed the JBI methodology for scoping reviews [13]; this approach was chosen to map the extent, range, and nature of the literature rather than answer a comparative-effectiveness question [17]. The protocol was prospectively registered on OSF (Version 1.0; 21 June 2026; DOI 10.17605/OSF.IO/ZKD53). Amendments dated 20, 21, and 22 September 2026, and a registration clarification dated 22 September 2026, are summarised in Supplementary File S1. Exploratory native database searches undertaken during revision were not incorporated into final eligibility assessment or synthesis; the final evidence set derives from the June PubMed search. The AI-assisted pre-filter and Stage 0 audit were specified in the original protocol, which defined the audit pool as the Stage B AI-EXCLUDE set; the audit was applied to the combined Stage A and Stage B exclusions, which is wider than registered. During revision, full-text eligibility assessment of the original 106 records was strengthened from 20% independent verification to 100% independent dual assessment. Inductive typology development was pre-specified, whereas AI assistance in drafting the charted entries and in consolidating the charted labels was not; both are reported as assistance steps with author verification. Reporting follows PRISMA-ScR [18]; the 22-item checklist is provided in Supplementary File S2.

### 2.2. Eligibility Criteria (PCC)

The primary research question was: what integrative frameworks, models, interventions, pathways, or decision-support tools that explicitly address at least one of diagnosis (D), supportive care (S), or therapy (T) within an integrative advanced-cancer decision-making context are reported in the adult advanced-cancer care literature, and what are their characteristics, facilitators, and barriers to implementation? Eligibility was specified using the Population–Concept–Context (PCC) framework.

Population: adults (≥18 years) with advanced or metastatic cancer per each report’s definition (stage III unresectable/stage IV, recurrent metastatic disease, or explicit advanced/incurable disease). Paediatric-only records and curative-intent-only records without an advanced-cancer subgroup were excluded. Borderline mixed-stage cohorts were resolved by K.M. + Y.S. consensus, requiring ≥50% advanced-stage patients or explicit advanced-stage subgroup reporting.

Concept: decision-making in advanced cancer care within an integrative framing, operationally defined as substantive attention to at least one D-S-T axis as an action-linked framework element situated within an integrative advanced-cancer decision-making context. Records describing single-modality measurement instruments only, and records addressing a single symptom or an isolated intervention without integrative decision-making framing, were excluded.

Context: decision-making in advanced cancer care across outpatient, inpatient, home, or hospice settings, including situations in which systemic anticancer therapy is considered, delivered, modified, or discontinued; no geographic restriction. English- or Japanese-language original reports, protocols, guidelines, or framework/model papers were eligible. Case reports, editorials, comments, letters without primary content, bibliometric analyses, and animal-only reports were excluded. The full E1–E13 exclusion coding scheme is provided in the pre-registered OSF protocol.

Operational definitions. The publication record was the unit of inclusion and counting; we mapped the decision structure described within it, regardless of study design. A mappable structure linked assessment, interpretation, or deliberation to a choice, action, or reassessment in an integrative advanced-cancer care context. Depending on the record, this could be a conceptual framework or model, a decision aid, an assessment-to-action pathway in a trial or intervention, a planned pathway in a protocol, a decision process described in an observational study, or the decision logic of a guideline. Definitions of the six framework-related terms are provided in Supplementary File S3; integration depth is defined separately in Section 2.7 and Supplementary File S3.

### 2.3. Information Sources

PubMed was the only native bibliographic database search contributing to the final evidence set. The search executed on 21 June 2026 was complemented by PubMed-based proxy scans for Cochrane CDSR and CINAHL, a ClinicalTrials.gov scan, and a relevance-ranked sample of 200 OpenAlex records. The PubMed-based proxy scans could not identify records outside PubMed, and the complementary scans contributed no additional included records. The executed search procedures and deviations from the registered information-source plan are documented in Supplementary File S1.

### 2.4. Search

The PubMed search executed on 21 June 2026 combined four concept blocks—Population, Treatment, Intervention, and Methodological framing—using AND, with predefined publication-date, language, and publication-type filters applied at execution. Within each block, controlled vocabulary and free-text synonyms were combined with OR. The complete executed query, the field tags and filters, the rationale for the four-block structure, the verbatim proxy queries, the retrieval count (*n* = 2648), and the PRESS 2015 checklist documentation are provided in Supplementary File S1.

### 2.5. Selection of Sources of Evidence

Screening was conducted in Rayyan, a web application for which no user-facing version number is issued (Rayyan Systems, Inc., Cambridge, MA, USA; https://www.rayyan.ai/, accessed on 31 July 2026). Pre-registered rule-based exclusions were first applied (Stage A), followed by an AI-assisted pre-filter (Stage B; Supplementary File S4). Records excluded by these procedures were subject to a pre-registered blinded dual-reviewer audit by K.M. and Y.S. using a random sample of 151 records drawn from the combined Stage A and Stage B exclusion pool; the registration had specified the Stage B AI-EXCLUDE set alone, so the audited pool is wider than registered. The audit evaluated the false-exclusion rate against the pre-specified 5% acceptance threshold; one record judged eligible after reviewer reconciliation was returned to screening. Full audit procedures, cross-tables, agreement statistics, and confidence intervals are provided in Supplementary File S5.

**Figure 1:**
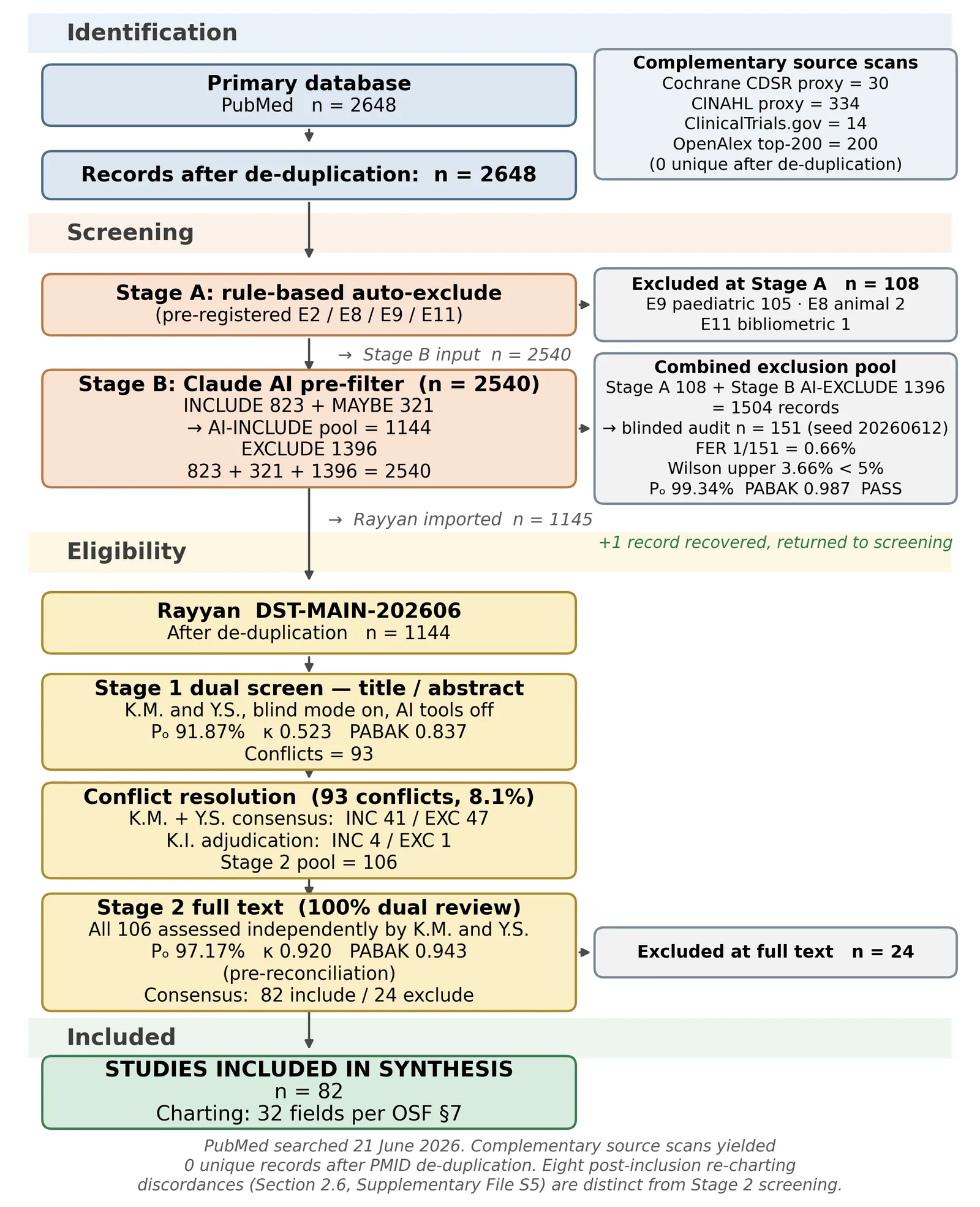
PRISMA-ScR flow diagram of study selection. Of 2648 records identified from PubMed, 82 were included in the final synthesis after staged screening and 100% independent dual full-text assessment. Complementary source scans contributed no additional records to the final evidence set. Inter-rater statistics are shown for the dual-reviewer stages. AI, artificial intelligence; CDSR, Cochrane Database of Systematic Reviews; CINAHL, Cumulative Index to Nursing and Allied Health Literature; EXC, exclude; FER, false-exclusion rate; INC, include; K.I., senior reviewer; K.M., first reviewer; OSF, Open Science Framework; PABAK, prevalence- and bias-adjusted kappa; PMID, PubMed identifier; PRISMA-ScR, Preferred Reporting Items for Systematic Reviews and Meta-Analyses extension for Scoping Reviews; P_o_, observed agreement; Y.S., second reviewer; κ, Cohen’s kappa.

After audit recovery and de-duplication, 1144 records underwent independent blinded title/abstract screening by K.M. and Y.S., with disagreements resolved by consensus and K.I. available for adjudication. In response to peer-review recommendations, the pre-registered full-text procedure was strengthened from assessment by K.M. with 20% independent verification to 100% independent dual full-text assessment. All 106 Stage 2 records were independently assessed by K.M. and Y.S. using the predefined eligibility criteria and E1–E13 exclusion codes before reconciliation. Consensus yielded 82 included and 24 excluded records. The complete screening flow is shown in Figure 1, and inter-rater reliability across screening stages is reported in Supplementary File S5.

### 2.6. Data Charting Process

A 32-field structured charting form (Supplementary File S3) was developed a priori and pilot-tested on the first five records; no substantive field revisions were required. Charting was AI-assisted: an initial draft entry was generated for each record from its full text, and K.M. verified and corrected every field of all 82 included records against the source article, including the implementation (CFIR) narrative block. Only the author-finalised values were used in the analyses. For the two records added during final eligibility reconciliation, framework categories were assigned directly using the finalised category definitions (Section 3.3), rather than through the free-text label-consolidation process applied to the original 80 records. Field-level completion for both groups is tabulated in Supplementary File S3.

A pre-specified 20% verification sample (*n* = 16) was randomly selected from the original 80-record included set. Y.S. independently re-charted these records on the same form without access to K.M.’s corresponding classifications; reviewer values were compared only after the independent re-charting was complete. Agreement was assessed using Cohen’s kappa [19] and PABAK [20], the latter reported because of prevalence imbalance in some blocks [21,22], and eight discordant classifications were adjudicated by K.I. Full sampling procedures, field-level agreement statistics, and adjudication results are provided in Supplementary File S5.

### 2.7. Data Items

Charted fields comprised (A) bibliographic metadata; (B) study design and population descriptors; (C) setting descriptors; (D) narrative descriptions of the D, S, and T axes; (E) narrative descriptions of facilitators and barriers coded to CFIR domains; (F) B/C/E dimension flags for B, C, and E; (G) authors’ conclusions and limitations; (H) inductive typology labels (up to two per record); (I) integration depth and a supplementary all-3-dimensions flag; (J) tracking fields; and (K) the fields in which Y.S.’s independent re-charting of the 20% verification sample was recorded (Section 2.6). Full definitions for all eleven blocks are provided in Supplementary File S3. Integration depth was charted as a separate variable reflecting the number of D-S-T domains functionally integrated within the framework or care pathway, rather than the number of non-Absent domains. Central domains contributed to depth; Mentioned domains contributed only where explicitly linked functionally to another domain. The operational rules for this judgement are given under field 25 of Supplementary File S3.

### 2.8. Synthesis of Results

Descriptive counts and cross-tabulations were computed using a reproducible Python pipeline (Python 3.12.10, with NumPy 2.4.6, pandas 2.3.3, SciPy 1.17.1, scikit-learn 1.9.0, statsmodels 0.14.6, openpyxl 3.1.5, and Matplotlib 3.10.9). For the D-S-T × B/C/E matrix, each D-S-T domain was classified per record as Central, Mentioned, or Absent. Central indicated an explicit organising, decision-relevant, or action-triggering role; Mentioned indicated explicit presence without a central decision or action role; and Absent indicated no meaningful representation. Strict analyses counted Central only, whereas inclusive analyses counted Central or Mentioned. K.M. classified all 82 included records; Y.S. independently applied the same rules to the 80 records independently included by both reviewers, while the two reconciliation-added records received consensus codes and are not part of the reliability analysis. Full operational rules and the predefined negative examples are provided in Supplementary File S3, and the reliability statistics in Supplementary File S5.

Framework categories were developed inductively from the free-text framework descriptors recorded by the charting reviewer; the category names are therefore generic descriptive labels rather than terms coined by the source authors. AI assistance was used to consolidate the heterogeneous descriptors into recurring categories, with all final assignments reviewed and finalised by the authors. Up to two categories could be assigned per record, so category frequencies are non-exclusive and do not sum to 100%. Category definitions and the complete mapping are provided in Supplementary Table S1 and Supplementary File S6.

For the cross-axis coupling analysis, the 22 records meeting the emergence-candidate screen were evaluated using a structured three-component rubric developed during revision. Explicit D-S-T linkage, triggering or reassessment, and longitudinality were each scored from 0 to 2 against predefined operational criteria, yielding a total score of 0–6 per record. K.M. and Y.S. scored all 22 candidate records using the same rubric. Agreement was assessed using linear-weighted Cohen’s κ for each ordinal component and Spearman’s rank correlation for total scores. The component scores, total scores, and supporting evidence are provided in Supplementary Table S2.

CFIR mapping was performed in two steps. A predefined keyword dictionary was used only to identify candidate implementation-related text within the charted facilitator and barrier narratives, and keyword matches were not treated as final classifications; K.M. then reviewed each candidate in context and assigned a CFIR 2.0 domain [16] only where the contextual meaning supported it. To assess reproducibility, Y.S. independently classified facilitator and barrier presence or absence across the five CFIR domains in the pre-existing random 16-record charting-verification sample, giving 160 binary record–domain–role classifications; observed agreement, Cohen’s κ and PABAK were computed before reconciliation, and the discordant classifications were adjudicated by K.I. The mapping procedure and the per-record assignments, with matched terms or adjudication provenance and a charted evidence excerpt for each, are provided in Supplementary Files S3 and S7.

The unit of analysis was the publication record. To address heterogeneity in the included evidence, records were classified separately by study design/evidence type and by primary mapped architecture, a single mutually exclusive assignment as either a conceptual framework/model, a decision aid or decision-support tool, or a structured intervention, care pathway, or implementation strategy. The design categories, the detailed strata, and the cross-tabulations are provided in Supplementary Table S3.

### 2.9. Critical Appraisal

Consistent with the JBI methodology for scoping reviews [13,17], no formal risk-of-bias or study-level critical appraisal was performed, because the objective was to map the extent, range, characteristics, and representation of integrative decision-making approaches rather than to estimate comparative effectiveness. Accordingly, the frequency, coverage, or structural prominence of a framework category was not interpreted as evidence of its effectiveness, methodological quality, or clinical superiority.

## 3. Results

### 3.1. Overview of Included Studies

Of 2648 records identified from PubMed, 1144 underwent dual-reviewer title/abstract screening and 106 proceeded to 100% independent dual full-text assessment; 82 records were ultimately included (Figure 1). The false-exclusion rate in the pre-registered audit was 1/151 (0.66%; two-sided 95% Wilson upper bound 3.66%), below the pre-registered 5% acceptance threshold. Inter-rater agreement was 91.87% at Stage 1 (Cohen’s κ = 0.523; PABAK = 0.837) and 97.17% across the 106 full-text records before reconciliation (κ = 0.920; PABAK = 0.943). Full screening-stage statistics are provided in Supplementary File S5. The complete included-study list is provided in references [12,23,24,25,26,27,28,29,30,31,32,33,34,35,36,37,38,39,40,41,42,43,44,45,46,47,48,49,50,51,52,53,54,55,56,57,58,59,60,61,62,63,64,65,66,67,68,69,70,71,72,73,74,75,76,77,78,79,80,81,82,83,84,85,86,87,88,89,90,91,92,93,94,95,96,97,98,99,100,101,102,103]; references [23,24,25,26,27,28,29,30,31,32,33,34,35,36,37,38,39,40,41,42,43,44,45,46,47,48,49,50,51,52,53,54,55,56,57,58,59,60,61,62,63,64,65,66,67,68,69,70,71,72,73,74,75,76,77,78,79,80,81,82,83,84,85,86,87,88,89,90,91,92,93,94,95,96,97,98,99,100,101,102,103] are listed in reverse chronological order.

The 82 included records were published between 2010 and 2026 (median 2021) and represented heterogeneous study designs, settings, and treatment contexts. Detailed descriptive characteristics, including study design, care setting, centre status, treatment line, B/C/E dimensions, and integration depth, are summarised in Supplementary Table S4. The full record-level study list and mapped characteristics are provided in Supplementary Table S5 and the accompanying machine-readable dataset (Supplementary File S8). Integration depth was all-three axes in 22/82 records (26.8%), two-of-three in 33/82 (40.2%), and one axis centrally in 27/82 (32.9%). Lung cancer was the most frequently represented single predominant tumour type, while mixed-tumour cohorts were common; tumour-type representation is summarised separately in Supplementary Table S6, because predominant tumour type and representation of a tumour type anywhere in a mixed study population are distinct measures.

For the unit-of-analysis stratification, 24/82 records (29.3%) were randomised or cluster-randomised trials, 11/82 (13.4%) non-randomised interventional studies, 13/82 (15.9%) protocols, and 20/82 (24.4%) observational, qualitative, or mixed-methods studies; the remaining records comprised framework/model or Delphi papers, guidelines/consensus statements, systematic reviews, and other designs. Independently of study design, the primary mapped architecture was a conceptual framework/model in 14 records (17.1%), a decision aid or decision-support tool in 13 (15.9%), and a structured intervention, care pathway, or implementation strategy in 55 (67.1%). These dimensions were kept separate because the architecture under study and the study design used to develop or evaluate it are conceptually distinct. The complete distributions and their cross-tabulation are provided in Supplementary Table S3.

### 3.2. D-S-T × B/C/E Coverage Matrix

**Figure 2:**
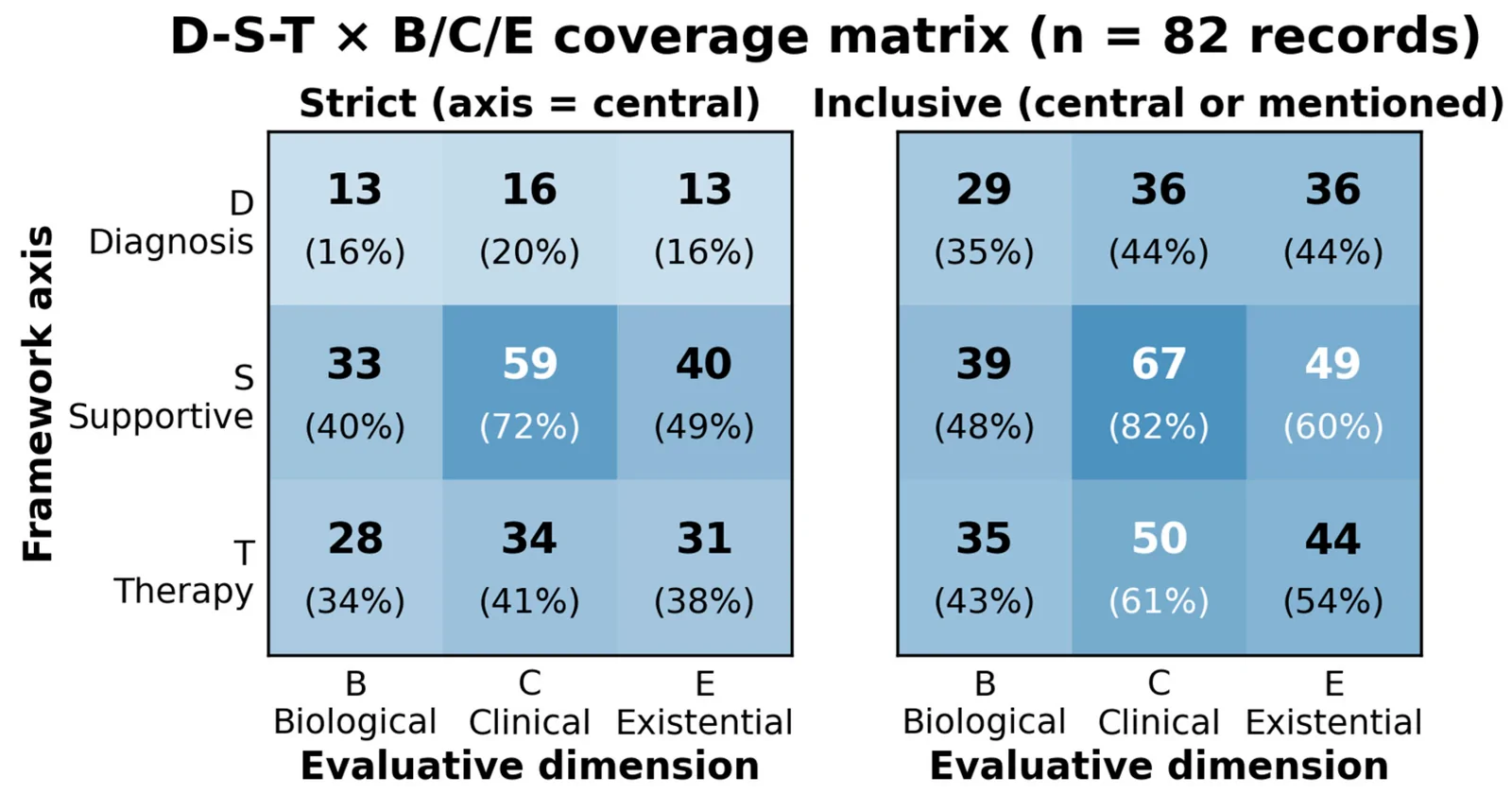
D-S-T × B/C/E coverage matrix (*n* = 82 records). (**Left**), strict analysis (Central only); (**right**), inclusive analysis (Central or Mentioned). Cells show the number and percentage of included records in which each D-S-T domain and B/C/E dimension were co-represented. Cell counts are based on reviewer-coded record-level classifications and do not represent quantities directly reported in the source studies. Percentages shown in the figure are rounded to the nearest whole number. D, Diagnosis; S, Supportive care; T, Therapy; B, Biological; C, Clinical; E, Existential. Darker shades of blue indicate higher percentages of included records in each cell.

Figure 2 shows the co-representation of the three decision/action domains and the B/C/E dimensions. The strict analysis counted only domains classified as Central, whereas the inclusive analysis also included those classified as Mentioned. Cell counts denote co-representation between a decision/action domain and a B/C/E dimension, not a causal decision-to-outcome relationship.

Under the strict definition (*n* = 82), Supportive care showed the greatest overall coverage, with S × C the most frequently represented cell (59/82, 72.0%). Diagnosis showed the lowest coverage, with D × B and D × E each represented in 13/82 records (15.9%). Under the inclusive definition, Diagnosis-related coverage increased but remained relatively under-represented compared with Supportive care and Therapy (Figure 2).

Inter-rater agreement was lower for Diagnosis than for Supportive care or Therapy, largely reflecting distinctions between Central and Mentioned. Agreement increased when these categories were collapsed for the inclusive analysis, and the strict and inclusive cell rankings were strongly concordant (Spearman’s ρ = 0.895). Full inter-rater statistics and sensitivity-analysis results are provided in Supplementary File S5.

### 3.3. Inductive Typology of Integrative Frameworks

**Figure 3:**
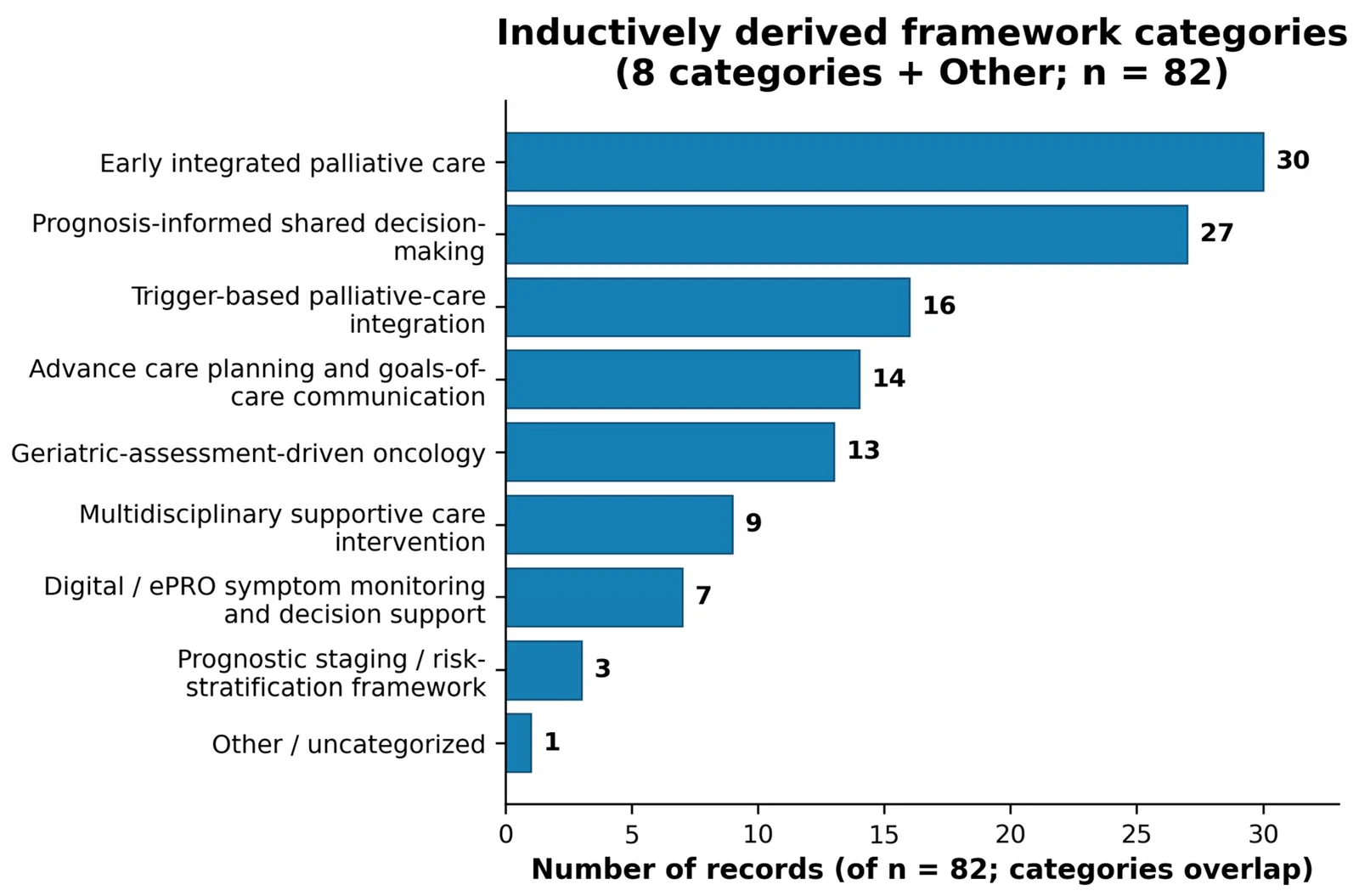
Inductively derived framework categories. Bars show the number of included records (*n* = 82) represented in each category. Categories are not mutually exclusive; each record could contribute to up to two categories, so percentages do not sum to 100%. Detailed definitions and mappings are provided in Supplementary Table S1 and Supplementary File S6.

During charting, records were assigned one or two free-text typology descriptors, yielding 160 raw labels across the original 80-record included set. AI-assisted consolidation of these charted descriptors into recurring framework categories, reviewed and finalised by the authors, mapped 158/160 labels (98.8%) to eight recurring framework categories (Figure 3; category definitions with example PMIDs are provided in Supplementary Table S1, and the full raw-to-category mapping in Supplementary File S6); 2/160 labels (1.3%) were assigned to a residual Other category. The two records added to the included set at revision consensus following the 100% independent dual full-text assessment had not been charted with free-text descriptors; their four category assignments were therefore made directly by K.M. against the finalised category definitions and are flagged as directly assigned in Supplementary File S6, which therefore comprises 160 label-to-category mappings plus four direct author assignments, 164 assignment rows in total; the 160 AI-consolidated labels were not re-mapped.

Early integrated palliative care was represented in 30 records (36.6%), followed by prognosis-informed shared decision-making in 27 records (32.9%). Trigger-based palliative-care integration was represented in 16 records (19.5%); advance care planning and goals-of-care communication in 14 records (17.1%); geriatric-assessment-driven oncology in 13 records (15.9%); multidisciplinary supportive care intervention in 9 records (11.0%); digital/ePRO symptom monitoring and decision support in 7 records (8.5%); and prognostic staging/risk-stratification framework in 3 records (3.7%). One record (1.2%) was assigned to the Other category. Framework categories were not mutually exclusive; each record could contribute to up to two categories, so percentages do not sum to 100%. Category definitions and representative studies are provided in Supplementary Table S1, and the complete record-level mapping is provided in Supplementary File S6.

### 3.4. Implementation Factors—CFIR Domain Coverage

**Figure 4:**
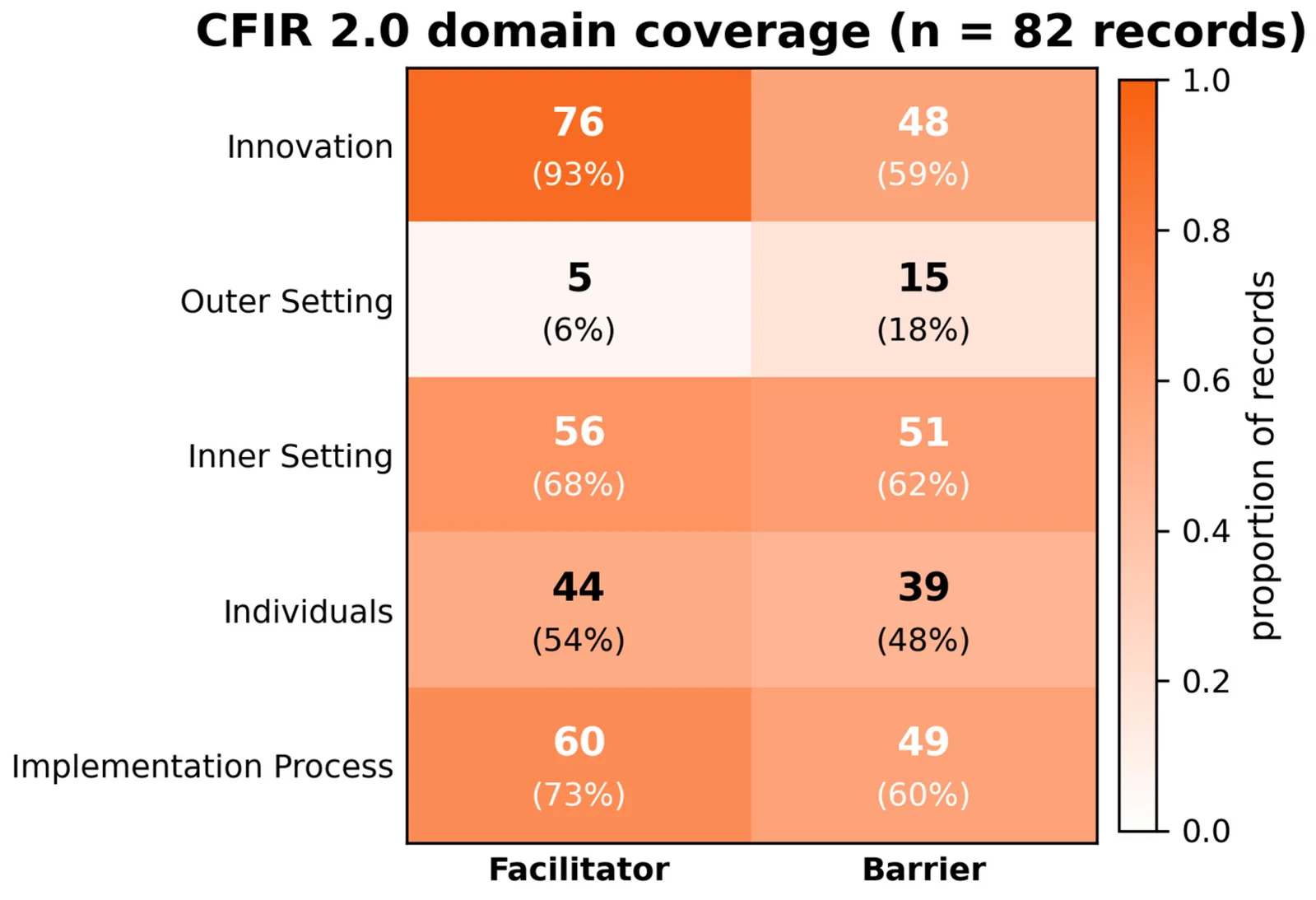
CFIR 2.0 domain coverage of reported facilitators and barriers after verification and adjudication (*n* = 82 records). Cells show the number and percentage of included records with a facilitator or barrier mapped to each CFIR domain; each record could contribute at most one hit per domain–role combination, so the aggregate of 443 counts record–domain–role instances across the five domains. Shading is the proportion of the 82 included records with a hit in that domain and role. CFIR, Consolidated Framework for Implementation Research.

Across the 82 included records, manual contextual CFIR mapping identified 443 record–domain–role instances (241 facilitators and 202 barriers); keyword matching was used only to retrieve candidate text [16]. Each record contributed at most one facilitator and one barrier per CFIR domain, so Figure 4 cells represent numbers of records rather than raw keyword frequencies. Independent verification of the random 16-record sample gave agreement on 116 of 160 binary classifications (72.5%; Cohen’s κ = 0.453; PABAK = 0.450). The 44 discordant classifications were adjudicated by K.I.; six additional instances were added to the final mapping and none was removed.

Innovation was the most frequently represented domain (76 facilitator and 48 barrier instances; 124 in total), followed by Implementation Process (60 and 49; 109), Inner Setting (56 and 51; 107), and Individuals (44 and 39; 83). Outer Setting remained least represented (5 and 15; 20) and was predominantly barrier-related. Detailed reliability results, final domain counts, and record-level evidence are provided in Supplementary Files S5 and S7.

### 3.5. Cross-Axis Coupling—Scoring and Highest-Scoring Records

Of the 82 included records, 22 (26.8%) met an emergence-candidate screen applying either of two conditions: charted integration depth of all-three, that is, all three D-S-T domains functionally integrated within the framework or care pathway (Section 2.7); or a positive all-three-dimensions outcome flag, the charted judgement that biological, clinical and existential outcomes were all reported. Both variables are charted fields in the registered protocol, but the screen combining them was defined at the synthesis stage and was not itself pre-registered. Integration depth was all-three for 22 records and the outcome flag was positive for 16; all 16 flagged records are among the 22, so the second condition selected no record the first had not, and the screen returns 22 records (Supplementary Table S2). Both conditions are reproducible per record, depth from Supplementary Table S5 and the outcome flag from the record-level dataset published with Table S5. This screen identified records for closer examination and does not, by itself, establish cross-axis coupling. The all-three-dimensions outcome flag should not be confused with the separate B, C and E presence flags: 32 of the 82 records carry all three presence flags, only 17 of which are also all-three on depth, and that presence combination was not used as a screening condition.

K.M. and Y.S. scored all 22 candidate records using the three-component cross-axis coupling rubric (Section 2.8). Exact agreement was 21/22 (95.5%) for explicit D-S-T linkage, 19/22 (86.4%) for triggering or reassessment, and 19/22 (86.4%) for longitudinality; the corresponding linear-weighted Cohen’s κ values were 0.909, 0.723, and 0.697. Total scores were identical for 17/22 records (77.3%) and were strongly rank-correlated (Spearman’s ρ = 0.916). Three records—Kaasa et al. (2018) [78], Smith et al. (2017) [85], and Temel et al. (2011) [102]—received the maximum total score of 6/6 from both reviewers and are presented below as the three tied highest-scoring records. The component scores, total scores, and supporting evidence for all 22 candidate records are provided in Supplementary Table S2.

Highest-scoring record 1|Kaasa et al., 2018 [78]—Lancet Oncology Commission.

The Commission linked objective prognostic assessment (D) with advance care planning and integrated palliative care (S) and with decisions on palliative chemotherapy and treatment discontinuation (T). Prognostic tools including PaP, PPI, mGPS, Feliu, and PiPS were incorporated alongside criteria for palliative-care referral and treatment reassessment; decisions to start, continue, or stop tumour-directed treatment were considered in relation to disease progression, performance status, nutritional decline, and symptom burden. The three axes were explicitly connected within a common reassessment pathway [78].

Highest-scoring record 2|Smith et al., 2017 [85]—ASCO guideline implementation through the TEAM approach.

The TEAM approach operationalises early concurrent palliative care alongside oncology care through dedicated time, formal symptom, psychosocial and spiritual assessment, and interdisciplinary management (S), together with structured recurrent discussion of prognosis (D) and repeated review of medically appropriate treatment options, goals of care, and advance directives (T). A worse scan or a change in ECOG performance status is used as the cue to revisit prognosis and realistic treatment options, so the three axes are connected within a recurring reassessment cycle [85].

Highest-scoring record 3|Temel et al., 2011 [102]—Longitudinal early integrated palliative care in metastatic non-small-cell lung cancer.

Early integrated palliative care began within three weeks of enrolment and continued at least monthly until death (S), and the palliative-care visit guidelines explicitly covered illness understanding and prognosis (D) and treatment decision-making, including the benefits and burdens of medical therapies (T). Prognostic perceptions were reassessed longitudinally, and a higher proportion of patients receiving early palliative care remained or became accurate that their cancer was incurable (82.5% versus 59.6%) [102].

The remaining candidate records showed varying degrees of explicit D-S-T linkage, triggering or reassessment, and longitudinality, with total scores ranging from 3 to 6, but did not receive the maximum score from both reviewers. Cross-axis coupling is therefore reported as a graded pattern across the candidate set rather than as a property that is simply present or absent, and the three tied highest-scoring records provide the clearest mapped examples of active linkage among Diagnosis, Supportive care, and Therapy.

## 4. Discussion

### 4.1. Principal Findings

This scoping review mapped 82 records addressing decision-making frameworks in advanced adult cancer care against an author-developed D-S-T × B/C/E conceptual mapping scaffold. Three descriptive findings summarise the map. First, coverage of the 3 × 3 D-S-T × B/C/E matrix is uneven: Supportive care is the most frequently central axis and Diagnosis the sparsest, with D × B and D × E the least represented cells. This asymmetry was robust under both strict and inclusive definitions. Second, despite the diversity of the charted framework descriptors, the literature converged on a small number of recurring framework categories, with early integrated palliative care and prognosis-informed shared decision-making being the two most frequently represented categories. Third, in the CFIR analysis (Section 3.4), reported implementation factors are dominated by Innovation characteristics, Implementation Process, Inner Setting, and Individuals; Outer Setting factors are uncommon and appear predominantly as barriers. Although inclusion of components classified as Mentioned increased cell counts and altered some rank positions, the overall nine-cell ordering remained strongly correlated across coding thresholds. Diagnosis-related combinations remained relatively under-represented. However, the precise magnitude of under-representation remains sensitive to the Central/Mentioned boundary, and the matrix should therefore be interpreted as a descriptive map of the reviewed literature rather than as a precise quantitative estimate of field-wide evidence gaps.

### 4.2. Comparison with the Existing Literature

The dominance of Supportive-care-centred frameworks and the scarcity of Diagnosis-centred frameworks are consistent with the historical trajectory of the field. Early integrated palliative care has been the principal engine of advanced-cancer decision-making research since the seminal Temel [104] and Bruera [10] contributions, with the Kaasa Lancet Oncology Commission [78] formalising the organisational model that many subsequent frameworks have extended. Prognostic-communication instruments and decision aids [75,101] are widely discussed but less frequently deployed as the central engine of an integrative framework. Our eight inductively derived framework categories (with a residual Other category) complement Kearney and colleagues’ call for a more person-centred conceptualisation of precision oncology [1]; we use the CFIR 2.0 framework of Damschroder and colleagues [16] to contextualise implementation factors, and the formal consolidation of charted descriptors into recurring categories has not, to our knowledge, been previously reported at this level of granularity.

The Outer-setting asymmetry—many barriers, few facilitators—is consistent with the wider implementation-science observation that integrative frameworks are typically designed inside healthcare institutions and encounter friction at the outer-setting interface with reimbursement systems, referral networks, and cultural or family-centred expectations. Frameworks that address outer-setting barriers proactively appear under-represented in the current evidence base and represent a legitimate priority for future intervention design. Table 1 sets the conceptual coverage of selected antecedent and cancer-care frameworks alongside that of the framework proposed in Section 4.4.

This comparison suggests that the novelty of the proposed Adaptive framework does not lie in multidimensionality itself. Biological, clinical, psychosocial, existential, and longitudinal perspectives are already represented in established models. Rather, the author-proposed contribution is the explicit organisation of Diagnosis, Supportive care, and Therapy as revisitable decision/action domains; the functional separation of B/C/E-related state or outcome signals, patient-reported or experiential outcomes, preferences/values, and process indicators; and a longitudinal, non-exclusive feedback structure in which changes in any B/C/E-related signal may prompt reassessment of one or more D/S/T domains. These proposed integrations remain hypothesis-generating and require prospective evaluation.

### 4.3. Implications for Practice, Policy, and Research

For practice, this review does not establish the comparative effectiveness or clinical superiority of any decision-making architecture. Rather, early integrated palliative care and prognosis-informed shared decision-making were the two most frequently represented approaches in the mapped literature and illustrate ways in which supportive-care and treatment deliberation have been structured in advanced-cancer care. Trigger-based palliative-care integration [12] and geriatric-assessment-driven oncology [11] were also represented as alternative organisational approaches. Whether any of these architectures improves patient outcomes, or whether one approach is preferable in a particular clinical setting, cannot be determined from the present scoping review and requires study-level quality appraisal and prospective comparative evaluation. The relative frequency of a framework category reflects its representation in the retrieved literature and should not be interpreted as evidence that the category is clinically more effective or preferable to less frequently represented approaches.

For policy, the Outer Setting barriers mapped in the CFIR analysis highlight contextual factors that may warrant consideration when adapting or implementing such approaches, including reimbursement structures, referral pathways, and cultural or family-centred communication practices. However, the present review does not establish that modification of these factors improves implementation success or clinical outcomes.

For research, a distinctive descriptive finding of this review is that, within the 22-record candidate set, three records received the maximum cross-axis coupling score of 6/6 from both reviewers, with the D, S, and T axes explicitly linked within a common reassessment pathway rather than merely co-present. This small highest-scoring subset motivates—but does not test—a framework hypothesis worth prospective investigation, which we refer to as the cross-axis coupling hypothesis: integrative frameworks in which one decision axis actively triggers reassessment of at least one other D-S-T axis may produce measurably different outcomes on one or more of the B/C/E dimensions than frameworks in which the axes coexist without such triggering. Distinguishing passive integration from active cross-axis coupling is not resolvable within a scoping review. In parallel, the two under-represented cells of the D-S-T × B/C/E matrix—Diagnosis × Biological and Diagnosis × Existential—are candidate priority areas for future intervention research, particularly for interventions that couple prognostic instruments or biomarker profiling with survival or dignity-and-meaning endpoints.

The transferability of the mapped decision-making architectures across cancer types should be interpreted cautiously. The evidence base was disproportionately represented by lung cancer, with breast cancer also recurring across both disease-specific and mixed-tumour studies. By contrast, haematological malignancies were represented by relatively few dedicated studies and appeared mainly within mixed cohorts. CNS malignancies were sparsely represented, largely within mixed or consensus-based records rather than dedicated cohorts. Gastrointestinal and pancreatic cancers were more substantially represented, including dedicated studies of pancreatic, gastro-oesophageal, and colorectal cancer, but the transferability of decision-making structures across these diseases may still be influenced by disease-specific trajectories, symptom burden, prognosis, and treatment options. Rare cancers, including sarcoma, mesothelioma, melanoma, and other uncommon tumour types, were generally represented by isolated studies or as small components of mixed cohorts. Accordingly, the D-S-T × B/C/E patterns identified here should be regarded as a map of the available literature rather than as a cancer-type-independent architecture. Disease-specific validation will be particularly important in haematological malignancies, CNS tumours, rare cancers, and other populations that were sparsely represented or outside the review design. This limitation also applies to populations excluded by design, including paediatric patients and predominantly curative-intent or early-stage cancer populations, for whom the present mapping should not be assumed to apply directly.

### 4.4. Toward an Adaptive D-S-T × BCE Framework

Building on the descriptive findings above, we propose an integrative framework that operationalises the cross-axis coupling hypothesis for prospective testing. The framework is not derived de novo; rather, it is a synthesis of the recurrent architectural elements identified across the eight recurring framework categories (Section 3.3), the three tied highest-scoring emergence-candidate records (Section 3.5 [78,85,102]), and the CFIR-mapped implementation factors described in Section 3.4. We present it here as a candidate hypothesis for prospective testing rather than as a validated instrument, consistent with the scoping-review methodology adopted throughout [13,15].

The proposed Adaptive D-S-T × BCE Framework should be distinguished from the descriptive findings of this scoping review. Literature-derived components include the recurrent representation of Diagnosis, Supportive care, and Therapy domains; the Biological, Clinical, and Existential dimensions identified in the mapped literature; recurring architectures such as early palliative-care integration, geriatric-assessment-guided care, shared decision-making, and trigger-based reassessment; and implementation determinants mapped using CFIR. Author-proposed components include the integration of these elements into a single longitudinal Adaptive D-S-T × BCE architecture, the use of B/C/E dimensions as feedback signals for reassessment, the proposed monitoring structure, and the extension of multidomain host assessment to all adults with advanced cancer.

B/C/E are retained as broad evaluative dimensions for mapping purposes but are not treated as a homogeneous set of outcomes or feedback signals in the proposed Adaptive framework. Information within these dimensions is separated functionally into four classes: (i) biological and clinical state or outcome signals; (ii) patient-reported or experiential outcomes; (iii) preferences and values, which primarily serve as decision inputs; and (iv) communication or care-process indicators. Within the E dimension, which is explicitly subdivided into three conceptually distinct components, existential well-being (e.g., dignity, meaning, hope, or existential distress) is distinguished from preference and value inputs and from communication/process constructs such as prognostic communication, ACP/GOC, and goal concordance. Constructs grouped within the same broad B/C/E dimension may therefore play different roles in decision-making and should not be interpreted as equivalent outcomes.

#### 4.4.1. Two Organising Principles

**Figure 5:**
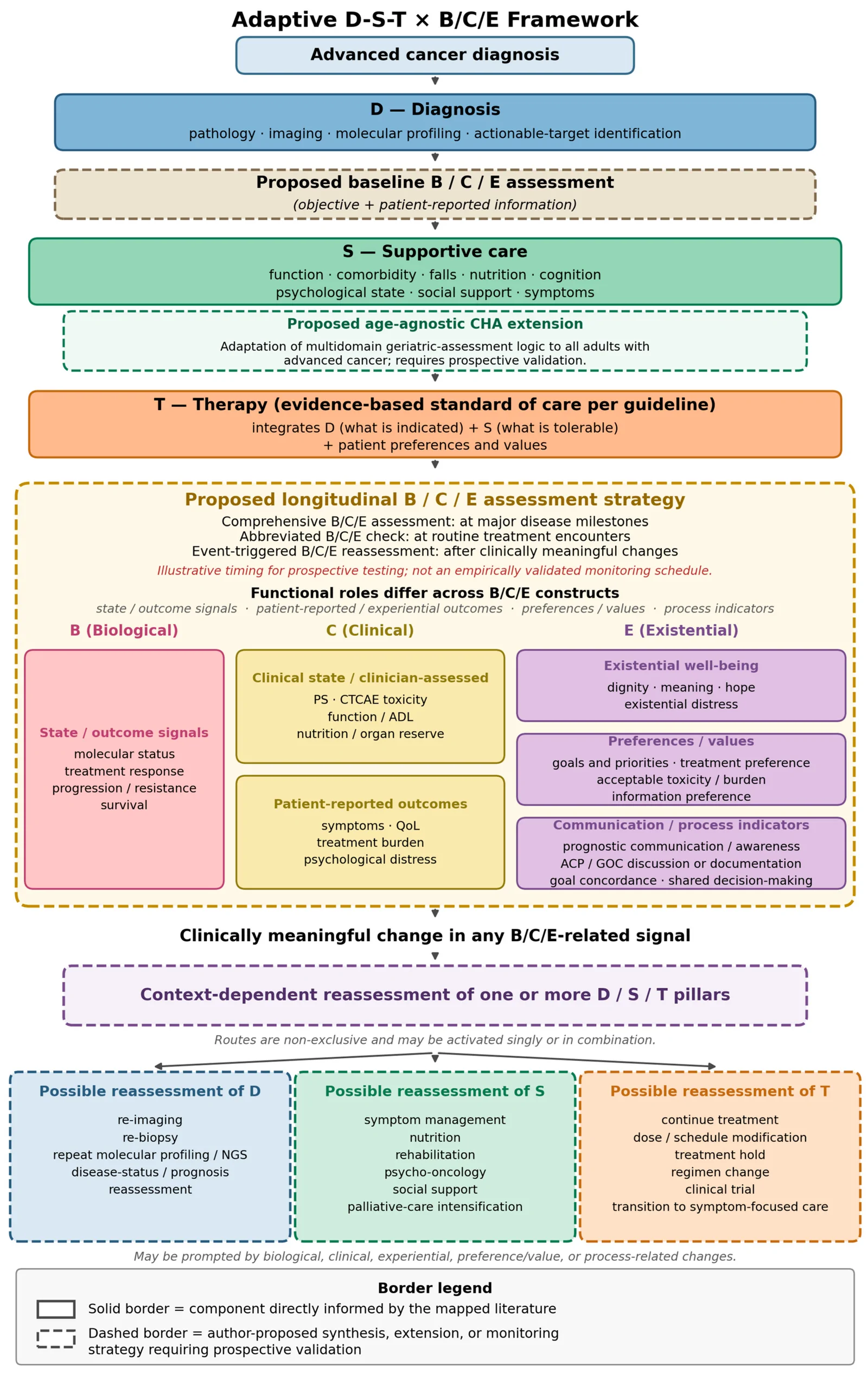
Adaptive D-S-T × B/C/E Framework for advanced cancer. The author-proposed framework links revisitable D/S/T decision/action pillars with functionally differentiated B/C/E information through non-exclusive, context-dependent reassessment routes. Solid borders indicate components directly informed by the mapped literature; dashed borders indicate author-proposed extensions. B/C/E information is distinguished as state/outcome signals, patient-reported or experiential outcomes, preferences/values, and process indicators; within E, existential well-being is separated from preference/value inputs and from ACP/GOC-related process indicators. The proposed assessment timing and the age-agnostic Comprehensive Host Assessment are illustrative and require prospective evaluation. ACP, advance care planning; ADL, activities of daily living; B/C/E, biological/clinical/existential; CHA, Comprehensive Host Assessment; CTCAE, Common Terminology Criteria for Adverse Events; D, Diagnosis; GOC, goals of care; NGS, next-generation sequencing; PS, performance status; QoL, quality of life; S, Supportive care; T, Therapy.

The proposed framework, which we refer to as the Adaptive D-S-T × BCE Framework (Figure 5), rests on two organising principles. First, D (Diagnosis), S (Supportive care), and T (Therapy) are revisitable decision/action pillars across the advanced-cancer trajectory; the framework does not replace guideline-based treatment selection but structures how the three pillars are re-opened over time. Second, the broad B/C/E dimensions provide functionally differentiated information that may prompt reassessment of one or more D/S/T pillars over time; they are not treated as homogeneous outcomes or as fixed one-to-one feedback signals.

#### 4.4.2. Structure

The framework proceeds longitudinally from advanced-cancer diagnosis, beginning with a baseline assessment that combines objective and patient-reported information across the B/C/E dimensions. Within the S (Supportive care) pillar we propose a Comprehensive Host Assessment (CHA), informed by multidomain geriatric-oncology assessment developed and evaluated primarily in older adults [11], spanning function, comorbidity, falls, cognition, psychological status, nutrition, polypharmacy, and social support. Extending this multidomain approach to adults of all ages is an author-proposed, age-agnostic adaptation requiring prospective evaluation. Therapy decisions would integrate disease-specific evidence (D), patient-level supportive-care and fitness considerations (S), and the preferences and values captured in the B/C/E assessment.

For prospective testing, B/C/E information could be reassessed comprehensively at major disease milestones, through a proposed abbreviated B/C/E check at routine treatment encounters, and through event-triggered reassessment after clinically meaningful unplanned changes, drawing on the electronic patient-reported outcome alert logic described by Basch and colleagues [105]. These assessment intensities and intervals are illustrative and do not represent validated instruments or established monitoring schedules.

#### 4.4.3. Non-Exclusive Feedback Routes

Changes in B/C/E-related information may prompt reassessment of one or more D/S/T pillars depending on clinical context. Biological or clinical change may lead to diagnostic or prognostic reassessment, therapy modification, and/or supportive-care review, whereas changes in existential well-being, preferences, or goals may alter treatment choices, supportive-care needs, or the amount and type of prognostic information desired. The feedback routes are therefore non-exclusive and context-dependent rather than fixed one-to-one mappings.

#### 4.4.4. Position Within the Field

The proposed framework does not introduce a new individual clinical component; rather, it combines elements already represented across the mapped literature into an explicit longitudinal coupling architecture. Its added value is therefore a testable conceptual organisation of existing elements, not evidence of clinical superiority.

#### 4.4.5. Novelty of the Proposed Adaptive Framework

The proposed novelty lies in the combination of five features: (1) revisitable D/S/T decision/action pillars; (2) functionally differentiated B/C/E information; (3) context-dependent, non-exclusive reassessment triggers; (4) explicit separation of existential well-being from preference and value inputs and from ACP/GOC or goal-concordance process indicators; and (5) recurrent longitudinal reassessment rather than a static architecture. These features constitute an author-proposed conceptual synthesis requiring prospective evaluation.

### 4.5. Strengths and Limitations

A further limitation concerns the review team itself: the authors are the originators of the D-S-T × B/C/E framework against which the literature was mapped, so the selection of the mapping scaffold, the axis classification rules and the interpretation of the resulting map are not independent of the framework being examined. The pre-registered criterion, the blinded dual screen, the adjudication arrangement and the published record-level data are intended to make each step checkable, but they do not remove this conflict, and the map should be read with it in mind. Strengths include pre-registration prior to database searching, a dual-reviewer workflow with independent adjudication and pre-registered acceptance thresholds for the AI pre-filter, transparent reporting of inter-rater reliability, and public availability of the registered protocol and core analysis materials on OSF, together with the full 82-record dataset in the Supplementary Materials. Reporting followed PRISMA-ScR [18].

Limitations. First, the final evidence set was derived from a single native bibliographic database search in PubMed. This is a major limitation for a multidisciplinary topic: relevant literature indexed elsewhere may have been missed, and the PubMed-based proxy scans could not assess coverage outside PubMed. Several supplementary search procedures specified in the protocol, including backward and forward citation searching, were not completed as planned. In addition, combining four concept blocks with AND may have reduced sensitivity for relevant reports that did not use terminology from every block. The findings should therefore be interpreted as a map of the captured literature, not an exhaustive synthesis of all relevant literature. Deviations from the registered information-source plan are documented in Supplementary File S1. Second, the English/Japanese restriction may have limited frameworks from other linguistic, cultural, and healthcare-system contexts, especially preference-sensitive, communication-related, and existential constructs. Third, the AI-assisted pre-filter may have introduced residual selection bias despite the preregistered blinded human audit. A low observed false-exclusion rate does not exclude systematic errors affecting particular terminology, study designs, publication styles, or under-represented conceptual studies. Fourth, the review intentionally includes heterogeneous units of evidence—conceptual frameworks, decision aids, structured interventions, care pathways, protocols, observational studies, randomised trials, and guidelines. Even after stratification by study design and primary mapped architecture, these are not methodologically equivalent and do not carry equal evidentiary weight. Fifth, consolidation of charted descriptors into inductively derived framework categories remains interpretive. Categories overlap, are not externally validated, and may be sensitive to alternative coding decisions. D/S/T Central/Mentioned/Absent and integration-depth judgements also retain interpretive elements despite coding rules, inter-rater assessment, and sensitivity analysis. Inter-rater reliability across the four core charting blocks was substantial to almost-perfect (interpretable per Landis and Koch [106]): Cohen’s kappa 0.871 for the Biological block (PABAK 0.875), 0.636 for the Clinical block with strong prevalence imbalance (PABAK 0.875), 1.000 for the Existential block, and 0.904 for the three-category Depth classification used in the emergence-candidate screen (PABAK 0.906). The only block with fair reliability was the all-three-dimensions outcome flag (Cohen’s kappa 0.333 after schema normalisation), reflecting a definitional ambiguity in the pre-registered field guide specific to that flag. It does not affect Figure 2 or the inductively derived framework categories (Supplementary Table S1), but it is one of the two conditions of the Section 3.5 screen, so its reliability bears on the candidate set: because every flagged record is also all-three on depth, the 22 candidates stand on the depth condition alone, and adopting the second reviewer’s value for each of the five flag discordances would add three records and give 25 rather than 22. The three tied highest-scoring records are all-three on depth and are unaffected. This sensitivity analysis is set out in Supplementary File S5. Sixth, cross-axis coupling remains exploratory. The 22 candidate records were evaluated using a structured three-component rubric applied by K.M. and Y.S., with reviewer agreement quantified for each component and for total scores. Although concordance was high, the rubric was developed during peer-review revision and has not been externally validated, so the scores should be interpreted as a structured descriptive synthesis rather than as a validated measurement instrument. Seventh, CFIR mapping involves interpretive judgement. In the 16-record verification sample, pre-reconciliation agreement across the 160 domain–role classifications was 72.5% (Cohen’s κ = 0.453; PABAK = 0.450), with variation between individual domains, and all discordances were adjudicated before the final mapping was produced; the CFIR analysis should nevertheless be read as descriptive implementation mapping rather than as a validated measurement instrument. Eighth, no formal study-level risk-of-bias or critical-appraisal assessment was performed, consistent with JBI scoping-review methodology [13]. Frequency or structural prominence in the map does not establish comparative effectiveness, methodological quality, or clinical superiority. Ninth, cancer-type representation was uneven. Lung and breast cancer contributed prominently; haematological malignancies, CNS tumours, and several rare cancers were sparsely represented. Transferability to these groups, and to paediatric and predominantly curative-intent populations excluded by design, remains uncertain. Tenth, the review spans 2010–2026, during which cancer therapeutics, molecular diagnostics, immunotherapy, supportive care, digital monitoring, and communication practice changed substantially. Temporal heterogeneity may affect apparent similarity across frameworks. Eleventh, the search strategies were documented against the PRESS 2015 checklist [107] by the review team but were not independently peer-reviewed by an information specialist. Finally, the proposed Adaptive D-S-T × B/C/E Framework is an author-developed conceptual synthesis. Its proposed monitoring structure, non-exclusive feedback pathways, age-agnostic host-assessment extension, and B/C/E functional subdivisions are not prospectively validated and require formal development, feasibility testing, measurement validation, and prospective comparative evaluation before clinical implementation.

## 5. Conclusions

This scoping review identified patterns in how diagnostic, supportive-care, and therapeutic decision-making architectures are represented across the advanced-cancer literature. The mapped literature showed uneven D-S-T × B/C/E coverage, with Supportive-care-centred architectures more frequently represented and Diagnosis-centred combinations relatively under-represented within the reviewed evidence base. These findings describe patterns of representation and conceptual organisation; they do not establish comparative effectiveness, clinical superiority, or evidence that the proposed Adaptive D-S-T × B/C/E Framework improves patient outcomes.

The proposed Adaptive framework should therefore be regarded as a hypothesis-generating conceptual model derived from the mapped literature and requiring formal development, feasibility testing, psychometric validation of its proposed assessment components, and prospective comparative evaluation before clinical implementation.

## Figures and Tables

**Table 1:** Author-mapped comparison of conceptual coverage between selected antecedent and cancer-care frameworks and the proposed Adaptive D-S-T × B/C/E Framework.

Framework	D	S	T	B	C	E	Longitudinality	Feedback/Reassessment
Engel biopsychosocial model [6]	○	○	○	●	●	○	—	No explicit operational reassessment loop
Saunders Total Pain concept [7]	—	●	—	○	●	●	○	Holistic reassessment implicit; no structured cross-domain trigger
Wagner Chronic Care Model [8]	○	●	○	○	●	○	●	Iterative care supported by clinical information and follow-up systems
WHO palliative-care framework [9]	○	●	○	○	●	●	●	Continuity across the care continuum; no explicit D/S/T coupling rule
Bruera & Hui cancer-trajectory model [10]	○	●	○	○	●	●	●	Supportive/palliative intensity changes across the trajectory; no explicit cross-domain trigger
Geriatric-assessment-guided oncology/Mohile et al. [11]	○	●	●	○	●	○	○	Assessment findings inform supportive interventions and cancer-treatment modification
Villalobos milestone-based communication framework [75]	●	●	●	○	●	●	●	Milestone-triggered reassessment of prognosis, care goals, palliative care, and treatment strategy
**Proposed Adaptive D-S-T × B/C/E Framework**	●	●	●	●	●	●	●	Author-proposed non-exclusive feedback: changes in any B/C/E-related signal may prompt reassessment of one or more D/S/T domains

D, Diagnosis; S, Supportive care; T, Therapy; B, Biological; C, Clinical; E, Existential. ● explicit/core; ○ partial/indirect/contextual; —not a primary component. Each cell was assigned after review of the cited source rather than inferred from the present manuscript. This table represents an author-mapped qualitative comparison of conceptual coverage and is not a validated scoring system, evidence hierarchy, or assessment of comparative clinical effectiveness.

## Data Availability

The charting workbook for the 80 records charted before revision, the screening decision logs, the AI pre-filter audit results, the inter-rater reliability statistics, the framework-category mappings, and the analysis scripts are available in the OSF project at https://osf.io/b8qn9/ (accessed on 22 September 2026), registration DOI 10.17605/OSF.IO/ZKD53. All 32 charted fields for all 82 included records, together with the primary mapped architecture and the derived tumour-type variables, are published per record in Supplementary File S8. The workbook deposited on OSF covers the original 80 records and does not contain the primary mapped architecture column; the complete record-level dataset is provided in Supplementary File S8.

## Supplementary Materials

The following supporting information can be downloaded at: https://www.mdpi.com/article/10.3390/cancers18193245/s1, File S1: Database search strategies; File S2: PRISMA-ScR 22-item checklist; File S3: Charting form and field definitions, including the operational rules for the D/S/T axis classification and the CFIR mapping procedure; File S4: AI pre-filter pre-registered specification and implemented procedure; File S5: Inter-rater reliability report for the screening stages, the charting field blocks, the cross-axis coupling scoring and the CFIR verification, with the related adjudication and sensitivity analyses; File S6: Label-to-category mapping—160 AI-consolidated raw labels plus four assignments made directly by K.M. (CSV, 164 category-assignment rows); File S7: CFIR per-record hits with matched terms or adjudication provenance and charted evidence excerpts (CSV, 443 final record–domain–role instances across all 82 records); File S8: Machine-readable record-level dataset for the 82 included records (CSV; all 32 charted fields per record, plus the primary mapped architecture and the derived tumour-type variables); Table S1: Inductively derived framework categories with definitions and example PMIDs; Table S2: Cross-axis coupling analysis of the 22 emergence-candidate records—K.M. and Y.S. component scores, total scores, and supporting D/S/T and coupling evidence; Table S3: Unit-of-analysis stratification of the 82 included studies by study design and primary mapped architecture; Table S4: Descriptive characteristics of the included studies by variable category; Table S5: Full record-level table for the 82 included studies; Table S6: Tumour-type representation of the included studies—predominant type and representation anywhere in the study population. Earlier versions of the supplementary set are deposited in the OSF project (DOI 10.17605/OSF.IO/ZKD53) under the numbering used at initial submission; the revised versions submitted with this article are not yet deposited there.
