# Supplementary material for: Mapping Integrative Decision-Making Frameworks in Advanced Cancer Care: A Scoping Review of Diagnostic, Supportive-Care, and Therapeutic Integration
Source: Cancers (Basel). 2026 Oct 8;18(19):3245. doi: 10.3390/cancers18193245 (PMC13664350; doi:10.3390/cancers18193245)
Supplement: Supplementary file 1 [file cancers-18-03245-s001.zip › File S1_Search_strategies_v20.pdf]

Supplementary File S1

Database search strategies

DST Scoping Review · OSF DOI 10.17605/OSF.IO/ZKD53

Part 1 — June 2026 primary search and complementary scans used in the final review

1. PubMed primary search (registered source label: “MEDLINE via PubMed”)

Search executed: 21 June 2026

The final search combined four concept clusters joined by AND:

Cluster 1 — Population (advanced/metastatic adult cancer, exact as executed):
(((cancer[tiab] OR neoplasm*[tiab] OR malignan*[tiab] OR tumor*[tiab] OR tumour*[tiab] OR carcinoma*[tiab] OR lymphoma*[tiab] OR leukemia*[tiab] OR myeloma*[tiab] OR sarcoma*[tiab] OR "Neoplasms"[Mesh]) AND (advanced[tiab] OR metastatic[tiab] OR incurable[tiab] OR refractory[tiab] OR relapsed[tiab] OR "stage IV"[tiab] OR "stage 4"[tiab] OR end-stage[tiab] OR "end stage"[tiab] OR progressive[tiab] OR "stem cell transplantation"[tiab] OR "Hematopoietic Stem Cell Transplantation"[Mesh] OR HSCT[tiab])) OR "Neoplasm Metastasis"[Mesh])

Cluster 2 — Treatment (anticancer pharmacotherapy, exact as executed):
(pharmacotherapy[tiab] OR "systemic therapy"[tiab] OR "drug therapy"[tiab] OR "targeted therapy"[tiab] OR "molecular targeted therapy"[Mesh] OR immunotherapy[tiab] OR "immune checkpoint"[tiab] OR "antibody-drug conjugate"[tiab] OR "bispecific antibody"[tiab] OR chemotherapy[tiab] OR antineoplastic[tiab] OR anti-cancer[tiab] OR anticancer[tiab] OR "Antineoplastic Agents"[Mesh] OR "stem cell transplantation"[tiab] OR "Hematopoietic Stem Cell Transplantation"[Mesh] OR HSCT[tiab] OR "cancer treatment"[tiab] OR "cancer therapy"[tiab])

Cluster 3 — Intervention (palliative / supportive / integrative care, exact as executed):
("palliative care"[Mesh] OR "palliative care"[tiab] OR "Hospice Care"[Mesh] OR "Terminal Care"[Mesh] OR "Palliative Medicine"[Mesh] OR "supportive care"[tiab] OR "best supportive care"[tiab] OR "integrated care"[tiab] OR "concurrent care"[tiab] OR "simultaneous care"[tiab] OR "shared care"[tiab] OR "comprehensive geriatric assessment"[tiab] OR "Geriatric Assessment"[Mesh] OR "early integrated palliative care"[tiab] OR "specialty palliative care"[tiab] OR "outpatient palliative care"[tiab] OR "biomarker reassessment"[tiab] OR "end-of-life care"[tiab] OR hospice[tiab] OR "stepped care"[tiab] OR "stepped palliative care"[tiab])

Cluster 4 — Methodological framing (exact as executed):
(framework[tiab] OR model[tiab] OR pathway[tiab] OR intervention[tiab] OR implementation[tiab] OR component*[tiab] OR facilitat*[tiab] OR barrier*[tiab] OR decision-making[tiab] OR "decision making"[tiab] OR approach[tiab] OR strategy[tiab] OR strategies[tiab] OR process[tiab])

Combined: #1 AND #2 AND #3 AND #4

Rationale for the four-block structure. The four clusters were combined with AND so that retrieval was focused on records explicitly situated at the intersection of advanced cancer, cancer-directed treatment, integrative or supportive care, and a structured framework, model, pathway, or decision-making process, rather than on the broader advanced-cancer literature. Population and Treatment together fix the clinical situation, Intervention fixes the integrative care context, and Methodological framing restricts retrieval to records that describe a decision structure. The cost of that choice is recorded as a limitation in Section 4.5 of the main text: requiring all four blocks may have reduced sensitivity for conceptual reports that do not use one or more of these vocabularies.

Filters applied at the E-utility layer: publication date ("2010/01/01"[PDAT] : "2026/06/21"[PDAT]); language (English[Lang] OR Japanese[Lang]); publication-type exclusions NOT ("case reports"[pt] OR editorial[pt] OR comment[pt] OR letter[pt]). Retrieved 2,648 records. Source of truth: rayyan_phase/search_queries/pubmed_query.txt (2026-06-21 12:50:52) and rayyan_phase/logs/esearch_count_2026-06-21.json (PubMed E-utility response, count = 2,648).

Copy-pastable query as executed (clusters 1–4 combined with AND, then the filters above appended):

```
((((cancer[tiab] OR neoplasm*[tiab] OR malignan*[tiab] OR tumor*[tiab] OR tumour*[tiab] OR carcinoma*[tiab] OR
lymphoma*[tiab] OR leukemia*[tiab] OR myeloma*[tiab] OR sarcoma*[tiab] OR "Neoplasms"[Mesh]) AND (advanced[tiab]
OR metastatic[tiab] OR incurable[tiab] OR refractory[tiab] OR relapsed[tiab] OR "stage IV"[tiab] OR "stage
4"[tiab] OR end-stage[tiab] OR "end stage"[tiab] OR progressive[tiab] OR "stem cell transplantation"[tiab] OR
"Hematopoietic Stem Cell Transplantation"[Mesh] OR HSCT[tiab])) OR "Neoplasm Metastasis"[Mesh])) AND
((pharmacotherapy[tiab] OR "systemic therapy"[tiab] OR "drug therapy"[tiab] OR "targeted therapy"[tiab] OR
"molecular targeted therapy"[Mesh] OR immunotherapy[tiab] OR "immune checkpoint"[tiab] OR "antibody-drug
conjugate"[tiab] OR "bispecific antibody"[tiab] OR chemotherapy[tiab] OR antineoplastic[tiab] OR anti-cancer[tiab]
OR anticancer[tiab] OR "Antineoplastic Agents"[Mesh] OR "stem cell transplantation"[tiab] OR "Hematopoietic Stem
Cell Transplantation"[Mesh] OR HSCT[tiab] OR "cancer treatment"[tiab] OR "cancer therapy"[tiab])) AND
(("palliative care"[Mesh] OR "palliative care"[tiab] OR "Hospice Care"[Mesh] OR "Terminal Care"[Mesh] OR
"Palliative Medicine"[Mesh] OR "supportive care"[tiab] OR "best supportive care"[tiab] OR "integrated care"[tiab]
OR "concurrent care"[tiab] OR "simultaneous care"[tiab] OR "shared care"[tiab] OR "comprehensive geriatric
assessment"[tiab] OR "Geriatric Assessment"[Mesh] OR "early integrated palliative care"[tiab] OR "specialty
palliative care"[tiab] OR "outpatient palliative care"[tiab] OR "biomarker reassessment"[tiab] OR "end-of-life
care"[tiab] OR hospice[tiab] OR "stepped care"[tiab] OR "stepped palliative care"[tiab])) AND ((framework[tiab] OR
model[tiab] OR pathway[tiab] OR intervention[tiab] OR implementation[tiab] OR component*[tiab] OR facilitat*[tiab]
OR barrier*[tiab] OR decision-making[tiab] OR "decision making"[tiab] OR approach[tiab] OR strategy[tiab] OR
strategies[tiab] OR process[tiab])) AND ("2010/01/01"[PDAT] : "2026/06/21"[PDAT]) AND (English[Lang] OR
Japanese[Lang]) NOT ("case reports"[pt] OR editorial[pt] OR comment[pt] OR letter[pt])
```

2. Cochrane Database of Systematic Reviews (CDSR) — proxy

Executed as PubMed-linked syntax targeting Cochrane-indexed records already present in the primary PubMed set. 30 records identified; all were already present in that set (0 unique). This is a coverage estimator using a PubMed-based journal proxy, not a search of CDSR through its own interface; the absence of unique records therefore does not demonstrate independent coverage of that database.

Verbatim query as executed, transcribed from Supplementary File 2 of the registered protocol (section 4). The protocol's <EXECUTION_DATE> placeholder was replaced by 2026/06/21 at execution; the 21 June 2026 execution log records that the query run matched this file verbatim apart from that substitution:

```
((Cochrane Database Syst Rev[Journal]) AND ((cancer[tiab] OR neoplasm*[tiab] OR malignan*[tiab] OR tumor*[tiab])
AND (advanced[tiab] OR metastatic[tiab] OR incurable[tiab] OR "stem cell transplantation"[tiab] OR HSCT[tiab]) AND
("palliative care"[tiab] OR "supportive care"[tiab] OR "integrated care"[tiab] OR "comprehensive geriatric
```

```
assessment"[tiab] OR "end-of-life care"[tiab] OR hospice[tiab] OR "stepped care"[tiab])) AND ("2010/01/01"[PDAT] : "2026/06/21"[PDAT]))
```

3. CINAHL coverage proxy via a pre-specified PubMed journal filter

334 candidates identified via the pre-specified PubMed journal filter; all were already present in the primary PubMed set (0 unique). As for CDSR, this is a PubMed-subset estimator rather than a native CINAHL search.

Verbatim journal filter as executed, transcribed from Supplementary File 4 of the registered protocol (section 4.1); the identical filter appears in Supplementary File 1 of the same protocol (section 6). It was combined with the four-cluster query of Section 1 above by AND:

```
("Eur J Oncol Nurs"[Journal] OR "Cancer Nurs"[Journal] OR "Oncol Nurs Forum"[Journal] OR "J Pain Symptom Manage"[Journal] OR "Palliat Med"[Journal] OR "J Palliat Med"[Journal] OR "J Palliat Care"[Journal] OR "Am J Hosp Palliat Care"[Journal] OR "Palliat Support Care"[Journal] OR "BMJ Support Palliat Care"[Journal] OR "Support Care Cancer"[Journal] OR "Palliat Care Soc Pract"[Journal] OR "Int J Palliat Nurs"[Journal] OR "J Hosp Palliat Nurs"[Journal] OR "J Geriatr Oncol"[Journal] OR "J Adv Nurs"[Journal] OR "Nurs Outlook"[Journal])
```

The 17 journals were fixed before execution by a two-part rule recorded in the protocol: a candidate journal had to be (a) primarily a nursing journal, or a palliative-care or supportive-care journal, and (b) dual-indexed in CINAHL and MEDLINE according to the NLM Catalog. The filter therefore reaches only the CINAHL–MEDLINE overlap; journals unique to CINAHL are out of its reach by construction.

4. ClinicalTrials.gov (trial-registry complement)

Search terms mirrored Cluster 1 + Cluster 3 above; 14 registered studies identified. Records feeding into the pool were those with completed protocol registrations that yielded a published framework/protocol paper. Result after PMID de-duplication against the primary PubMed set: 0 unique records.

5. OpenAlex (grey-literature top-page sample)

Top-200 relevance-ranked records for the combined query "advanced cancer AND decision framework AND (palliative OR supportive) AND (shared decision OR prognostic)". Records already present in the primary PubMed set were de-duplicated at Rayyan import. Result after PMID de-duplication against that set: 0 unique records.

6. Registered information sources and the sources actually executed

The registered protocol lists ten information sources. The table below states, for each, what the 21 June 2026 execution log records. It is included so that the registered plan and the executed search can be compared directly.

#	Registered information source	Status at the 21 June 2026 run	Records
1	MEDLINE via PubMed (registered label)	Executed in PubMed as registered; because no medline[sb] restriction was applied, the retrieved set is described in the revised manuscript as the primary PubMed set rather than a MEDLINE-only set	2,648
2	Cochrane Library (CDSR + CENTRAL)	CDSR executed as the registered PubMed-journal proxy. CENTRAL not executed directly; the protocol pre-specifies ClinicalTrials.gov as its surrogate (source 7)	30
3	Web of Science Core Collection (registered as a forward-citation plus Semantic Scholar proxy where direct credentials are unavailable)	Not executed at this run: the forward-citation component was not run and Semantic Scholar returned no records (source 6). Native Web of Science was searched only at the revision stage (Part 2), not incorporated into the final screened evidence set	0
4	CINAHL	Executed as the registered 17-journal PubMed proxy	334

5	OpenAlex API	Partially executed: a top-200 relevance sample of 8,002 matching records. The remaining 7,802 were not retrieved	200
6	Semantic Scholar API	Attempted and failed: HTTP 429 rate limiting returned no records. The log notes a retry for a later session; no retry is recorded	0
7	ClinicalTrials.gov API v2	Executed as registered	14
8	Forward-citation tracking via PubMed pubmed_pubmed_citedin from pre-specified seminal anchor papers	Not executed. The anchor PMIDs are also not enumerated in the protocol: the registered OSF protocol's Supplementary Files 1 (section 7) and 3 (section 4.1) both defer the list to the manuscript Methods, where it was not reported	0
9	Grey literature: conference abstracts (ASCO, ESMO, JSMO, EHA) and guideline documents (NCCN, ASCO, ESMO, JSCO, JCOG)	No dedicated search of the meeting libraries or guideline portals was executed, and no strategy document for this source exists among the registered OSF protocol's Supplementary Files 1–4. The OpenAlex sample (source 5) was the open-index substitute for conference proceedings and was itself only partially executed. Guideline and consensus documents entered the pool only if they were retrieved through the primary PubMed search	0
10	Backward snowballing of the reference lists of included records	Not executed	0

Sources 3, 8, 9 and 10 are deviations from the registration; each is set out in the table below, and Section 2.3 of the manuscript refers here for that detail. Source 6 was attempted and failed, and source 5 was executed only in part. WHO ICTRP is not counted here: the registration states explicitly that it is not searched, on the ground that it overlaps substantially with ClinicalTrials.gov and that the review's scope prioritises published syntheses over individual trial protocols, so its absence is not a deviation.

7. PRESS 2015 documentation

Each of the four database strategies in the registered protocol carries a table documenting the strategy against the eight elements of the PRESS 2015 guideline [McGowan J, Sampson M, Salzwedel DM, et al. J Clin Epidemiol 2016, 75, 40–46]. The table recorded for the MEDLINE strategy is reproduced below; Supplementary Files 2–4 of the protocol carry the same eight elements for the Cochrane, Web of Science and CINAHL strategies, differing only where an element does not apply or where direct database access was deferred. This is the review team's own element-by-element documentation, prepared before the search was executed. It was not an independent peer review of the strategies by an information specialist or librarian, and is not presented as one; the absence of an external search peer review is stated as a limitation in Section 4.5 of the manuscript. This PRESS documentation refers to the registered June 2026 primary PubMed strategy and its database-specific translations.

PRESS 2015 element	Status recorded in the registered protocol	Documented in
Translation of the research question (PCC)	Complete	Supp File 1, section 2
Boolean and proximity operators	Complete (AND/OR; no proximity operators used)	Supp File 1, section 3
Subject headings (MeSH)	Complete (explicit [Mesh] tags)	Supp File 1, section 3
Free-text terms ([tiab])	Complete	Supp File 1, section 3
Spelling variants and truncation	Complete (neoplasm*, tumor*, tumour*, sarcoma*, component*, facilitat*, barrier*, strategies)	Supp File 1, section 3
Limits and filters	Complete (date, language, publication-type exclusion)	Supp File 1, section 4
Errors or anomalies	None detected; parse failures handled at the fetch stage	Supp File 1, section 5
Reproducibility	Complete (full query string, E-utilities calls and implementation script)	Supp File 1, sections 3 and 5

The four protocol files are archived at the root of the OSF project (DOI 10.17605/OSF.IO/ZKD53) as supp_file_1_medline_pubmed.docx, supp_file_2_cochrane.docx, supp_file_3_wos.docx and supp_file_4_cinahl.docx.

Search reproducibility

Full search files, execution timestamps and PMID lists for each of the Part 1 sources are archived in the OSF project (DOI 10.17605/OSF.IO/ZKD53): the search-strategy documents under 01_Search_strategies/, the screening logs and the raw API responses for ClinicalTrials.gov, OpenAlex and Semantic Scholar under 02_Screening_logs/, and the four pre-registered database strategies at the project root.

Part 2 — protocol amendments and revision-stage searches

Exploratory native searches were conducted on 20–21 September 2026 in CINAHL Plus, Cochrane CDSR, Cochrane CENTRAL, and Web of Science Core Collection. Database-specific adaptations of the original four conceptual search blocks were used; the Web of Science search used Title, Abstract and Author Keywords fields. The publication-date cut-off was inadvertently set to 21 May 2026, whereas the primary PubMed search covered publications through 21 June 2026. Screening of the additional records

was not undertaken. No record from these searches contributed to the final 82-record evidence set, Figure 1, or any analysis. The table below summarises the registered amendments and the separate clarification of the documented primary PubMed date limit.

Date	Recorded change	Final status
20 September 2026	Additional native database searches were recorded.	Exploratory searches; no records entered the final evidence set.
21 September 2026	A plan was recorded to screen the additional records without an AI-assisted pre-filter and to strengthen full-text assessment to independent dual review.	Screening of the additional records was not undertaken, and no record was incorporated into final eligibility assessment; independent dual full-text review of the original Stage 2 pool was retained.
22 September 2026	The decision to use the original 106-record Stage 2 pool and apply 100% independent dual full-text assessment was recorded.	Implemented for the original Stage 2 pool; the final included set was 82 records after reconciliation.
22 September 2026, registration clarification	The previously documented publication-date cut-off for the primary PubMed search was corrected.	The executed primary PubMed search covered 1 January 2010 to 21 June 2026; its 2,648-record retrieval count was unchanged.
