# Supplementary material for: Mapping Integrative Decision-Making Frameworks in Advanced Cancer Care: A Scoping Review of Diagnostic, Supportive-Care, and Therapeutic Integration
Source: Cancers (Basel). 2026 Oct 8;18(19):3245. doi: 10.3390/cancers18193245 (PMC13664350; doi:10.3390/cancers18193245)
Supplement: Supplementary file 1 [file cancers-18-03245-s001.zip › File S2_PRISMA_ScR_22item_checklist_v19.pdf]

Supplementary File S2

PRISMA-ScR: 22-item Checklist

Preferred Reporting Items for Systematic reviews and Meta-Analyses extension for Scoping Reviews (PRISMA-ScR); see main-text reference [18]

Manuscript: DST scoping review, revised version (2 October 2026) · final synthesis pool n = 82

#	Item	Reporting	Location in the revised manuscript
**TITLE**			
**1**	Title	Identify the report as a scoping review.	Title page ("Mapping Integrative Decision-Making Frameworks in Advanced Cancer Care: A Scoping Review of Diagnostic, Supportive-Care, and Therapeutic Integration")
**ABSTRACT**			
**2**	Structured summary	Provide a structured summary that includes background, objectives, eligibility criteria, sources of evidence, charting methods, results, and conclusions.	Abstract (Background/Objectives, Methods, Results, Conclusions headings; states eligibility, sources of evidence, dual screening and charting method; 269 words excluding the four section labels, 273 counted on whitespace)
**INTRODUCTION**			
**3**	Rationale	Describe the rationale for the review in the context of what is already known.	§1, paras 1–2
**4**	Objectives	State the objectives being addressed.	§1, para 4 (four numbered descriptive objectives, i–iv, plus a separately stated secondary conceptual-synthesis objective)
**METHODS**			
**5**	Protocol and registration	Indicate whether a review protocol exists; provide registration information.	Section 2.1 (OSF registration, amendment dates, protocol deviations, and the distinction between the final evidence set and exploratory revision-stage searches); Supplementary File S1, Part 2 (amendment chronology).
**6**	Eligibility criteria	Specify characteristics of the sources of evidence used as eligibility criteria (PCC).	§2.2 (Population, Concept, Context; E1–E13 exclusion codes)
**7**	Information sources	Describe all information sources in the search and date last searched.	Section 2.3 and Supplementary File S1, Part 1 (information sources used to construct the final evidence set; primary PubMed search executed on 21 June 2026). Supplementary File S1, Part 2 records the separate exploratory native searches executed on 20–21 September 2026; their records were not incorporated into the final evidence set.
**8**	Search	Present the full electronic search strategy for at least 1 database.	Section 2.4 and Supplementary File S1, Part 1 (the complete executed PubMed search strategy, including field tags, limits, filters, and retrieval count). Supplementary File S1, Part 2 summarises protocol amendments relating to the exploratory searches that were not incorporated into the final evidence set.
**9**	Selection of sources	State the process for selecting sources of evidence.	§2.5 (Stage A rule-based auto-exclusions + Stage B AI pre-filter with a combined 1,504-record exclusion-pool audit, n = 151; Stage 1 dual-reviewer title/abstract screening with K.I. adjudication of five persistent conflicts; Stage 2 = 100% independent dual full-text assessment of all 106 records)
**10**	Data charting process	Describe methods of charting including calibration, whether	§2.6 (32-field form, pilot-tested on the first five included records; AI-generated initial draft entries

#	Item	Reporting	Location in the revised manuscript
		independently by two reviewers.	verified and corrected field by field by K.M. for all 82 included records; for the two records added at final eligibility reconciliation the framework categories were assigned directly against the finalised definitions rather than through the label-consolidation route used for the original 80; Y.S. re-charted a 20% verification sample, n = 16, drawn from the original 80-record included set; K.I. adjudicated 8 discordances) + Supplementary File S5
**11**	Data items	List and define all variables for which data were sought.	§2.7 + Supplementary File S3
**12**	Critical appraisal (opt.)	If done, provide a rationale for including or excluding critical appraisal.	§2.9 (not performed; JBI methodology explicitly optional for scoping reviews)
**13**	Synthesis of results	Describe the methods of handling and summarising the data that were charted.	§2.8 (reproducible Python pipeline; strict and inclusive coverage matrices; inductive consolidation of framework descriptors recorded by the charting reviewer; two-step CFIR mapping in which keyword matches served only as retrieval cues and all final domain assignments were human contextual classifications, with independent verification of the 16-record sample and adjudication of discordant classifications) + §2.8 (unit-of-analysis stratification) + Supplementary Table S3 + §2.8 (three-component cross-axis coupling scoring of all 22 emergence-candidate records, with reviewer-agreement analysis) + Supplementary Table S2
**RESULTS**			
**14**	Selection of sources	Give numbers of sources of evidence screened, assessed for eligibility, and included; PRISMA flow diagram.	§3.1 para 1 + Figure 1
**15**	Characteristics of sources	For each source of evidence, present characteristics for which data were charted.	§3.1 para 2 + Supplementary Table S4
**16**	Critical appraisal (opt.)	If done, present data on critical appraisal.	Not applicable (per Item 12 rationale)
**17**	Results of individual sources	For each included source of evidence present the relevant data that were charted.	Supplementary Table S5 (S5a bibliographic and S5b classification panels: selected per-record charted variables, not the full 32-field chart) + Supplementary Table S2 (22 cross-axis candidate records)
**18**	Synthesis of results	Summarise and/or present the charting results as they relate to the review questions and objectives.	§3.2 (D-S-T × B/C/E coverage) + §3.3 (inductively derived framework categories) + §3.4 (CFIR) + §3.5 (cross-axis coupling); Figures 2–4; Supplementary Tables S1–S4 and S6 and Supplementary File S7
**DISCUSSION**			
**19**	Summary of evidence	Summarise the main results; consider relevance to key groups.	§4.1 (principal findings) + §4.3 (practice/policy/research)
**20**	Limitations	Discuss limitations of the scoping review process.	§4.5 (Strengths and Limitations)
**21**	Conclusions	Provide a general interpretation of the results; potential implications and next steps.	§5 (Conclusions) + §4.4 (Adaptive framework as candidate hypothesis for prospective testing) + Figure 5
**FUNDING**			
**22**	Funding	Describe sources of funding and role of funders.	Back matter, Funding section (no external funding)
