# Supplementary material for: Mapping Integrative Decision-Making Frameworks in Advanced Cancer Care: A Scoping Review of Diagnostic, Supportive-Care, and Therapeutic Integration
Source: Cancers (Basel). 2026 Oct 8;18(19):3245. doi: 10.3390/cancers18193245 (PMC13664350; doi:10.3390/cancers18193245)
Supplement: Supplementary file 1 [file cancers-18-03245-s001.zip › File S3_Charting_form_field_definitions_v22.pdf]

Supplementary File S3

Charting form and field definitions

DST Scoping Review · OSF DOI 10.17605/OSF.IO/ZKD53 · 32 fields

The charting form comprised 32 fields organised into 11 blocks (A–K).

Block	Field	Definition / value type	Purpose
**A. Bibliographic**	1. PMID	Integer (PubMed ID)	Anchor / de-duplication
**A**	2. First author	Text (LastName Initials)	Reference build
**A**	3. Year	Integer (publication year)	Reference build
**A**	4. Country / setting	Free text	Descriptive
**A**	5. Journal	Text (full journal title, as recorded in PubMed; see the note below on how it is rendered in Table S5a and File S8)	Reference build
**B. Study design + Population**	6. Study design	Dropdown as pre-specified on the form (RCT, cluster RCT, non-randomised trial, protocol, prospective cohort, retrospective cohort, systematic review, framework/model paper, mixed methods, other). Records whose design fell under "other" were reclassified post hoc, after charting, into the finer categories reported in Table S4 and Table S3 (guideline/consensus statement, Delphi consensus, cross-sectional study, prospective qualitative multimethod study); these four categories were not options on the pre-specified form.	Descriptive
**B**	7. Cancer type(s)	Free text	Descriptive
**B**	8. Treatment line	Dropdown (1st-line / 2nd-line / 3rd+ / Any / Mixed / NR)	Descriptive
**B**	9. Age range	Free text (mean/median + range)	Descriptive
**B**	10. Advanced-cancer definition (verbatim)	Free text (as written by authors)	Eligibility audit trail
**C. Setting**	11. Setting	Dropdown (Outpatient / Inpatient / Mixed / NA / NR)	Descriptive
**C**	12. Multi / Single / Population-based	Dropdown (Multicentre / Single-centre / Population / NA / NR)	Descriptive
**D. D-S-T components**	13. D (Diagnosis) narrative	Free text (action-linked description)	§3.2 axis coding
**D**	14. S (Supportive care) narrative	Free text (action-linked description)	§3.2 axis coding
**D**	15. T (Therapy) narrative	Free text (action-linked description)	§3.2 axis coding
**E. Implementation (CFIR)**	16. Facilitators (CFIR domains)	Free text	§3.4 CFIR mapping
**E**	17. Barriers (CFIR domains)	Free text	§3.4 CFIR mapping

**F. B/C/E dimension-presence flags**	18. B — Biological (OS/PFS/response)	Dropdown (● reported / — not reported)	§3.2 B/C/E dimension coding
**F**	19. C — Clinical (toxicity/QoL)	Dropdown (● / —)	§3.2 B/C/E dimension coding
**F**	20. E — Existential (PROs/dignity)	Dropdown (● / —)	§3.2 B/C/E dimension coding
**G. Authors' synthesis**	21. Authors' conclusions	Free text	§3.5 emergence reading
**G**	22. Authors' limitations	Free text	Context
**H. Typology**	23. Inductive typology — primary	Free text	§3.3 framework-category consolidation input
**H**	24. Typology — secondary	Free text (or None)	§3.3 framework-category consolidation input
**I. Integration depth**	25. Depth	Dropdown (all-three / two-of-three / one-centrally). Charted independently of the Central/Mentioned/Absent classification: a domain classified as Central contributes; a domain classified as Mentioned contributes only when it has an explicit functional linkage to another domain; contextual, descriptive, eligibility-only and outcome-only mentions do not contribute. Depth is therefore not the count of non-Absent domains.	First condition of the §3.5 emergence-candidate screen
**I**	26. All-3-dim outcome flag	Dropdown (YES / NO; the registered protocol also allowed “partial” and “single”). The charted judgement that biological, clinical and existential outcomes were all reported. This is NOT the same as the B, C and E presence flags (fields 18–20): across the 82 included records the outcome flag is positive for 16, whereas all three presence flags are set for 32.	Second condition of the §3.5 emergence-candidate screen (the first is field 25). All 16 flagged records are also all-three on depth, so the screen returns 22 records on either condition; see Supplementary File S5 for the sensitivity of the set to this field’s reliability (kappa 0.333).
**J. Tracking**	27. Charted by	Dropdown (KM / YS)	Provenance
**J**	28. Charted date	Date (YYYY-MM-DD)	Provenance
**J**	29. Notes / caveats	Free text	Provenance
**K. YS re-charting**	30. YS re-charted	Dropdown (YES / NO)	20% verification tracking
**K**	31. YS re-chart date	Date (YYYY-MM-DD)	20% verification tracking
**K**	32. Discordance alert	Free text	Charting-block kappa audit

Note on the charting procedure and the pilot. The form was pilot-tested on the first five included records on 7 August 2026, as the registered protocol specified for the first few included studies; no field was added, removed or redefined as a result. Charting then proceeded with AI assistance: an initial draft entry was generated for each record from its full text, and K.M. verified and corrected every field against the source article. Only the author-finalised values were used in the analyses. The per-record notes published in Supplementary File S8 carry the date on which each record was verified.

Note on field 5 (Journal). The field was charted as the full journal title as recorded in PubMed. Supplementary Table S5a reproduces that value verbatim, including the lower-case form and the “official journal of …” subtitle where PubMed carries one; Supplementary File S8 stores the same journal

normalised to its short title in headline case, with the subtitle removed. The two therefore differ in wording for 30 of the 82 records while identifying the same journal, and the References use the MDPI-abbreviated form again. No PMID, record or classification differs between the two files.

Note on field 25 (Integration depth). Integration depth was charted as a separate variable and was not calculated mechanically from the Central/Mentioned/Absent classification; rather, it reflected the number of D-S-T domains functionally integrated within the framework or care pathway. A domain classified as Central contributed to integration depth, whereas a domain classified as Mentioned contributed only when it had an explicit functional linkage to another domain (e.g., through assessment, triggering, referral, deliberation, or decision support). Contextual, descriptive, eligibility-only, or outcome-only mentions did not contribute to integration depth. Thus, integration depth was not calculated simply as the number of non-Absent domains. Across the 82 included records the resulting distribution was all-three 22, two-of-three 33, and one-centrally 27.

Note on the three distinct "all three" variables. (i) Integration depth = all-three (field 25): all three D-S-T domains functionally integrated; 22/82 records. (ii) All-three-dimensions outcome flag (field 26): biological, clinical and existential outcomes all reported; 16/82 records, every one of which is also all-three on depth. The §3.5 screen is the union of (i) and (ii) and therefore returns 22 records. (iii) The B, C and E presence flags (fields 18–20) all set: 32/82 records, only 17 of which are also all-three on depth. Variable (iii) is not a screening condition and must not be read as the "all three dimensions" of the screen. Depth and the presence flags are published per record in Table S5b. The outcome flag is not a column of Table S5b and cannot be re-derived from Table S4, which is aggregate only; it is published per record in Supplementary File S8.

Field-level completion by record group

Field block	Original 80-record set	2 records added at reconciliation
A Bibliographic (1–5)	charted	charted
B Study design and population (6–10)	charted	charted
C Setting (11–12)	charted	charted
D D/S/T narratives (13–15)	charted	charted; consensus synthesis coding after final reconciliation
E Implementation, CFIR (16–17)	charted	charted
F B/C/E presence (18–20)	charted	charted
G Authors' conclusions and limitations (21–22)	charted	charted
H Typology free text (23–24)	charted as free-text descriptors	direct final category assignments; no pre-consolidation free-text descriptors
I Depth and all-3-dim flag (25–26)	charted	charted
J Tracking (27–29)	charted	charted
K Y.S. re-charting (30–32)	fields 30–32 populated for the 16 records of the verification sample; not applicable for the remaining 64	not applicable: outside the 16-record verification sample. Field 30 is recorded as NO; fields 31–32 are empty

All applicable charting fields are represented for all 82 records. The two records added at final eligibility reconciliation (Conduit 2021 and Huang 2020) were charted on the same 32-field form as the original 80, including the implementation (CFIR) narrative block. For fields 23–24, no pre-consolidation free-text typology descriptors were generated; instead, the final framework categories were assigned directly against the finalised definitions. Fields 30–32 were used only for the pre-specified Y.S. re-charting verification and were populated only where applicable, so empty cells in that block are not missing data. An earlier version of this file recorded the CFIR and typology blocks as not charted for these two records;

Supplementary File S8 and the 82-record master charting workbook show that both blocks carry values, and that statement is withdrawn.

Operational definitions of the framework types

These definitions were applied when deciding whether a record described an identifiable decision structure, and which kind. They are referenced from Section 2.2 of the main text. A study design was never classified as a framework in itself; what was mapped was the decision structure described within each record.

Term	Operational definition
**Framework**	An explicit conceptual or organising structure identifying relevant domains, components, relationships, or sequential processes involved in clinical decision-making.
**Model**	A conceptual, explanatory, or predictive representation of how specified factors interact to inform or influence a clinical decision.
**Decision aid**	A structured tool supporting patients and/or clinicians in comparing options by clarifying choices, benefits and harms, values, preferences, or trade-offs.
**Intervention**	A structured clinical or supportive-care approach incorporating an identifiable decision process such as assessment, interpretation, management selection, implementation, and/or reassessment.
**Care pathway**	An organised sequence of assessments, decisions, interventions, or referrals guiding management across a defined clinical trajectory.
**Implementation strategy**	An organised method used to integrate, operationalise, or sustain a decision-making process, intervention, or care pathway in practice.

A record was mapped only where an identifiable and reproducible organisation of decision-relevant components linked assessment, interpretation or deliberation to a choice, action, implementation, reassessment, or treatment or care modification. “Reproducible” means that a second reader applying these definitions to the same text would identify the same structure; it is the criterion that keeps the mapping rule from being satisfied by an incidental mention. Study design did not by itself make a record eligible or ineligible: randomised trials, observational studies, protocols, guidelines and interventions were each mapped when they contained such a structure. Integration depth, which is a separate judgement, is defined under field 25 above and in Section 2.7 of the main text.

Operational rules for the D/S/T axis classification

Each D-S-T domain was classified per record as Central, Mentioned or Absent. Central requires that the domain is an explicit organising, decision-relevant or action-triggering component of the framework or pathway. Mentioned indicates explicit presence without a central decision or action role. Absent indicates no meaningful representation. Three negative examples were predefined and applied throughout: advanced or stage-IV eligibility alone does not make Diagnosis Central; receipt of anticancer therapy alone does not make Therapy Central; and symptoms or quality of life measured only as outcomes do not make Supportive care Central. The strict coverage matrix counts an axis only when it is Central; the inclusive matrix counts an axis when it is Central or Mentioned. These rules are the same ones referenced from Section 2.8 of the main text.

CFIR mapping procedure

CFIR mapping was a two-step procedure applied to the charted facilitator and barrier narratives. First, a predefined keyword dictionary identified candidate implementation-related text; keyword matches were never treated as final classifications. Second, K.M. reviewed each candidate in context and either assigned it to one of the five CFIR 2.0 domains or rejected it where the contextual meaning did not support an

assignment. Final domain assignments are therefore human contextual classifications rather than keyword counts. Each record contributed at most one facilitator hit and at most one barrier hit per domain. The per-record assignments are published in Supplementary File S7, where every one of the 443 record–domain–role rows carries, in its match_terms field, either the retrieval cue that brought the passage forward or the provenance of the classification — 17 rows record that the domain was named directly in the charted narrative with no keyword retrieval, and 6 that the classification was added at adjudication — together with a charted evidence excerpt. The keyword dictionary itself was not retained as a separate file and the mapping script has not survived, so the dictionary cannot be reproduced in full and is not reconstructed here. What the execution record does support is the set of terms that matched at least one record, and those terms are listed by domain in the table below. A term that was in the original list but matched no record left no trace in the output, so the recovered list is a lower bound on the dictionary and not the dictionary itself. Of the 443 rows, 420 carry one or more matched terms, 17 record that the CFIR domain was named directly in the charted narrative with no keyword retrieval, and 6 were added at adjudication and carry "adjudication-added; no original keyword hit in the K.M. mapping" instead of a term; those six are the worked examples of the domain-boundary decisions described above. Because a keyword match served only to retrieve candidate text and every final assignment was a human contextual classification, the completeness of the dictionary bears on the reproducibility of the retrieval step and not on the validity of the classifications, which were independently verified in the 16-record sample and adjudicated as reported in Supplementary File S5. The mapping was independently verified by Y.S. in the pre-existing random 16-record sample and the discordant domain–role classifications were adjudicated by K.I.; the agreement statistics and the adjudication outcome are reported in Supplementary File S5.

Keyword cues recoverable from the execution record

Terms are shown as matched, case-insensitively; several are stems (for example "implementa", "structur") because the retrieval step matched on stems. 49 distinct terms are recoverable across the five domains.

CFIR 2.0 domain	n	Terms that matched at least one record
**Innovation**	8	adaptability, cost, evidence strength, intervention, protocol, relative advantage, standardised, standardized
**Outer Setting**	4	cultur, family-centred, patient needs, reimbursement
**Inner Setting**	15	clinic, communication, culture, hospital, institution, leadership, multidisciplinary, network, organisation, readiness, resourc, staff, staffing, structur, unit
**Individuals**	10	attitud, clinician, confidence, experience, individual, knowledge, nurse, physician, skill, training
**Implementation Process**	12	early initiat, engagement, evaluat, implementa, plan, planned, planning, plans, reflect, schedul, timing, workflow
