# Supplementary material for: Mapping Integrative Decision-Making Frameworks in Advanced Cancer Care: A Scoping Review of Diagnostic, Supportive-Care, and Therapeutic Integration
Source: Cancers (Basel). 2026 Oct 8;18(19):3245. doi: 10.3390/cancers18193245 (PMC13664350; doi:10.3390/cancers18193245)
Supplement: Supplementary file 1 [file cancers-18-03245-s001.zip › File S4_AI_prefilter_specification_v8.pdf]

Supplementary File S4

AI pre-filter: pre-registered specification and implemented procedure

1. Purpose

To pre-filter the PubMed search output (n = 2,540 after rule-based Stage A exclusions) using a large language model (Claude, Anthropic) applied under a pre-specified classification prompt, prior to human dual-reviewer screening.

2. Numerical flow (corrected)

PubMed records identified: 2,648
Stage A rule-based auto-exclusions: 108 (E2/E8/E9/E11)
Stage B input: 2,648 − 108 = 2,540
Stage B AI classifications: INCLUDE 823 + MAYBE 321 + EXCLUDE 1,396 = 2,540 (verified)
AI-INCLUDE pool (INCLUDE + MAYBE): 823 + 321 = 1,144 → to Rayyan dual review
Stage B AI-EXCLUDE pool: 1,396 records
Combined exclusion pool for audit: Stage A (108) + Stage B AI-EXCLUDE (1,396) = 1,504

3. Audit design

A blinded audit sample of n = 151 (seed 20260612) was drawn from the combined 1,504-record exclusion pool. The registered rule defines the audit pool as the AI-EXCLUDE pool, that is the Stage B EXCLUDE records, which on execution numbered 1,396; the audit was applied instead to the Stage A rule-based exclusions and the Stage B EXCLUDE records together, 108 + 1,396 = 1,504. The registration states no count for that pool, and auditing the combined pool is wider than registered. K.M. and Y.S. independently re-screened each sampled record under blind conditions, blinded to the AI classification and its rationale. Acceptance criterion (pre-registered): Wilson 95% upper bound of the false-exclusion rate < 5% AND Cohen's kappa ≥ 0.61 (or PABAK ≥ 0.61 with the kappa paradox disclosed).

Observed:
Recovered records: 1/151 (FER = 0.66%)
Wilson 95% upper CI: 3.66% (< 5% threshold — PASS)
Observed agreement Po: 99.34%
PABAK: 0.987 (Almost perfect)

4. Model and prompt

Pre-registered model identifier: claude-opus-4-7 (Anthropic), to be called through the Anthropic API at temperature = 0, one record per call.
Executed model identifier: not captured. The run was carried out through an agent interface that returns no per-request model metadata and exposes no temperature parameter, so neither the exact model snapshot nor the effective decoding parameters were recorded. The pre-registered deterministic settings were specified but not verifiably applied, and the pre-filter is therefore not claimed to be exactly reproducible. See Section 5.
Prompt: reproduced in full in Section 6 below, and archived in the OSF registration component (AI pre-filter specification, Section 3). Structured output ({decision, rationale, matched_axis}).

Human oversight: AI classifications governed only the pre-filter routing step. All subsequent screening (Rayyan dual review) was performed by human reviewers with AI tools OFF and blinded mode ON.

5. Implemented procedure (verified against the execution record, September 2026)

The Stage B pre-filter was executed on 21 June 2026 on the 2,540 records that passed the Stage A rule-based auto-exclusions. Screening was carried out by an Anthropic Claude model accessed as a general-purpose subagent through an agent interface, rather than through direct Anthropic API calls as described in the pre-registered specification above. Because that interface does not return per-request model metadata, the exact model snapshot identifier was not captured in the execution log. The interface likewise does not expose a temperature parameter, so the pre-registered temperature = 0 setting was not explicitly applied; the effective decoding parameters were the agent defaults and were not recorded. Deterministic settings were therefore specified in the protocol but not verifiably applied at execution, and the run should not be described as fully deterministic or exactly reproducible.

Each record was presented to the model as PMID, title (truncated to 300 characters), journal (truncated to 100 characters), publication year, and abstract (truncated to 2,000 characters). All 2,540 records had an abstract; 747 records (29.4%) had abstracts that reached the 2,000-character cap and were therefore truncated, and 3 titles reached the 300-character cap. The model received no full text, reference lists, supplementary materials, citation information, or human screening decisions. Records were submitted in batches of 40 rather than one record per call, so records within a batch shared a single model context; this differs from the pre-registered single-record independent-evaluation design.

The system prompt instructed the model to classify each record as INCLUDE, MAYBE, or EXCLUDE against the pre-registered inclusion criterion and to return strict JSON containing decision, a one-sentence rationale, and matched_axis. No automatic API-level retry was implemented. Records were submitted in slices of 40, and the run comprised three submission passes — a 10-record test pass, a full pass over all 2,540 records, and one re-submission pass covering the records still without a usable decision. The full pass returned 2,526 decisions, of which 2,200 were usable and matched an input PMID; the re-submission pass covered 331 records and returned 332 decisions, of which 331 matched. Decisions were merged with precedence retry > full > test, and any returned identifier not present in the input PMID set was discarded. The merged log therefore draws 10 records from the test pass, 2,199 from the full pass and 331 from the re-submission pass, which is how the three passes reconcile to 2,540; note that 2,200 + 331 is not the correct decomposition, because one full-pass record is superseded by the re-submission pass and the test-pass records are additional to both.

Source: the Stage B execution audit of 23 September 2026, which quotes the merge log line “test (10) + full (2526 → 2200 unique) + retry (332 → 331 unique)” and the per-pass source attribution “test 10 / full 2,199 / retry 331” recorded in stage_b_all_decisions_2026-06-21.json.

Submission pass	Records submitted	Decisions returned	Usable and matched	Contributed to the merged log
Test	10	10	10	10
Full	2,540	2,526	2,200	2,199
Re-submission (retry)	331	332	331	331
Merged log	—	—	—	2,540

After merging, no record remained without a decision, so the pre-specified API-FALLBACK rule (API error or non-parseable JSON routed conservatively to the AI-INCLUDE pool) was implemented but never invoked: zero records were assigned API-FALLBACK and zero JSON parse errors were logged.

The final decision log contains one entry per record for all 2,540 records, each with PMID, decision, free-text rationale, matched_axis, and a source-batch label. Rationales are present for 2,540/2,540 records

(100%) and matched_axis for 1,205/2,540. Decision counts were INCLUDE 823, MAYBE 321, and EXCLUDE 1,396. INCLUDE and MAYBE records formed the 1,144-record AI-INCLUDE pool that advanced to dual-reviewer screening; EXCLUDE records were combined with the 108 Stage A rule-based exclusions to form the 1,504-record pool subjected to the Stage 0 blinded audit. Per-record model identifiers, timestamps, token counts, and raw model responses were not retained, and the original agent task output files are no longer available; the structured per-record decision log is therefore the authoritative execution record and is archived on OSF.

6. Classification prompt

The prompt is reproduced here in full so that it can be read without leaving this document. It is the prompt as pre-registered in the AI pre-filter specification lodged with the OSF registration (Section 3, Claude prompt template). {TITLE}, {JOURNAL}, {YEAR} and {ABSTRACT} are placeholders populated from the bibliographic record.

```
[SYSTEM]
You are an evidence-synthesis screening assistant for a scoping review on integrative decision-making
frameworks in adult advanced cancer pharmacotherapy. Classify a single record by title and abstract
against the pre-registered inclusion criterion.

Pre-registered inclusion criterion:
A record is INCLUDE-eligible if the full text would explicitly address at least one of the three D-S-T
components (Diagnosis, Supportive care, or Therapy) within an integrative advanced-cancer decision-
making context.

Population restrictions:
- Adults only (>=18 years).
- Advanced / metastatic / recurrent / refractory / relapsed solid or haematological cancer.
- Curative-intent or early-stage cancer-only studies are EXCLUDE.

Decision options:
- INCLUDE: abstract clearly meets the criterion.
- MAYBE:   abstract is ambiguous but plausibly meets the criterion.
- EXCLUDE: abstract clearly fails the criterion OR matches an E1-E13 exclusion code.

Output format (strict JSON, no extra text):
{
  "decision": "INCLUDE" | "MAYBE" | "EXCLUDE",
  "rationale": "<one sentence, <=40 words>",
  "matched_axis": ["D"|"S"|"T"] (if INCLUDE or MAYBE)
}

[USER]
Title: {TITLE}
Journal: {JOURNAL}
Year: {YEAR}
Abstract: {ABSTRACT}
```

This is the classification instruction as registered. In the executed run it was applied through the agent interface described in Section 5: records were submitted in slices of 40 and each record additionally carried its PMID. The decision log returned by the run conforms to the output schema shown above, carrying a decision, a free-text rationale and matched_axis for each of the 2,540 records, but the verbatim text as transmitted to the subagent was not retained in the execution record, so the prompt is reported here as registered rather than as logged.
