# Supplementary material for: Mapping Integrative Decision-Making Frameworks in Advanced Cancer Care: A Scoping Review of Diagnostic, Supportive-Care, and Therapeutic Integration
Source: Cancers (Basel). 2026 Oct 8;18(19):3245. doi: 10.3390/cancers18193245 (PMC13664350; doi:10.3390/cancers18193245)
Supplement: Supplementary file 1 [file cancers-18-03245-s001.zip › File S5_Kappa_report_v19.pdf]

Supplementary File S5

Inter-Rater Reliability Report — Screening, Charting, Cross-Axis, and CFIR Analyses

1. Screening-stage agreement (eligibility decisions)

Agreement on eligibility decisions is reported below for each screening stage. These values correspond to Figure 1 and to Sections 2.5 and 3.1 of the manuscript. They are a separate exercise from the verification and reliability analyses in Sections 2–6 of this file, which report agreement on charted variables, cross-axis coupling scores and CFIR classifications within the final set of included records; the two have different units, denominators, and reviewers' tasks and should not be pooled. For the Stage 0 audit, Cohen's kappa is 0.000 even though the two reviewers agreed on 150 of 151 records: with 150 of the 151 cells in a single category the expected agreement equals the observed agreement, so kappa collapses. This is the kappa paradox under extreme prevalence imbalance, and PABAK (0.987), which is not subject to it, is reported alongside. The 2 × 2 table is given below. In the table, Po is the observed agreement and κ is Cohen's kappa.

Screening stage	Records	Po (%)	κ	PABAK	Reviewers and outcome
Stage 0 — blinded audit of the combined exclusion pool	151	99.34	0.000	0.987	K.M. and Y.S., in a separate blinded Rayyan project. One record recovered as eligible: FER 1/151 = 0.66% (95% Wilson upper bound 3.66%, below the pre-registered 5% threshold); it was returned to screening.
Stage 1 — title/abstract dual screen	1,144	91.87	0.523	0.837	K.M. and Y.S. in Rayyan, blind mode on and AI tools off. 93 conflicts (8.1%): 88 resolved by K.M. + Y.S. consensus, 5 adjudicated by K.I. Stage 2 pool = 106.
Stage 2 — full-text eligibility assessment (100% independent dual review)	106	97.17	0.920	0.943	K.M. and Y.S. assessed all 106 records independently; 103/106 agreement before reconciliation. Three disagreements reconciled by consensus: final set 82 included and 24 excluded.

The pre-registered procedure of full-text assessment by K.M. with 20% independent verification by Y.S. was strengthened to 100% independent dual assessment in response to peer review; the Stage 2 row above reports that strengthened design. The three Stage 2 disagreements were resolved by K.M. and Y.S. in discussion without recourse to adjudication: PMID 39928067 (Slavova-Boneva et al.) remained EXCLUDE because the study population was older adults receiving systemic anticancer therapy and was not restricted to an advanced, metastatic, recurrent, or refractory population; PMID 32202060 (Conduit et al., 2021) and PMID 32019540 (Huang et al., 2020) were reclassified INCLUDE because both concern adult populations with advanced or incurable cancer and evaluate structured integrative decision-making pathways addressing at least one D-S-T component.

Stage 0 audit: K.M. × Y.S. cross-table (n = 151)

	Y.S. Include	Y.S. Exclude	Total
**K.M. Include**	0	1	1
**K.M. Exclude**	0	150	150
**Total**	0	151	151

Observed agreement 150/151 = 99.34%; expected agreement 99.34%; Cohen's κ = 0.000; PABAK = 2(0.9934) − 1 = 0.987. The single discordant record (PMID 36562276) was reconciled to INCLUDE and returned to screening, which is also the numerator of the false-exclusion rate, FER = 1/151 = 0.66% (two-

sided 95% Wilson interval 0.12% to 3.66%). Agreement and the false-exclusion rate are different quantities that coincidentally share a numerator of one. Source: stage_0_final_stats_2026-06-23.json, archived on OSF.

2. Charting verification design

Charting used an AI-generated initial draft entry for each record, which K.M. verified and corrected field by field against the source article on the pre-registered 32-field form; only the author-finalised values were used. The figures below are computed from those finalised values. Y.S. independently re-charted a deterministic 20% verification sample (n = 16) drawn from the original 80-record included set using seed 20260612, preserved from earlier audit stages. Selection was based only on record identity and order and was independent of the subsequent consolidation of the free-text framework descriptors into recurring categories, so the final framework-category assignments did not influence selection of the verification sample. The sample was therefore fixed before the two records added at revision consensus (PMID 32202060, PMID 32019540) entered the included set, and those two records were not retrospectively added to the verification sample; the re-charting statistics below accordingly retain n = 16 and their original 80-record sampling frame. The re-chart covered the bibliographic descriptors, the Diagnosis/Supportive-care/Therapy narratives, the Biological/Clinical/Existential presence flags, integration depth, and the all-three-dimensions outcome flag; Cohen’s kappa and PABAK were computed for the categorical blocks reported below. It did not extend to the derived CFIR domain mapping, which was verified separately; those results are reported in Section 6 below.

Audit sample size and stratification. The pre-registered rule was n_audit = max(ceil(0.10 × |AI-EXCLUDE pool|), 50), capped at the pool size, where the registration defines the AI-EXCLUDE pool as the Stage B EXCLUDE records and states no count for it. The audit was applied to the combined Stage A and Stage B exclusion pool, which on execution held 1,504 records (108 Stage A + 1,396 Stage B), so ceil(0.10 × 1,504) gave n = 151; this broadened the audit population beyond the registered Stage B AI-EXCLUDE set and is reported as a deviation from the registered wording. The protocol also pre-specified stratification of the sample by source (PubMed versus non-PubMed); because the exclusion pool was entirely PubMed-derived, the stratification collapsed to a single stratum and the sample was drawn as a simple random sample under the pre-registered seed. Sensitivity of the acceptance rule: the rule required the two-sided 95% Wilson upper bound on the false-exclusion rate to remain below 5%. At n = 151 that bound is 3.66% for one recovered record, 4.70% for two, and 5.68% for three, so the audit would have failed its pre-specified criterion at three or more recovered eligible records.

3. Kappa per charted field block (n = 16 re-charted records)

Block	Po (%)	Cohen's κ	PABAK	Landis–Koch interpretation
**B (Biological, •/—)**	93.75	0.871	0.875	Almost perfect
**C (Clinical, •/—)**	93.75	0.636	0.875	Substantial (κ depressed by prevalence imbalance)
**E (Existential, •/—)**	100.00	1.000	1.000	Perfect
**Depth (3-category)**	93.75	0.904	0.906	Almost perfect (multi-category PABAK)
**All-3-dim flag (normalised)**	68.75	0.333	0.375	Fair — reflects field-guide ambiguity for that supplementary flag

4. Charting-discordance adjudication (8 discordances across the 16 re-charted records)

Per the pre-specified protocol, all discordances between K.M. and Y.S. were adjudicated by K.I. (senior reviewer, oncology–palliative-care expert) blind to K.M.'s original chart. K.I.'s adjudicated value became the final chart value for each field. The 8 adjudications:

#	PMID	Field / Block	K.M. / Y.S.	K.I. adjudicated (final)
1	26646758 (Strasser 2016)	B — Biological	K.M. ● / Y.S. —	● (K.M.)
2	20597705 (Gaertner 2010)	C — Clinical	K.M. ● / Y.S. —	● (K.M.)
3	26768772 (Grudzen 2016)	Depth	K.M. one-centrally / Y.S. two-of-three	one-centrally (K.M.)
4	38726807 (Klastersky 2024)	All-3-dim flag	K.M. YES / Y.S. partial→NO	YES (K.M.)
5	37231417 (Deis 2023)	All-3-dim flag	K.M. NO / Y.S. YES	NO (K.M.)
6	34750145 (Potenza 2021)	All-3-dim flag	K.M. NO / Y.S. YES	NO (K.M.)
7	28859646 (Oostendorp 2017)	All-3-dim flag	K.M. NO / Y.S. YES	NO (K.M.)
8	27821313 (Maltoni 2016)	All-3-dim flag	K.M. NO / Y.S. YES	NO (K.M.)

K.I. reviewed the D/S/T narratives and B/C/E flags for each discordant record and provided a final adjudicated value. In this cohort of 8 discordances, K.I.'s adjudicated values aligned with K.M.'s original chart in all 8 cases. The adjudication record does not document a reason for that pattern, and none is inferred here.

Sensitivity of the emergence-candidate set. The Section 3.5 screen applies two conditions: integration depth of all-three, or a positive all-three-dimensions outcome flag. In the master chart the flag is positive for 16 records and every one of them is also all-three on depth, so the screen returns the same 22 records as the depth condition alone. Discordances in either field can therefore move a record into or out of the set, and both were examined. Depth: there was one discordance, PMID 26768772 (Grudzen 2016), K.M. one-centrally versus Y.S. two-of-three; neither value is all-three, so the set is unchanged. Outcome flag: there were five discordances. Adopting Y.S.'s value for all five would leave PMID 38726807 (Klastersky 2024) and PMID 27821313 (Maltoni 2016) in the set, since both are all-three on depth irrespective of the flag, and would add PMID 37231417 (Deis 2023), PMID 34750145 (Potenza 2021) and PMID 28859646 (Oostendorp 2017), which are two-of-three on depth and were flagged positive by Y.S. only. The candidate set would then be 25 records rather than 22. The three tied highest-scoring records (Kaasa 2018, Smith CB 2017, Temel 2011) are all-three on depth and are unaffected under either reviewer's values.

D-S-T axis-classification reliability and strict/inclusive sensitivity analysis

These statistics were reported in Section 3.2 of earlier versions of the manuscript and are reproduced here unchanged. They cover the 80 records independently included by both reviewers; the two records added at final eligibility reconciliation carry consensus codes and are not part of this comparison, and this exercise is separate from the 16-record full-field re-charting described under the charting blocks above.

Three-category agreement (Central / Mentioned / Absent) was 62.50% for Diagnosis (Cohen's $\kappa = 0.429$), 88.75% for Supportive care ($\kappa = 0.653$), and 72.50% for Therapy ($\kappa = 0.524$). The lower agreement for Diagnosis predominantly reflected distinctions between Central and Mentioned rather than frequent disagreement between complete absence and any presence.

When Central and Mentioned were collapsed as presence for the inclusive sensitivity analysis, agreement increased to 86.25% (κ = 0.726) for Diagnosis, 97.50% (κ = 0.886) for Supportive care, and 87.50% (κ = 0.716) for Therapy.

Sensitivity analysis showed strong concordance between the strict (Central only) and inclusive (Central or Mentioned) cell rankings (Spearman's ρ = 0.895). Inclusive coding increased individual cell counts by 6–23 records, and 7 of the 9 cells remained within one average-rank position. Diagnosis-related combinations remained relatively under-represented under both definitions.

5. Cross-axis coupling scoring reliability (n = 22 emergence-candidate records)

K.M. and Y.S. scored the 22 emergence-candidate records with the same three-component rubric, each component from 0 to 2 (Section 2.8; the operational criteria and the per-record scores are in Supplementary Table S2). Exact agreement was 21/22 (95.5%) for explicit D-S-T linkage, 19/22 (86.4%) for triggering or reassessment, and 19/22 (86.4%) for longitudinality, with linear-weighted Cohen's κ of 0.909, 0.723, and 0.697 respectively. Total scores were identical for 17 of the 22 records (77.3%) and were strongly rank-correlated (Spearman's ρ = 0.916). No component was scored 0 for any record, so the observed scale spans two categories and the linear weighting is arithmetically equivalent to unweighted κ for these data. Kaasa 2018, Smith CB 2017 and Temel 2011 were the only records receiving 6/6 from both reviewers. The rubric was developed during peer-review revision and is not externally validated.

6. CFIR mapping verification and adjudication (n = 16 records, 160 domain–role classifications)

Y.S. independently classified facilitator and barrier presence or absence across the five CFIR 2.0 domains in the pre-existing random 16-record charting-verification sample, without access to K.M.'s corresponding classifications. This generated 160 binary record–domain–role classifications. K.M. and Y.S. agreed on 116 of 160 (72.5%; Cohen's κ = 0.453; PABAK = 0.450) before reconciliation: K.M. rated 76 cells Present and Y.S. 88, with 16 cells Present for K.M. only and 28 Present for Y.S. only. The 44 discordant classifications were adjudicated by K.I. Six K.M.-Absent / Y.S.-Present classifications were retained as Present in the final mapping and no K.M.-Present classification was removed, so the final CFIR dataset contains 443 record–domain–role instances across the 82 included records (241 facilitators, 202 barriers). This adjudication is a separate exercise from the charting-field adjudication in Section 4: it concerns CFIR domain–role cells, not the D/S/T narratives or the B/C/E flags, and the two should not be pooled.

Block	n	Observed agreement	Cohen's κ	PABAK
**Overall**	**160**	**72.5%**	**0.453**	**0.450**
**Facilitator classifications**	80	75.0%	0.497	0.500
**Barrier classifications**	80	70.0%	0.398	0.400
**Innovation**	32	78.1%	0.440	0.563
**Outer Setting**	32	75.0%	0.152	0.500
**Inner Setting**	32	71.9%	0.410	0.438
**Individuals**	32	62.5%	0.270	0.250
**Implementation Process**	32	75.0%	0.455	0.500

The low κ for Outer Setting arose despite 75.0% observed agreement and reflects marked prevalence imbalance in that domain, which is why PABAK is shown alongside κ throughout. Values are the pre-reconciliation K.M. versus Y.S. comparison; they are not recomputed from the adjudicated values, which would no longer describe inter-rater agreement.
