# Supplementary material for: Mapping Integrative Decision-Making Frameworks in Advanced Cancer Care: A Scoping Review of Diagnostic, Supportive-Care, and Therapeutic Integration
Source: Cancers (Basel). 2026 Oct 8;18(19):3245. doi: 10.3390/cancers18193245 (PMC13664350; doi:10.3390/cancers18193245)
Supplement: Supplementary file 1 [file cancers-18-03245-s001.zip › Table_S1_Inductively_derived_framework_categories_v6.pdf]

Supplementary Table S1

Inductively derived framework categories with definitions and example PMIDs

DST Scoping Review · OSF DOI 10.17605/OSF.IO/ZKD53 · n = 82 records · 160 raw labels + 4 direct author assignments = 164 category assignments

Framework category	Definition	n records	n assignments	Example PMIDs
**Early integrated palliative care**	Time- or diagnosis-based embedding of specialist palliative care alongside oncology from the point of advanced-cancer diagnosis (Temel/Kaasa/Maltoni lineage). Includes simultaneous/proactive/co-located outpatient or inpatient PC services, day-hospital models, whole-trajectory pathways, organisational integration frameworks with standardised care pathways and multidisciplinary teams, and benchmarking/audit frameworks evaluating the structure of oncology–PC integration.	30	34	32031887, 30344075, 27821313, 21555700
**Prognosis-informed shared decision-making**	Structured decision architectures — decision aids, values-clarification tools, prognostic-risk communication scripts, and MDT decision meetings — that integrate prognostic information, patient values, and treatment-utility trade-offs into choices among anticancer treatment options (continuation, dose de-escalation, switching, clinical-trial participation, or transition to BSC/palliative pathways).	27	44	29310605, 21483008, 21542415
**Trigger-based palliative-care integration**	Criterion-driven referral to specialist PC using automated or protocolised triggers — needs-screening thresholds, prognostic algorithms, symptom/ePRO alarms, ED admission, treatment milestones, or behavioural-default/opt-out designs. The referral rule, rather than the calendar interval, is the framework's core mechanism.	16	17	26768772, 28306372, 39982729
**Geriatric-assessment-driven oncology**	Comprehensive geriatric assessment (CGA/GA/PGA) as the gating and tailoring instrument for treatment intensity, supportive interventions, toxicity mitigation, and communication in older adults with advanced cancer. Includes Delphi/consensus care-planning bundles for older adults, web/EHR-embedded GA workflows, telehealth GA, and impairment- or vulnerability-triggered treatment-allocation frameworks that operate on GA data.	13	22	31697365, 26884557, 34741815
**Advance care planning and goals-of-care communication**	Structured programs whose primary framework is ACP, goals-of-care discussion, advance-directive integration, ethical treatment-limitation guidance, or EOL-preference elicitation, producing documented plans that guide subsequent treatment-limitation, hospice-transition, or EOL de-intensification decisions.	14	17	34922770, 30913904, 29907553
**Multidisciplinary supportive care intervention**	Interdisciplinary supportive-care programs distinct from specialist-PC integration and from GA/decision frameworks — stepped/tiered psychosocial care, screening-triggered psycho-oncology, needs-based nurse-led triage, nutrition + psychology–focused supportive care, CBT-based stigma-reduction, prehabilitation, and supportive-care-informed	9	12	32937942, 38631891, 39556485

Framework category	Definition	n records	n assignments	Example PMIDs
	treatment-tolerability pathways.			
**Digital / ePRO symptom monitoring and decision support**	Frameworks in which digital tools drive symptom management, treatment adjustment, or referral decisions — ePRO with severity alerts, wearable/passive sensing, AI-based remote monitoring, telehealth PC delivery, EHR-embedded clinical decision support, and automated default nudges.	7	11	41915775, 30652585, 26646758
**Prognostic staging / risk-stratification framework**	Conceptual or clinical frameworks that stratify patients by overall physiological reserve and prognostic risk — stratified supportive-care concepts, traffic-light treatment and supportive-care categories, cachexia staging, and prognostic risk tiers — and prescribe supportive-care intensity and treatment posture according to the stratum.	3	5	38726807, 39088825, 22831160
**Other / uncategorized**	Framework types represented too sparsely for a dedicated framework category — currently biomarker/NGS-guided precision-oncology decision pathways (ESCAT/MTB-supported treatment matching).	1	2	40122129

Note: framework categories are inductively derived and are not mutually exclusive; a record may carry up to two framework descriptors, and the two descriptors may map to the same or to different categories. Therefore, the record counts do not sum to 82 and the Section 3.3 percentages do not sum to 100%. "n records" is the number of included records in which the category was represented; "n assignments" is the number of category assignments mapped to it, counting the 160 raw labels and the 4 direct author assignments together. The residual Other category captured 2/164 (1.2%) category assignments, both of them raw labels, for a single record with a niche framework. The full raw-label-to-category mapping for all 164 category assignments is provided in Supplementary File S6.
Two records entered the included set at revision consensus following 100% independent dual full-text assessment: PMID 32202060 (Conduit et al., 2021) — advance care planning and goals-of-care communication, and trigger-based palliative-care integration; and PMID 32019540 (Huang et al., 2020) — advance care planning and goals-of-care communication, and trigger-based palliative-care integration. No free-text descriptor had been charted for either record, so these four category assignments were made directly by K.M. against the definitions in this table and are flagged as such in Supplementary File S6. The category definitions themselves were not changed, and the 160 raw labels from the original 80-record set were not re-mapped.
