# Supplementary material for: Mapping Integrative Decision-Making Frameworks in Advanced Cancer Care: A Scoping Review of Diagnostic, Supportive-Care, and Therapeutic Integration
Source: Cancers (Basel). 2026 Oct 8;18(19):3245. doi: 10.3390/cancers18193245 (PMC13664350; doi:10.3390/cancers18193245)
Supplement: Supplementary file 1 [file cancers-18-03245-s001.zip › Table_S2_Cross_axis_coupling_evidence_v10.pdf]

# Supplementary Table S2

## Cross-axis coupling analysis of the 22 emergence-candidate records

*How these 22 records were selected. The emergence-candidate screen applied either of two charted conditions: integration depth of all-three (charting field 25), meaning all three D-S-T domains functionally integrated within the framework or care pathway; or a positive all-three-dimensions outcome flag (field 26), the charted judgement that biological, clinical and existential outcomes were all reported. Both fields are in the registered charting form, but the screen combining them was defined at the synthesis stage and was not itself pre-registered. Depth was all-three for 22 of the 82 included records and the outcome flag was positive for 16; all 16 are among the 22, so the union is the 22 records listed below. The depth condition is reproducible from the Depth column of Table S5b. The outcome flag must not be confused with the B, C and E presence flags, which are set for 32 records and were not a screening condition. Meeting the screen identifies a record for closer reading; it does not by itself establish cross-axis coupling, which is judged below.*

**Scoring rubric.** The 22 records meeting the emergence-candidate screen were evaluated with a three-component rubric developed during peer-review revision. Each component was scored 0, 1 or 2 against the operational criteria below, giving a total of 0–6 per record. K.M. and Y.S. scored all 22 records using the same rubric.

**Explicit D-S-T linkage.** 0, the D, S and T domains merely co-occur, with no stated influence between domains and no decision-level connection. 1, an explicit link exists between at least two domains, or integration across D-S-T is described in general terms, but an operational pathway connecting all three is not specified. 2, all three domains are explicitly connected, so that information or action in one domain feeds a judgement or action in another.

**Triggering or reassessment.** 0, no trigger, threshold, event, time point or reassessment rule. 1, reassessment or triggering is present as a concept, but what changes what is left general. 2, an explicit trigger — symptoms, performance status, prognosis, a laboratory or imaging finding, patient preference, treatment response or a scheduled time point — initiates reassessment, referral or treatment change in another domain.

**Longitudinality.** 0, a single static assessment or decision, with no repeat evaluation. 1, follow-up or repeated assessment is described, but no systematic feedback or adaptation procedure. 2, repeated assessment at multiple time points, continuous monitoring, and adaptation over the treatment course are built into the framework or pathway.

**Agreement.** Agreement across the three ordinal components was evaluated with linear-weighted Cohen's κ and the concordance of total scores with Spearman's rank correlation: κ = 0.909 for explicit D-S-T linkage, 0.723 for triggering or reassessment and 0.697 for longitudinality; total scores were identical for 17 of the 22 records (77.3%) with ρ = 0.916. No component was scored 0 for any record, so the observed scale spans two categories and the linear weighting is arithmetically equivalent to unweighted κ for these data.

**Highest-scoring records.** Kaasa 2018, Smith CB 2017 and Temel 2011 were the only records to receive the maximum total score of 6/6 from both reviewers. Scores of 3 to 5 are common across the remaining records, so cross-axis coupling is reported as a graded pattern rather than a present/absent property.

**Table S2a. Component and total cross-axis coupling scores for all 22 emergence-candidate records. link, explicit D-S-T linkage; trig, triggering or reassessment; long, longitudinality. Each component is scored 0–2 and the total 0–6.**

| PMID | Author, Year | K.M. link | K.M. trig | K.M. long | K.M. total | Y.S. link | Y.S. trig | Y.S. long | Y.S. total | 6/6 from both |
|---|---|---|---|---|---|---|---|---|---|---|
| **42211526** | Li S 2026 | 1 | 1 | 1 | 3 | 1 | 1 | 1 | 3 | No |
| **41379446** | Huang HL 2025 | 1 | 1 | 1 | 3 | 1 | 1 | 1 | 3 | No |
| **38193250** | Hjermstad MJ 2024 | 1 | 1 | 1 | 3 | 1 | 1 | 1 | 3 | No |
| **39088825** | Phillips I 2024 | 2 | 2 | 1 | 5 | 2 | 2 | 1 | 5 | No |
| **38726807** | Klastersky J 2024 | 2 | 1 | 1 | 4 | 2 | 1 | 1 | 4 | No |
| **39690895** | Laryionava K 2024 | 2 | 1 | 1 | 4 | 1 | 2 | 1 | 4 | No |
| **37154941** | Poisson C 2023 | 2 | 1 | 1 | 4 | 2 | 1 | 1 | 4 | No |

| | | | | | | | | | | |
|---|---|---|---|---|---|---|---|---|---|---|
| **35597176** | Ribi K 2022 | 1 | 1 | 1 | 3 | 1 | 2 | 1 | 4 | No |
| **33432524** | Hui D 2021 | 2 | 1 | 2 | 5 | 2 | 1 | 2 | 5 | No |
| **31867978** | Verhoef MJ 2020 | 1 | 1 | 1 | 3 | 1 | 1 | 1 | 3 | No |
| **32031887** | Temel JS 2020 | 1 | 1 | 1 | 3 | 1 | 1 | 1 | 3 | No |
| **32826262** | Nelson A 2024 | 2 | 1 | 1 | 4 | 2 | 1 | 1 | 4 | No |
| **30157701** | Corsi DC 2019 | 2 | 2 | 1 | 5 | 2 | 2 | 1 | 5 | No |
| **30677754** | Villalobos M 2019 | 2 | 2 | 2 | 6 | 2 | 2 | 1 | 5 | No |
| **30344075** | Kaasa S 2018 | 2 | 2 | 2 | **6** | 2 | 2 | 2 | **6** | **Yes** |
| **28306372** | Adelson K 2017 | 1 | 2 | 1 | 4 | 1 | 2 | 1 | 4 | No |
| **28029308** | Temel JS 2017 | 1 | 1 | 1 | 3 | 1 | 1 | 2 | 4 | No |
| **28561696** | Smith CB 2017 | 2 | 2 | 2 | **6** | 2 | 2 | 2 | **6** | **Yes** |
| **27821313** | Maltoni M 2016 | 1 | 2 | 2 | 5 | 1 | 1 | 2 | 4 | No |
| **24654196** | Colombet I 2012 | 1 | 1 | 2 | 4 | 1 | 1 | 2 | 4 | No |
| **21542415** | Smith TJ 2011 | 2 | 2 | 2 | 6 | 2 | 2 | 1 | 5 | No |
| **21555700** | Temel JS 2011 | 2 | 2 | 2 | **6** | 2 | 2 | 2 | **6** | **Yes** |

**Table S2b. Charted D/S/T evidence and score-based outcome for the same 22 records. Entries under D / S / T evidence are extracts from the charting workbook, not quotations from the source articles.**

| # | PMID | First author, Year | D evidence (summary) | S evidence (summary) | T evidence (summary) | Score-based outcome |
|---|---|---|---|---|---|---|
| **1** | 42211526 | Li S 2026 | Explicit within the EIPC communication/ACP component: ongoing discussions addressed disease understanding, prognosis, and treatment goals, with future-care preferences documented. No formal prognostic score or biomarker-guided reassessment was used. | Central: structured EIPC began within 4 weeks of advanced-cancer diagnosis and used a multidisciplinary team for comprehensive assessment, symptom-targeted management, psychosocial support, nutrition/rehabilitation, practical support, and regular follow-up. | Explicit: personalized care plans and ACP were discussed with patients and families to align treatment with goals, values, and preferences, including future and end-of-life decisions; EIPC was delivered as simultaneous care alongside anticancer therapy and was associated with less chemotherapy in the last 6 weeks of life. | K.M. 3/6, Y.S. 3/6 — candidate record. |
| **2** | 41379446 | Huang HL 2025 | Action-triggering entry condition: a new diagnosis of stage IV oral cavity cancer initiated the SOP pathway. The model did not use biomarker reassessment or a formal prognostic score, and prognosis disclosure was not a standardized component. | Central: multidisciplinary palliative care was offered alongside anticancer treatment through palliative-care specialists, social workers, psychologists/chaplains, and physiatrists, with symptom and psychosocial needs addressed through the SOP pathway. | Central: the structured 3-talk SDM process (choice, option, decision talk) explicitly compared anticancer and palliative-care options, discussed risks and benefits, elicited patient preferences, and aligned decisions with individual values and end-of-life preferences. | K.M. 3/6, Y.S. 3/6 — candidate record. |
| **3** | 38193250 | Hjermstad MJ 2024 | Explicit: the physician education programme included communication of prognosis and breaking bad news, and the pathway incorporated an estimated life expectancy ≤12 months plus tumour- and patient-centred information to support decisions; no biomarker reassessment or formal multivariable prognostic score was used. | Central: all intervention patients received mandatory specialised palliative-care referral, repeated ESAS/Eir symptom assessment before consultations, systematic discussion/documentation of symptoms, and coordinated oncology–palliative care throughout the anticipated last line of treatment. | Central: performance status, weight loss and patient preferences were explicitly documented and used to decide initiation or cessation of anticancer therapy, with shared decision-making between oncology and palliative-care teams; end-of-life treatment use was the primary outcome. | K.M. 3/6, Y.S. 3/6 — candidate record. |
| **4** | 39088825 | Phillips I 2024 | Central: the proposed traffic-light model 'stages the host' using prognostic and fitness information including ECOG performance status, tumour stage, comorbidities, symptom/nutritional screening, functional testing, systemic inflammatory markers (e.g., mGPS/SIPS), and cachexia to classify | Central: comprehensive supportive care is explicitly stratified by traffic-light group, ranging from universal/self-directed measures for fitter patients to targeted or specialist interventions, symptom control, rehabilitation, nutrition, psychological support, palliative care, and | Central: traffic-light status is explicitly linked to suitability for anticancer therapy—green patients are very likely to receive treatment, red patients are very unlikely despite additional intervention, and orange patients have uncertain fitness that supportive interventions may improve; | K.M. 5/6, Y.S. 5/6 — candidate record. |

| | | | | | | |
|---|---|---|---|---|---|---|
| | | | patients as green, orange, or red. | advanced-care planning for greater need. | goals of care should be negotiated with every patient. | |
| **5** | 38726807 | Klastersky J 2024 | Central: the model stratifies patients primarily by prognosis and response to therapy into curable, incurable, end-of-life, and cured/stabilized states; prognosis communication and advance care planning are emphasized particularly for incurable and end-of-life disease. | Central: supportive care is tailored across all stages, combining medical symptom management, psychosocial support, early palliative care, advance care planning, terminal care, bereavement, survivorship support, and integrative medicine according to patient needs and disease trajectory. | Central: prognosis/response strata are linked to different therapeutic priorities—potentially curative treatment for curable disease, palliative oncology for incurable disease, minimization of futile interventions in terminal care, and surveillance/maintenance after cure or stabilization—with interdisciplinary input. | K.M. 4/6, Y.S. 4/6 — candidate record. |
| **6** | 39690895 | Laryionava K 2024 | Central: the two-part decision aid explicitly incorporates prognosis. Part II is completed at disease progression/staging and includes individualized prognostic information such as median survival and the proportion expected to benefit from further treatment; prognosis and expected benefit are clarified by junior and senior physicians before the patient discussion. | Explicit and decision-linked: best supportive care and palliative care are presented as concrete alternatives to further anticancer treatment. The implementation model also requires palliative-care education, timely interdisciplinary networking, and improved knowledge of supportive/palliative options, although it does not deliver a separate symptom-management programme. | Central: the customizable decision aid supports shared decisions about continuing versus forgoing further anticancer therapy. Part I elicits life goals, values and preferences; Part II compares the benefits and burdens of the next cancer-specific treatment with forgoing treatment and focusing on best supportive care. | K.M. 4/6, Y.S. 4/6 — candidate record. |
| **7** | 37154941 | Poisson C 2023 | Explicit but not formalized as a prognostic score: CSMs integrate the patient's clinical status and oncological prognosis with anticipated acute-event prognosis when defining goals and intensity of care. A pre-existing Decision-Aid Form (DAF) classifies planned care intensity into four levels (ICU, conditional ICU, non-invasive ward care, or exclusive palliative care); these strata were strongly associated with subsequent survival, although the DAF itself is a care-intensity tool rather than a validated prognostic model. | Central: supportive-care optimization was a formal CSM domain and included pain and refractory-symptom management, psychological/psychiatric care, difficult patient-family-team relationships, social measures, sedation discussions, discharge planning, and palliative-care-team involvement. | Central: CSMs directly addressed whether to continue, reduce, change, or stop anticancer treatment and whether to limit invasive care/ICU escalation. Among cases with an oncology-treatment discussion, discontinuation was validated in 44 cases and exclusive palliative care was frequently chosen; collegial recommendations were documented in the EHR. | K.M. 4/6, Y.S. 4/6 — candidate record. |
| **8** | 35597176 | Ribi K 2022 | Explicit through prognostic communication/understanding, but not through a formal prognostic model. The framework asks clinicians to assess curability/incurability, educate patients about the expected disease course without chemotherapy and best-/worst-case prognosis, and verify patient/family understanding. The authors explicitly note that the decision process is not based on prognostication alone. | Central and integrated into the decision process: the framework addresses multidimensional symptoms, coping, family burden, existential/end-of-life preparation, assurance of concurrent palliative/supportive care with chemotherapy, multidisciplinary involvement, and continuous palliative care when chemotherapy is not pursued. | Central: the framework is specifically designed for decisions for or against systemic anticancer treatment with palliative intent. It structures discussion of chemotherapy goals, expected benefits and side effects, monitoring of treatment effect, the option to stop chemotherapy at any time, risk-benefit weighing, a deliberate pause, and the final patient/shared/clinician decision. | K.M. 3/6, Y.S. 4/6 — candidate record. |
| **9** | 33432524 | Hui D 2021 | Central: the proposed prognostic continuum begins with prognostic prediction/formulation and progresses through prognostic disclosure, prognostic awareness and | Explicit and integrated: specialist palliative care is positioned across the prognostic continuum, with communication support, coping strategies/counselling, | Central: the final step of the continuum is prognosis-based decision-making, translating prognostic information into personalized and goal-concordant decisions. Downstream | K.M. 5/6, Y.S. 5/6 — candidate record. |

| | | | | | | |
|---|---|---|---|---|---|---|
| | | | prognostic acceptance. Accurate clinical/model-based prognostication is intended to reduce uncertainty and errors and to provide the foundation for subsequent personalized communication and decisions. | psychology referral and follow-up/logistical support tailored to the patient's cognitive and emotional state. These interventions aim to reduce distress, enhance acceptance and support care planning. | decisions explicitly include cancer treatment use/continuation, advance care planning, DNR orders and intensity/quality of end-of-life care. | |
| 10 | 31867978 | Verhoef MJ 2020 | Proposed (not empirically implemented as an intervention): the integrated-care model in Figure 1 of the source article uses poor prognosis, high risk of severe treatment toxicity, uncertain treatment response, and an unpredictable disease trajectory as cues for integrated care. The curative track includes informing patients about possible future scenarios of the disease trajectory, with continuous evaluation of goals of care. No formal prognostic score or survival-prediction tool was tested. | Central proposed track: early concurrent palliative/supportive care alongside disease-directed care, based on four-dimensional physical, psychological, social, and spiritual care. It includes symptom management/supportive care, continuous assessment of patients' values/wishes/priorities, family involvement, advance care planning, and multidisciplinary communication. In the empirical cohort, PCCT referral and proactive symptom-management plans were also measured. | Explicit in the proposed model: shared decision-making about treatment and needed care, including treatment choices, hospital admissions, life-sustaining treatments, goals/preferences for future medical treatment and care, and advance care planning. The empirical cohort measured LST discussions/limitations but did not test a treatment-decision intervention. | K.M. 3/6, Y.S. 3/6 — candidate record. |
| 11 | 32031887 | Temel JS 2020 | Active within the intervention manual: palliative-care clinicians were instructed to assess and enhance prognostic awareness and illness understanding in patients and caregivers across the illness trajectory. Prognostic understanding and communication were also measured with the Prognosis and Treatment Perceptions Questionnaire. No biomarker reassessment or formal prognostic score was used. | Central: early integrated palliative care was delivered by a PC physician or advanced practice nurse within 4 weeks of enrollment and at least monthly until death, alongside oncology care. The manual explicitly included symptom assessment and treatment, support for patient/caregiver coping, therapeutic relationship, and EOL-care planning; hospitalized intervention patients were also required to be seen by the PC team. | Active but secondary to the supportive-care axis: the intervention manual explicitly included assisting with treatment decision making and planning for EOL care, so treatment choices were part of the protocolized PC content rather than only a downstream outcome. No regimen-specific decision algorithm, formal decision aid, or mandated dose-reduction/cessation rule was described. | K.M. 3/6, Y.S. 3/6 — candidate record. |
| 12 | 32826262 | Nelson A 2024 | Proposed/action-linked D axis. Prognosis and patients' prognostic understanding are explicitly connected to treatment-option discussions: the authors state that prognosis was the link for oncologists to discuss available options, and Figure 4 of the source article proposes that updates on each patient's understanding of—and willingness to discuss—prognosis should be available when treatment options are discussed. This was an identified gap and proposed pathway component, not a tested prognostic intervention or formal prognostic score. | Proposed S axis within the pathway. Figure 4 of the source article states that supportive-care options should be considered as a clinical pathway; the proposed practice also requires assessment/recording of symptoms and quality of life and feedback about the patient's care/support network. In the observed pathway, palliative-care consultants were not always present and supportive care was insufficiently framed as a positive alternative. | Central T axis. The study follows palliative SACT decision-making from MDT recommendation through the oncology consultation and documents changes between recommended and actual treatment (including best supportive care/no treatment). The proposed pathway calls for early presentation of all treatment options and for MDT/treatment decisions to be based on patient priorities and preferences; prompt questions such as "What if I do nothing?" are suggested to support deliberation. | K.M. 4/6, Y.S. 4/6 — candidate record. |
| 13 | 30157701 | Corsi DC 2019 | Active at the consensus-framework level. Final recommendations call for clinical prognostic | Central. The document recommends early/simultaneous palliative care alongside oncology | Active at the consensus-framework level. Antineoplastic treatment is explicitly delivered | K.M. 5/6, Y.S. 5/6 — candidate record. |

| | | | | | | |
|---|---|---|---|---|---|---|
| | | | indicators, especially the surprise question ("Would I be surprised if this patient dies in the next 6-12 months?"), within a broader prognostic assessment to identify patients for early/specialist palliative care. Prognosis/life expectancy is then used to stratify care models (>12 months vs <12 months). PPI, PaP/PaP-D and PiPS are discussed as validated prognostic tools, but are not mandated as required components. | care, with earlier assessment and treatment of symptoms, multidimensional assessment of physical/psychosocial/spiritual needs, specialist PC referral, multidisciplinary/shared care, a case manager, and systematic symptom/QoL assessment (e.g., ESAS/EAPC basic dataset). Referral can be triggered by severe symptoms, delirium, neurologic complications, emotional distress, existential/spiritual crisis, social frailty or caregiver limitations. | simultaneously with palliative care, and healthcare decisions are recommended to use progressive shared decision-making across initiation, continuation and withdrawal of palliative antineoplastic therapy. Treatment effectiveness, appropriateness/proportionality, QoL-limiting adverse effects and the patient's will are to be reassessed before decisions. Progressive disease despite second-line systemic therapy is also a PC-referral trigger; no drug-specific regimen or dose algorithm is prescribed. | |
| 14 | 30677754 | Villalobos M 2019 | Central at the proposed-framework level. Prognostic awareness is explicitly assessed and revisited across the disease trajectory, beginning with disclosure of diagnosis/prognosis and continuing at remission/stable disease, progression, and end-of-life transitions. The approach uses stepwise prognostic communication to improve illness understanding and link prognosis to realistic treatment and care decisions; no formal prognostic score is used. | Central at the proposed-framework level. Early palliative care is integrated throughout the trajectory, with continuous assessment of symptoms and needs, nurse-navigator follow-up, coping support, care coordination, and advance care planning. Palliative care is presented as an active, equal care option rather than a late downstream referral. | Central at the proposed-framework level. Milestone conversations explicitly address treatment options at diagnosis, continuation versus discontinuation during response/stable disease, re-evaluation of further cancer treatment after progression, and stopping disease-modifying treatment while transitioning to symptom-focused care. Decisions are preference-sensitive and repeatedly revisited. | K.M. 6/6, Y.S. 5/6 — candidate record. |
| 15 | 30344075 | Kaasa S 2018 ★ | Active: prognostic assessment and communication are explicitly integrated. The Commission reviews formal survival tools (PaP/D-PaP, PPS, PPI, mGPS, Feliu nomogram, PiPS), recommends objective prognostic tools to complement clinician estimates, and recommends early prognostic communication. Prognosis is linked to ACP/place and ceilings of care, palliative-chemotherapy decisions, and palliative/hospice referral criteria. | Central: early integrated palliative/supportive care across the cancer trajectory; systematic symptom and patient-reported outcome assessment with results used in decision-making; needs-based and time-based palliative-care referral criteria; psychosocial/spiritual assessment and management; primary/secondary/tertiary palliative care, care coordination, community/hospice referral, and family support. | Active and decision-linked: SDM explicitly addresses starting, changing, continuing, or stopping tumour-directed treatment and includes the option of no tumour-directed treatment. The Commission also recommends criteria to guide discontinuation (e.g., progression, performance status, nutritional status/weight loss, symptom burden), repeated treatment-plan reassessment, and embedding SDM/ACP in standardised care pathways; palliative-care participation in multidisciplinary teams is recommended. | K.M. 6/6, Y.S. 6/6 — maximum total score from both reviewers; one of the three tied highest-scoring records presented in Section 3.5. |
| 16 | 28306372 | Adelson K 2017 | Active but secondary: the trigger set was explicitly designed to identify patients for whom PC involvement could improve "prognostic clarification and/or symptom control." The standardized PC consultation included goals-of-care/advance-care-planning work and communication of bad news, linking illness/prognostic understanding to subsequent care planning. | Central: patients meeting any standardized trigger received automatic multidisciplinary PC consultation. The standardized intervention included ESAS-based symptom assessment and treatment, goals-of-care and advance care planning, transition planning, daily follow-up of new/ongoing symptoms, and coordination with families/social work for | Active: the PC team conducted or assisted discussions about new or changing goals of care and "made associated treatment adjustments." This directly links the intervention to treatment continuation/de-escalation decisions, although no regimen-selection algorithm or formal decision aid was used. The observed reduction in chemotherapy after | K.M. 4/6, Y.S. 4/6 — candidate record. |

| | | | | | | |
|---|---|---|---|---|---|---|
| | | | No formal prognostic score or survival model was used. | goal-concordant transitions. | discharge is a downstream outcome and is not the sole basis for coding T. | |
| 17 | 28029308 | Temel JS 2017 | Active (secondary): PC clinicians were trained to help patients understand prognosis and treatment goals and facilitate informed decisions. Intervention visits explicitly addressed illness understanding (38.4%). Prognostic understanding/communication was measured longitudinally with the Prognosis and Treatment Perceptions Questionnaire. This is prognostic-awareness/communication rather than a formal prognostic score. | Central: early integrated palliative care began within 4 weeks of enrollment and continued at least monthly until death, with telephone substitution when needed and inpatient PC follow-up during admissions. Intervention visits addressed symptoms (74.7%), coping (70.2%), rapport (44.4%), illness understanding (38.4%), advance-care planning (14.2%), treatment decisions (16.3%), and disposition (2.1%). | Active (secondary): PC clinicians explicitly facilitated informed decisions about care; treatment decisions were documented as a focus in 16.3% of intervention visits, alongside prognosis/treatment-goal communication and advance-care planning. There was no protocolized regimen-selection or de-escalation algorithm and routine oncology care continued, so T represents decision support rather than mandated therapy modification. | K.M. 3/6, Y.S. 4/6 — candidate record. |
| 18 | 28561696 | Smith CB 2017 ★ | Active: TEAM explicitly operationalizes prognostic awareness and communication. Education includes structured, recurrent prognosis discussions; progressive scans and worsening clinical status are cues to revisit prognosis and advance-care planning, and the conclusion recommends using a worse scan or change in ECOG performance status to discuss prognosis and realistic options. No formal prognostic score or biomarker reassessment is used. | Central: TEAM operationalizes early concurrent palliative care through dedicated time, formal symptom/psychosocial/spiritual assessment, and interdisciplinary management. The ASCO guidance summarized in the article recommends dedicated palliative care early (generally within 8 weeks), concurrent with active oncology care, with symptom/distress/function management, coping support, care coordination, and indicated referrals. | Active: TEAM includes repeated discussion of medically appropriate treatment options and their outcomes, followed by goal setting and advance-care planning; prognosis/scan changes are used to revisit realistic options. The ASCO palliative-care components summarized in the article explicitly include clarification of treatment goals and assistance with medical decision making. This is decision support rather than a regimen-selection algorithm. | K.M. 6/6, Y.S. 6/6 — maximum total score from both reviewers; one of the three tied highest-scoring records presented in Section 3.5. |
| 19 | 27821313 | Maltoni M 2016 | Active D element under this review definition. The systematic EPC intervention explicitly incorporates scheduled, checklist-based discussions covering health-related domains, designed to foster prognostic awareness and facilitate early shared decision-making among the patient, family, and oncologist. Although the cultural background in Italian clinical practice presents barriers due to limited patient prognostic awareness, the operationalized framework actively targets and guides these decision-making discussions rather than merely observing them. | Central. In the systematic EPC arm, a palliative-care specialist saw patients within 2 weeks of enrolment and every 2-4 weeks thereafter until death, using a predetermined checklist and interventions planned according to general palliative-care guidelines. The comparator received standard cancer care plus on-demand PC requested by the patient, family, or attending oncologist. EPC was intended to manage psychophysical symptoms during chemotherapy and support longitudinal palliative care/hospice use. | Active T element under this review definition. The systematic EPC intervention includes an operational component aimed at guiding treatment decisions and limiting aggressive end-of-life (EoL) care. While late-stage chemotherapy use was analyzed as a key outcome, the significantly lower rate of chemotherapy within the last 30 days of life in the intervention group (18.7% vs 27.8%) reflects the active role of EPC in facilitating treatment discontinuation and intensity adjustment through structured, early decision-making processes. | K.M. 5/6, Y.S. 4/6 — candidate record. The reviewers differ on triggering: K.M. read metastatic disease and first-line chemotherapy as an explicit trigger for activating systematic early palliative care, in contrast to symptom-triggered on-demand referral, whereas Y.S. judged the scheduled consultations and supportive-care |

| | | | | | | |
|---|---|---|---|---|---|---|
| | | | | | | intervention explicit but the rule by which change in one D-S-T domain triggers reassessment of another less clearly specified. Both scores are reported as given and were not reconciled. |
| **20** | 24654196 | Colombet I 2012 | Active, secondary prognostic axis. Entry to the weekly onco-palliative meeting (OPM) was triggered by referent-physician estimation of life expectancy <6 months for an incurable patient needing goals-of-care discussion. Within the OPM, expected survival benefit was explicitly weighed, and the PC team also addressed illness and prognostic understanding. Thus prognosis was operationalized as a trigger/input to subsequent care decisions, although no validated prognostic score was used. | Central. The integrated-care model used a weekly multidisciplinary OPM plus longitudinal joint follow-up by oncology and the specialist PC team. PC activities included symptom management, end-of-life decisions, coping, illness/prognostic understanding, hospice referral, home-care coordination, and outpatient follow-up; goals of care were revisited at subsequent OPMs. | Central and explicitly decision-linked. OPM discussions considered expected survival benefit, quality of life, aggressiveness of care, and patient preferences, with options including continuing chemotherapy (with or without PC), phase I/II trial referral, or PC only. Decisions were then submitted to the patient's wishes and goals were updated longitudinally. | K.M. 4/6, Y.S. 4/6 — candidate record. |
| **21** | 21542415 | Smith TJ 2011 | Central. The decision aid explicitly discloses prognosis and prognosis-related treatment information: incurability/chance of cure, expected survival with and without chemotherapy, response probability, and duration of disease control. Patients first indicated how much prognostic information they wanted, then received disease- and line-specific quantitative estimates and were reassessed for understanding. This is an active prognostic-disclosure/prognostic-awareness intervention, not merely baseline staging. | Active, secondary. The decision aid explicitly prompts end-of-life/supportive planning, including hospice involvement, advance directives/living wills, CPR preferences, life review, spiritual and financial issues, and preferred place of care/death. A psychologist and chaplain were available if distress occurred. These supportive-care/ACP elements are embedded in the intervention, although the study did not test a structured referral or symptom-management pathway. | Central. The intervention is a formal decision aid modelled on the Ottawa Decision Support Framework. It presents line-specific palliative-chemotherapy benefits and harms and, in the example aid, explicitly compares chemotherapy with best supportive care/hospice. The purpose is to support informed treatment choice by balancing survival/response/symptom benefit against toxicity and alternatives; actual treatment changes were not measured in this pilot. | K.M. 6/6, Y.S. 5/6 — candidate record. |
| **22** | 21555700 | Temel JS 2011 ★ | Central: early palliative care explicitly addressed illness understanding and education, including disease prognosis, to support informed decisions. Prognostic perceptions (curability) were assessed longitudinally; early PC increased the proportion who remained or became accurate about incurability (82.5% vs 59.6%; P=.02). This is active prognostic- | Central: early integrated palliative care began within 3 weeks of enrollment and continued at least monthly until death. The protocol addressed symptom management, coping with life-threatening illness for patients/families, and development of care plans/referrals, alongside standard oncology care. | Active: treatment decision making was an explicit component of the palliative-care visit guidelines. PC clinicians educated patients about prognosis and the potential benefits and burdens of medical therapies to enable informed treatment decisions. Lower IV chemotherapy use within 60 days of death among prognostically accurate early-PC patients (9.4% vs 50%; | K.M. 6/6, Y.S. 6/6 — maximum total score from both reviewers; one of the three tied highest-scoring records presented in Section 3.5. |

| | | | | | | |
|---|---|---|---|---|---|---|
| | | | awareness work, not merely baseline staging. | | P=.02) is a downstream association; T is counted because treatment decision making itself was operationalized in the intervention. | |

*Note. Records marked ★ received the maximum total score of 6/6 from both reviewers and are the three records presented in main-text Results Section 3.5. Column entries under D / S / T evidence are the charted narratives from the master charting workbook, reproduced in full; they are reviewer-generated descriptions and are not quotations from the source articles.*
