# Supplementary material for: Mapping Integrative Decision-Making Frameworks in Advanced Cancer Care: A Scoping Review of Diagnostic, Supportive-Care, and Therapeutic Integration
Source: Cancers (Basel). 2026 Oct 8;18(19):3245. doi: 10.3390/cancers18193245 (PMC13664350; doi:10.3390/cancers18193245)
Supplement: Supplementary file 1 [file cancers-18-03245-s001.zip › Table_S3_Unit_of_analysis_stratification_v8.pdf]

Supplementary Table S3

Unit-of-analysis stratification of the 82 included studies by study design and primary mapped architecture

Study design/evidence type and primary mapped architecture are two separate dimensions. Each record has exactly one study-design stratum and exactly one primary mapped architecture, so both distributions sum to 82 and to 100%. These assignments are mutually exclusive and should not be confused with the inductively derived framework categories (Table S5, Table S1), which are overlapping: a record may carry more than one framework category. Secondary architectural features coexisting within a record remain captured in the framework-category charting. Percentages are of n = 82.

Table S3a. Study design / evidence type

Study-design stratum	n	%
**Randomised trials (RCT + cluster RCT)**	24	29.3
**Non-randomised interventional studies**	11	13.4
**Protocols**	13	15.9
**Observational / qualitative / mixed-methods studies**	20	24.4
**Framework/model / Delphi papers**	4	4.9
**Guideline / consensus statements**	2	2.4
**Systematic reviews**	3	3.7
**Other**	5	6.1
**Total**	**82**	**100.0**

Table S3b. Primary mapped architecture

Primary mapped architecture	n	%
**Conceptual framework / model**	14	17.1
**Decision aid / decision-support tool**	13	15.9
**Structured intervention / care pathway / implementation strategy**	55	67.1
**Total**	**82**	**100.0**

Table S3c. Cross-tabulation of study design by primary mapped architecture

Study design	Framework/model	Decision aid/tool	Intervention/pathway/strategy	Total
**Randomised trials**	0	4	20	24
**Non-randomised interventional**	0	2	9	11
**Protocols**	0	4	9	13
**Observational /**	3	1	16	20

**qualitative / mixed-methods**				
**Framework/model / Delphi**	4	0	0	4
**Guideline / consensus**	2	0	0	2
**Systematic reviews**	1	1	1	3
**Other**	4	1	0	5
**Total**	**14**	**13**	**55**	**82**

Cell counts are record counts. Row totals equal the study-design strata in Table S3a and column totals equal the primary mapped architectures in Table S3b.
Provenance and reproducibility. The study-design stratum and the primary mapped architecture are single-valued per-record variables; the architecture was recorded alongside the 32 charted fields rather than being one of them, and both are published per record in Supplementary File S8, so every count in Tables S3a–S3c is recomputable from that dataset. An earlier version of this file stated that the per-record architecture values were archived with the charting workbook deposited on OSF; that deposit covers the original 80 records and carries no architecture column, so the values are published here instead.
