# Supplementary material for: Mapping Integrative Decision-Making Frameworks in Advanced Cancer Care: A Scoping Review of Diagnostic, Supportive-Care, and Therapeutic Integration
Source: Cancers (Basel). 2026 Oct 8;18(19):3245. doi: 10.3390/cancers18193245 (PMC13664350; doi:10.3390/cancers18193245)
Supplement: Supplementary file 1 [file cancers-18-03245-s001.zip › Table_S4_Descriptive_characteristics_v11_n82.pdf]

Supplementary Table S4

Descriptive characteristics of the included studies (n = 82)

Rounding: half-up to 1 decimal place; percentages of n = 82. Every block is a single assignment per record and totals 82, except the B, C and E rows, which count records in which that B/C/E dimension was flagged as present (fields 18–20) and are not mutually exclusive; they are not the all-three-dimensions outcome flag, which is the separate field 26.

Provenance of each block. Every count in this table is recomputable row by row from the accompanying dataset (Supplementary File S8), which publishes all 32 charted fields for all 82 records, including the two added at final eligibility reconciliation. The reconciliation decisions behind the charted values are recorded in the master charting workbook.

Category	n	%
**Study design**		
RCT	17	20.7
Protocol	13	15.9
Non-randomised trial	11	13.4
Retrospective cohort	9	11.0
Cluster RCT	7	8.5
Prospective cohort	7	8.5
Other	5	6.1
Systematic review	3	3.7
Framework/model paper	3	3.7
Mixed methods	2	2.4
Guideline/consensus statement	2	2.4
Delphi consensus	1	1.2
Prospective qualitative multimethod study	1	1.2
Cross-sectional	1	1.2
**Setting**		
Outpatient	44	53.7
Mixed	27	32.9
Inpatient	7	8.5
NR	3	3.7
Not applicable	1	1.2
**Multi/Single/Pop**		
Multicentre	38	46.3
Single-centre	37	45.1
Not applicable	6	7.3
NR	1	1.2
**Treatment line**		
NR	40	48.8
Mixed	19	23.2
1st-line	17	20.7
Any	4	4.9
2nd-line	2	2.4
**Depth**		
two-of-three	33	40.2
one-centrally	27	32.9
all-three	22	26.8
**All-3-dim flag**		
NO	66	80.5
YES	16	19.5
**B (positive)**		
positive	48	58.5

**C (positive)**		
positive	75	91.5
**E (positive)**		
positive	57	69.5
