# Supplementary material for: Mapping Integrative Decision-Making Frameworks in Advanced Cancer Care: A Scoping Review of Diagnostic, Supportive-Care, and Therapeutic Integration
Source: Cancers (Basel). 2026 Oct 8;18(19):3245. doi: 10.3390/cancers18193245 (PMC13664350; doi:10.3390/cancers18193245)
Supplement: Supplementary file 1 [file cancers-18-03245-s001.zip › Table_S5_Full_record_level_table_v11_n82.pdf]

Supplementary Table S5

Full record-level table for the 82 included studies with D/S/T axis classification and B/C/E dimension-presence flags

D/S/T axis: central (C) / mentioned (M) / absent (A). B/C/E: ● = present, — = absent (K.M. chart). Depth was classified according to the charting rules in Supplementary File S3. Framework categories are inductively derived and not mutually exclusive: a record may appear under more than one category, so category counts do not sum to the number of records. Category assignments, including four direct author assignments for the two records added at final eligibility reconciliation, are listed in Supplementary File S6. Records 39 (Conduit C 2021), 45 (Huang HL 2020) were added at final eligibility reconciliation; their D/S/T values are consensus synthesis codes and were not part of the independent dual D/S/T agreement analysis (n = 80).

The record-level table is presented as two panels linked by PMID: Table S5a carries the identifiers and bibliographic fields, Table S5b the charted classifications. Presenting all fourteen fields in one table forced every long cell to break a word at a time. The same 82 rows are also supplied as a machine-readable dataset, Supplementary File S8, which adds the study-design, setting, centre-status, treatment-line, cancer-type and all-three-dimensions-flag fields from the master charting form so that Table S4 can be recomputed per record. The accompanying dataset provides all 32 charted fields for all 82 records, together with the primary mapped architecture used in Table S3. For records outside the 16-record verification sample, fields 31–32 are empty; field 30 is blank for the 64 non-sampled records from the original 80-record set and is recorded as NO for the two records added at final eligibility reconciliation.

Table S5a. Record identifiers and bibliographic fields (n = 82)

#	PMID	Author, Year	Title	Journal	DOI
1	41932293	Aleixo G 2026	Practical Geriatric Assessment (PGA) Implementation Strategies and Correlative Evaluations (PACE-70): Protocol for a hybrid implementation-effectiveness study in three community oncology practices in the United States.	Journal of geriatric oncology	10.1016/j.jgo.2026.102965
2	41512250	Aleixo G 2026	Embedding Geriatric Oncology in a Community Practice of an Academic Health System: Implementation, Outcomes, and End-of-Life Impact.	JCO oncology practice	10.1200/OP-25-00772
3	41778625	Hao S 2026	Precision palliative care in advanced/recurrent esophageal cancer patients treated with anti-PD-1/PD-L1 immunotherapy (ESO-Shanghai 25): a randomized, phase III Trial.	Future oncology (London, England)	10.1080/14796694.2026.2637738
4	41880032	Kessler ER 2026	Use of a Delphi process to formulate a pragmatic framework for care planning in older adults with advanced bladder cancer.	Supportive care in cancer : official journal of the Multinational Association of Supportive Care in Cancer	10.1007/s00520-026-10532-1
5	42211526	Li S 2026	The impact of early integrated palliative care on symptom burden and quality of life in patients with advanced breast cancer.	Frontiers in oncology	10.3389/fonc.2026.1817750
6	40499588	Bergerot CD 2025	Telehealth Geriatric Assessment and Supportive Care Intervention (GAIN-S) Program: A Randomized Clinical Trial.	Journal of the National Comprehensive Cancer Network : JNCCN	10.6004/jnccn.2025.7020
7	41109944	Hillen B 2025	Digital and AI-assisted multimodal supportive care, combining physical activity, nutrition, and pain management during chemotherapy for advanced pancreatic cancer patients: study protocol of the European multicenter randomized controlled trial of the RELEVIUM project.	BMC cancer	10.1186/s12885-025-14867-6
8	41379446	Huang HL 2025	Shared Decision-Making With Otolaryngologists and Palliative Care Specialists in Oral Cavity Cancer.	JAMA network open	10.1001/jamanetworkopen.2025.48557
9	40122129	Kazmi F 2025	Next-generation sequencing for guiding matched targeted therapies in people	The Cochrane database of systematic	10.1002/14651858.CD014872.pub2

#	PMID	Author, Year	Title	Journal	DOI
			with relapsed or metastatic cancer.	reviews	
10	39566832	Norman KE 2025	Impact of a Criteria-Based Inpatient Palliative Oncology Consultation Model on End-of-Life Outcomes.	Journal of pain and symptom management	10.1016/j.jpainsymman.2024.11.011
11	39982729	Parikh RB 2025	Algorithm-Based Palliative Care in Patients With Cancer: A Cluster Randomized Clinical Trial.	JAMA network open	10.1001/jamanetworkopen.2024.58576
12	40812801	Richard HA 2025	Blended palliative and survivorship care intervention (POISE) for patients with metastatic oncogene-driven non-small cell lung cancer: study protocol for a pilot randomised controlled trial.	BMJ open	10.1136/bmjopen-2024-098075
13	38521641	Alibhai SMH 2024	TOward a comPrehensive supportive Care intervention for Older men with metastatic Prostate cancer (TOPCOP3): A pilot randomized controlled trial and process evaluation.	Journal of geriatric oncology	10.1016/j.jgo.2024.101750
14	41915775	Basch E 2024	Comparing the Effectiveness of Electronic Symptom Monitoring versus Usual Care in Improving Survival among Patients with Metastatic Cancer—The PRO-TECT Trial	PCORI Final Research Reports (Patient-Centered Outcomes Research Institute)	10.25302/01.2024.ihs.151133392
15	38326125	Daviu Cobian C 2024	Impact of FRAilty screening and Geriatric assessment and INtervention in older patients with epithelial Ovarian Cancer: A multicenter randomized clinical trial protocol (FRAGINOC).	Journal of geriatric oncology	10.1016/j.jgo.2024.101713
16	39340169	Ermers DJ 2024	The focus on life-prolonging anticancer treatment hampers shared decision-making in people with advanced cancer: A qualitative embedded multiple-case study.	Palliative medicine	10.1177/02692163241281145
17	39259563	Greer JA 2024	Telehealth vs In-Person Early Palliative Care for Patients With Advanced Lung Cancer: A Multisite Randomized Clinical Trial.	JAMA	10.1001/jama.2024.13964
18	38193250	Hjermstad MJ 2024	PALLiative care in ONcology (PALLiON): A cluster-randomised trial investigating the effect of palliative care on the use of anticancer treatment at the end of life.	Palliative medicine	10.1177/02692163231222391
19	39115845	Kang E 2024	Early Integrated Palliative Care in Patients With Advanced Cancer: A Randomized Clinical Trial.	JAMA network open	10.1001/jamanetworkopen.2024.26304
20	38726807	Klastersky J 2024	A new comprehensive and stratified concept for supportive care in cancer patients.	Current opinion in oncology	10.1097/CCO.0000000000001039
21	39690895	Laryionava K 2024	Feasibility of implementing a decision aid for advanced cancer patients in a comprehensive cancer centers' outpatient setting: Valuable lessons learned.	Cancer medicine	10.1002/cam4.70127
22	36822846	Marret L 2024	Implementation of palliative care day hospital for outpatients with advanced cancer.	BMJ supportive & palliative care	10.1136/spcare-2023-004206
23	39556485	Matsumoto Y 2024	Nurse-Led Screening-Triggered Early Specialized Palliative Care Program for Patients With Advanced Lung Cancer: A Multicenter Randomized Controlled Trial.	Cancer medicine	10.1002/cam4.70325
24	32826262	Nelson A 2024	Chemotherapy decision-making in advanced lung cancer: a prospective qualitative study.	BMJ supportive & palliative care	10.1136/bmjspcare-2020-002395
25	39088825	Phillips I 2024	A traffic light approach for treatment and supportive care stratification in lung cancer.	Current opinion in supportive and palliative care	10.1097/SPC.0000000000000716
26	38631891	Phillips I 2024	Early prehabilitation reduces admissions and time in hospital in patients with newly diagnosed lung cancer.	BMJ supportive & palliative care	10.1136/spcare-2024-004869
27	34750145	Potenza L 2024	Early palliative/supportive care in acute myeloid leukaemia allows low aggression end-of-life interventions: observational outpatient study.	BMJ supportive & palliative care	10.1136/bmjspcare-2021-002898
28	38824442	Temel JS 2024	Stepped Palliative Care for Patients With Advanced Lung Cancer: A Randomized Clinical Trial.	JAMA	10.1001/jama.2024.10398
29	36574052	Chvetzoff G 2023	Impact of early palliative care on additional line of chemotherapy in metastatic breast cancer patients: results from the randomized study OSS.	Supportive care in cancer : official journal of the Multinational Association of Supportive Care in Cancer	10.1007/s00520-022-07561-x
30	36356279	Culakova E 2023	Effects of a Geriatric Assessment Intervention on Patient-Reported Symptomatic Toxicity in Older Adults With Advanced Cancer.	Journal of clinical oncology : official journal of the American Society of Clinical Oncology	10.1200/JCO.22.00738
31	37231417	Deis N 2023	HELP - Heidelberg decision aid for lung cancer patients: a randomized	Trials	10.1186/s13063-023-07365-2

#	PMID	Author, Year	Title	Journal	DOI
			controlled clinical trial.		
32	37200607	Nishijima TF 2023	Survival in Older Japanese Adults With Advanced Cancer Before and After Implementation of a Geriatric Oncology Service.	JCO oncology practice	10.1200/OP.22.00842
33	37154941	Poisson C 2023	Contribution of collegial support meetings (CSM) in the management of complex situations of patients with advanced cancer.	Supportive care in cancer : official journal of the Multinational Association of Supportive Care in Cancer	10.1007/s00520-023-07782-8
34	34922770	Davidson BA 2022	Promoting timely goals of care conversations between gynecologic cancer patients at high-risk of death and their providers.	Gynecologic oncology	10.1016/j.ygyno.2021.12.009
35	35079813	Matsumoto Y 2022	Early specialized palliative care for patients with metastatic lung cancer receiving chemotherapy: a feasibility study of a nurse-led screening-triggered programme.	Japanese journal of clinical oncology	10.1093/jjco/hyab204
36	36167377	Matsuoka A 2022	Geriatric assessment and management with question prompt list using a web-based application for elderly patients with cancer (MAPLE) to communicate ageing-related concerns: J-SUPPORT 2101 study protocol for a multicentre, parallel group, randomised controlled trial.	BMJ open	10.1136/bmjopen-2022-063445
37	35597176	Ribi K 2022	Towards a novel approach guiding the decision-making process for anticancer treatment in patients with advanced cancer: framework for systemic anticancer treatment with palliative intent.	ESMO open	10.1016/j.esmoop.2022.100496
38	36071303	van der Padt-Pruijsten A 2022	The effect of a palliative care pathway on medical interventions at the end of life: a pre-post-implementation study.	Supportive care in cancer : official journal of the Multinational Association of Supportive Care in Cancer	10.1007/s00520-022-07352-4
39	32202060	Conduit C 2021	Implementing 'Goals of Care' discussion and palliative care referral for patients with advanced lung cancer: an outpatient-based pilot project.	Internal medicine journal	10.1111/imj.14817
40	33983395	Hall PS 2021	Efficacy of Reduced-Intensity Chemotherapy With Oxaliplatin and Capecitabine on Quality of Life and Cancer Control Among Older and Frail Patients With Advanced Gastroesophageal Cancer: The GO2 Phase 3 Randomized Clinical Trial.	JAMA oncology	10.1001/jamaoncol.2021.0848
41	33432524	Hui D 2021	The Importance of Prognostication: Impact of Prognostic Predictions, Disclosures, Awareness, and Acceptance on Patient Outcomes.	Current treatment options in oncology	10.1007/s11864-020-00810-3
42	34591080	Li D 2021	Geriatric Assessment-Driven Intervention (GAIN) on Chemotherapy-Related Toxic Effects in Older Adults With Cancer: A Randomized Clinical Trial.	JAMA oncology	10.1001/jamaoncol.2021.4158
43	33417481	Lu Z 2021	Early Interdisciplinary Supportive Care in Patients With Previously Untreated Metastatic Esophagogastric Cancer: A Phase III Randomized Controlled Trial.	Journal of clinical oncology : official journal of the American Society of Clinical Oncology	10.1200/JCO.20.01254
44	34741815	Mohile SG 2021	Evaluation of geriatric assessment and management on the toxic effects of cancer treatment (GAP70+): a cluster-randomised study.	Lancet (London, England)	10.1016/S0140-6736(21)01789-X
45	32019540	Huang HL 2020	Shared decision making with oncologists and palliative care specialists effectively increases the documentation of the preferences for do not resuscitate and artificial nutrition and hydration in patients with advanced cancer: a model testing study.	BMC palliative care	10.1186/s12904-020-0521-7
46	32937942	Lynch FA 2020	Feasibility and Acceptability of Fear-Less: A Stepped-Care Program to Manage Fear of Cancer Recurrence in People with Metastatic Melanoma.	Journal of clinical medicine	10.3390/jcm9092969
47	31697365	Mohile SG 2020	Communication With Older Patients With Cancer Using Geriatric Assessment: A Cluster-Randomized Clinical Trial From the National Cancer Institute Community Oncology Research Program.	JAMA oncology	10.1001/jamaoncol.2019.4728
48	32031887	Temel JS 2020	Multisite, Randomized Trial of Early Integrated Palliative and Oncology Care in Patients with Advanced Lung and Gastrointestinal Cancer: Alliance A221303.	Journal of palliative medicine	10.1089/jpm.2019.0377
49	31867978	Verhoef MJ 2020	End-of-Life Trajectories of Patients With Hematological Malignancies and Patients With Advanced Solid Tumors Visiting the Emergency Department: The Need for a Proactive Integrated Care Approach.	The American journal of hospice & palliative care	10.1177/1049909119896533
50	30157701	Corsi DC 2019	Consensus document of the Italian Association of Medical Oncology and the Italian Society of Palliative Care on early palliative care.	Tumori	10.1177/0300891618792478
51	30913904	Park EJ 2019	Feasibility of Early Application of an Advance Directive at the Time of First-Line Palliative Chemotherapy in Patients With Incurable Cancer: A Prospective	The American journal of hospice & palliative care	10.1177/1049909119839355

#	PMID	Author, Year	Title	Journal	DOI
			Study.		
52	30168410	Tapp D 2019	Evaluation of decision support tools for patients with advanced cancer: A systematic review of literature.	Palliative & supportive care	10.1017/S1478951518000512
53	31783851	van Lent LGG 2019	Realizing better doctor-patient dialogue about choices in palliative care and early phase clinical trial participation: towards an online value clarification tool (OnVaCT).	BMC palliative care	10.1186/s12904-019-0486-6
54	30677754	Villalobos M 2019	Communication along Milestones in Lung Cancer Patients with Advanced Disease.	Oncology research and treatment	10.1159/000496407
55	28734786	Engelhardt EG 2018	Clinical Usefulness of Tools to Support Decision-making for Palliative Treatment of Metastatic Colorectal Cancer: A Systematic Review.	Clinical colorectal cancer	10.1016/j.clcc.2017.06.007
56	29310605	Henselmans I 2018	A randomized controlled trial of a skills training for oncologists and a communication aid for patients to stimulate shared decision making about palliative systemic treatment (CHOICE): study protocol.	BMC cancer	10.1186/s12885-017-3838-8
57	30344075	Kaasa S 2018	Integration of oncology and palliative care: a Lancet Oncology Commission.	The Lancet. Oncology	10.1016/S1470-2045(18)30415-7
58	29907553	Laryionava K 2018	Development and Evaluation of an Ethical Guideline for Decisions to Limit Life-Prolonging Treatment in Advanced Cancer: Protocol for a Monocentric Mixed-Method Interventional Study.	JMIR research protocols	10.2196/resprot.9698
59	30652585	Wright AA 2018	The HOPE Pilot Study: Harnessing Patient-Reported Outcomes and Biometric Data to Enhance Cancer Care.	JCO clinical cancer informatics	10.1200/CCI.17.00149
60	28306372	Adelson K 2017	Standardized Criteria for Palliative Care Consultation on a Solid Tumor Oncology Service Reduces Downstream Health Care Use.	Journal of oncology practice	10.1200/JOP.2016.016808
61	28562197	Einstein DJ 2017	Improving End-of-Life Care: Palliative Care Embedded in an Oncology Clinic Specializing in Targeted and Immune-Based Therapies.	Journal of oncology practice	10.1200/JOP.2016.020396
62	28761725	Martoni AA 2017	Impact of intervention aimed at improving the integration of oncology units and local palliative care services: results of the multicentre prospective sequential MIRTO study.	ESMO open	10.1136/esmoopen-2016-000116
63	28859646	Oostendorp LJM 2017	Decision aids for second-line palliative chemotherapy: a randomised phase II multicentre trial.	BMC medical informatics and decision making	10.1186/s12911-017-0529-y
64	28561696	Smith CB 2017	Using the New ASCO Clinical Practice Guideline for Palliative Care Concurrent With Oncology Care Using the TEAM Approach.	American Society of Clinical Oncology educational book. American Society of Clinical Oncology. Annual Meeting	10.1200/EDBK_175474
65	28029308	Temel JS 2017	Effects of Early Integrated Palliative Care in Patients With Lung and GI Cancer: A Randomized Clinical Trial.	Journal of clinical oncology : official journal of the American Society of Clinical Oncology	10.1200/JCO.2016.70.5046
66	28470369	Vayne-Bossert P 2017	Integration of oncology and palliative care: setting a benchmark.	Supportive care in cancer : official journal of the Multinational Association of Supportive Care in Cancer	10.1007/s00520-017-3736-2
67	26884557	Corre R 2016	Use of a Comprehensive Geriatric Assessment for the Management of Elderly Patients With Advanced Non-Small-Cell Lung Cancer: The Phase III Randomized ESOGIA-GFPC-GECP 08-02 Study.	Journal of clinical oncology : official journal of the American Society of Clinical Oncology	10.1200/JCO.2015.63.5839
68	26768772	Grudzen CR 2016	Emergency Department-Initiated Palliative Care in Advanced Cancer: A Randomized Clinical Trial.	JAMA oncology	10.1001/jamaoncol.2015.5252
69	27821313	Maltoni M 2016	Systematic versus on-demand early palliative care: A randomised clinical trial assessing quality of care and treatment aggressiveness near the end of life.	European journal of cancer (Oxford, England : 1990)	10.1016/j.ejca.2016.10.004
70	26646758	Strasser F 2016	The effect of real-time electronic monitoring of patient-reported symptoms and clinical syndromes in outpatient workflow of medical oncologists: E-MOSAIC, a multicenter cluster-randomized phase III study (SAKK 95/06).	Annals of oncology : official journal of the European Society for Medical Oncology	10.1093/annonc/mdv576
71	26559912	Clarke G 2015	Withdrawal of anticancer therapy in advanced disease: a systematic literature review.	BMC cancer	10.1186/s12885-015-1862-0
72	25872950	do Carmo TM 2015	A phase II study in advanced cancer patients to evaluate the early transition to palliative care (the PREPArE trial): protocol study for a randomized controlled trial.	Trials	10.1186/s13063-015-0655-8
73	25173586	Rugno FC 2014	Early integration of palliative care facilitates the discontinuation of anticancer	Gynecologic oncology	10.1016/j.ygyno.2014.08.030

#	PMID	Author, Year	Title	Journal	DOI
			treatment in women with advanced breast or gynecologic cancers.		
74	24654196	Colombet I 2012	Effect of integrated palliative care on the quality of end-of-life care: retrospective analysis of 521 cancer patients.	BMJ supportive & palliative care	10.1136/bmjspcare-2011-000157
75	22831160	Vigano A 2012	The cachexia clinic: from staging to managing nutritional and functional problems in advanced cancer patients.	Critical reviews in oncogenesis	10.1615/critrevoncog.v17.i3.70
76	21779232	Gaertner J 2011	Facilitating Early Integration of Palliative Care into Breast Cancer Therapy. Promoting Disease-Specific Guidelines.	Breast care (Basel, Switzerland)	10.1159/000329007
77	21483008	Leighl NB 2011	Supporting treatment decision making in advanced cancer: a randomized trial of a decision aid for patients with advanced colorectal cancer considering chemotherapy.	Journal of clinical oncology : official journal of the American Society of Clinical Oncology	10.1200/JCO.2010.32.0754
78	22158486	Magarotto R 2011	Reduced use of chemotherapy at the end of life in an integrated-care model of oncology and palliative care.	Tumori	10.1177/030089161109700506
79	21320319	Oostendorp LJ 2011	Assessing the information desire of patients with advanced cancer by providing information with a decision aid, which is evaluated in a randomized trial: a study protocol.	BMC medical informatics and decision making	10.1186/1472-6947-11-9
80	21542415	Smith TJ 2011	A pilot trial of decision aids to give truthful prognostic and treatment information to chemotherapy patients with advanced cancer.	The journal of supportive oncology	10.1016/j.suponc.2010.12.005
81	21555700	Temel JS 2011	Longitudinal perceptions of prognosis and goals of therapy in patients with metastatic non-small-cell lung cancer: results of a randomized study of early palliative care.	Journal of clinical oncology : official journal of the American Society of Clinical Oncology	10.1200/JCO.2010.32.4459
82	20597705	Gaertner J 2010	Implementing WHO recommendations for palliative care into routine lung cancer therapy: a feasibility project.	Journal of palliative medicine	10.1089/jpm.2009.0399

Table S5b. Axis classification, B/C/E dimension-presence flags, integration depth and framework categories (n = 82)

#	PMID	Author, Year	D	S	T	B	C	E	Depth	Inductively derived framework categories
1	41932293	Aleixo G 2026	A	C	C	—	•	—	two-of-three	Geriatric-assessment-driven oncology; Digital / ePRO symptom monitoring and decision support
2	41512250	Aleixo G 2026	A	C	C	•	•	•	two-of-three	Geriatric-assessment-driven oncology; Digital / ePRO symptom monitoring and decision support
3	41778625	Hao S 2026	A	C	A	•	•	•	one-centrally	Trigger-based palliative-care integration; Multidisciplinary supportive care intervention
4	41880032	Kessler ER 2026	M	C	C	—	•	•	two-of-three	Geriatric-assessment-driven oncology
5	42211526	Li S 2026	M	C	M	—	•	•	all-three	Early integrated palliative care; Advance care planning and goals-of-care communication
6	40499588	Bergerot CD 2025	M	C	M	•	•	•	two-of-three	Geriatric-assessment-driven oncology
7	41109944	Hillen B 2025	A	C	A	•	•	—	one-centrally	Digital / ePRO symptom monitoring and decision support
8	41379446	Huang HL 2025	M	C	C	—	•	•	all-three	Prognosis-informed shared decision-making; Early integrated palliative care
9	40122129	Kazmi F 2025	C	A	C	•	•	—	two-of-three	Other / uncategorized
10	39566832	Norman KE 2025	M	C	A	—	•	•	two-of-three	Trigger-based palliative-care integration; Early integrated palliative care
11	39982729	Parikh RB 2025	C	C	A	—	•	—	two-of-three	Trigger-based palliative-care integration
12	40812801	Richard HA 2025	C	C	A	•	•	•	two-of-three	Early integrated palliative care; Prognosis-informed shared decision-making
13	38521641	Alibhai SMH 2024	A	C	M	—	•	—	two-of-three	Geriatric-assessment-driven oncology; Multidisciplinary supportive care intervention
14	41915775	Basch E 2024	A	C	M	—	•	—	two-of-three	Digital / ePRO symptom monitoring and decision support
15	38326125	Daviu Cobian C 2024	A	C	C	•	•	—	two-of-three	Geriatric-assessment-driven oncology; Multidisciplinary supportive care intervention
16	39340169	Ermers DJ 2024	M	A	C	—	—	•	two-of-three	Prognosis-informed shared decision-making
17	39259563	Greer JA 2024	A	C	A	—	—	—	one-centrally	Early integrated palliative care; Digital / ePRO symptom monitoring and decision support
18	38193250	Hjermstad MJ 2024	M	C	C	•	•	•	all-three	Early integrated palliative care; Prognosis-informed shared decision-making
19	39115845	Kang E 2024	A	C	M	—	•	•	one-centrally	Early integrated palliative care; Advance care planning and goals-of-care communication
20	38726807	Klastersky J 2024	C	C	C	•	•	•	all-three	Prognostic staging / risk-stratification framework; Early integrated palliative care
21	39690895	Laryionava K 2024	C	M	C	•	•	•	all-three	Prognosis-informed shared decision-making
22	36822846	Marret L 2024	A	C	M	—	•	•	two-of-three	Early integrated palliative care

#	PMID	Author, Year	D	S	T	B	C	E	Depth	Inductively derived framework categories
23	39556485	Matsumoto Y 2024	A	C	A	—	•	—	one-centrally	Trigger-based palliative-care integration; Multidisciplinary supportive care intervention
24	32826262	Nelson A 2024	M	M	C	•	•	•	all-three	Prognosis-informed shared decision-making
25	39088825	Phillips I 2024	C	C	C	•	•	•	all-three	Prognostic staging / risk-stratification framework
26	38631891	Phillips I 2024	A	C	A	•	•	—	one-centrally	Multidisciplinary supportive care intervention
27	34750145	Potenza L 2024	C	C	A	—	•	•	two-of-three	Early integrated palliative care; Advance care planning and goals-of-care communication
28	38824442	Temel JS 2024	A	C	A	—	•	—	one-centrally	Early integrated palliative care; Trigger-based palliative-care integration
29	36574052	Chvetzoff G 2023	A	C	C	—	•	•	two-of-three	Early integrated palliative care; Prognosis-informed shared decision-making
30	36356279	Culakova E 2023	A	C	M	•	•	—	two-of-three	Geriatric-assessment-driven oncology
31	37231417	Deis N 2023	A	M	C	—	•	•	two-of-three	Prognosis-informed shared decision-making
32	37200607	Nishijima TF 2023	A	C	C	•	•	—	two-of-three	Geriatric-assessment-driven oncology
33	37154941	Poisson C 2023	M	C	C	•	•	•	all-three	Advance care planning and goals-of-care communication; Prognosis-informed shared decision-making
34	34922770	Davidson BA 2022	M	A	C	•	—	•	two-of-three	Advance care planning and goals-of-care communication
35	35079813	Matsumoto Y 2022	A	C	A	—	•	—	one-centrally	Trigger-based palliative-care integration; Multidisciplinary supportive care intervention
36	36167377	Matsuoka A 2022	A	C	M	•	•	•	one-centrally	Geriatric-assessment-driven oncology
37	35597176	Ribi K 2022	M	C	C	•	•	•	all-three	Prognosis-informed shared decision-making
38	36071303	van der Padt-Pruijsten A 2022	M	C	A	•	•	•	two-of-three	Trigger-based palliative-care integration; Advance care planning and goals-of-care communication
39	32202060	Conduit C 2021	A	C	M	—	•	•	one-centrally	Advance care planning and goals-of-care communication; Trigger-based palliative-care integration
40	33983395	Hall PS 2021	A	M	C	•	•	•	two-of-three	Prognosis-informed shared decision-making
41	33432524	Hui D 2021	C	M	C	•	•	•	all-three	Prognosis-informed shared decision-making
42	34591080	Li D 2021	A	C	A	•	•	•	one-centrally	Geriatric-assessment-driven oncology
43	33417481	Lu Z 2021	A	C	A	•	•	—	one-centrally	Multidisciplinary supportive care intervention
44	34741815	Mohile SG 2021	A	C	C	•	•	—	two-of-three	Geriatric-assessment-driven oncology
45	32019540	Huang HL 2020	A	M	C	—	•	•	two-of-three	Advance care planning and goals-of-care communication; Trigger-based palliative-care integration
46	32937942	Lynch FA 2020	A	C	A	—	•	•	one-centrally	Multidisciplinary supportive care intervention
47	31697365	Mohile SG 2020	A	C	A	•	•	—	one-centrally	Geriatric-assessment-driven oncology
48	32031887	Temel JS 2020	M	C	M	—	•	•	all-three	Early integrated palliative care; Prognosis-informed shared decision-making
49	31867978	Verhoef MJ 2020	M	C	M	•	•	•	all-three	Early integrated palliative care
50	30157701	Corsi DC 2019	C	C	C	•	•	•	all-three	Early integrated palliative care; Trigger-based palliative-care integration
51	30913904	Park EJ 2019	A	C	A	—	—	•	one-centrally	Advance care planning and goals-of-care communication
52	30168410	Tapp D 2019	A	M	C	•	•	•	two-of-three	Prognosis-informed shared decision-making
53	31783851	van Lent LGG 2019	A	M	C	—	—	•	two-of-three	Prognosis-informed shared decision-making
54	30677754	Villalobos M 2019	C	C	C	•	•	•	all-three	Prognosis-informed shared decision-making; Early integrated palliative care
55	28734786	Engelhardt EG 2018	C	A	C	•	•	—	two-of-three	Prognosis-informed shared decision-making
56	29310605	Henselmans I 2018	M	A	C	•	—	•	two-of-three	Prognosis-informed shared decision-making
57	30344075	Kaasa S 2018	C	C	C	•	•	•	all-three	Early integrated palliative care
58	29907553	Laryionava K 2018	A	A	C	—	•	•	one-centrally	Advance care planning and goals-of-care communication
59	30652585	Wright AA 2018	A	C	A	•	•	—	one-centrally	Digital / ePRO symptom monitoring and decision support
60	28306372	Adelson K 2017	M	C	M	•	•	•	all-three	Trigger-based palliative-care integration; Early integrated palliative care
61	28562197	Einstein DJ 2017	A	C	A	•	•	—	one-centrally	Trigger-based palliative-care integration; Early integrated palliative care
62	28761725	Martoni AA 2017	A	C	C	—	•	•	two-of-three	Early integrated palliative care; Advance care planning and goals-of-care communication
63	28859646	Oostendorp LJM 2017	M	A	C	•	•	•	two-of-three	Prognosis-informed shared decision-making
64	28561696	Smith CB 2017	M	C	M	•	•	•	all-three	Early integrated palliative care; Prognosis-informed shared decision-making
65	28029308	Temel JS 2017	M	C	M	—	•	•	all-three	Early integrated palliative care; Prognosis-informed shared decision-making
66	28470369	Vayne-Bossert P	A	C	A	—	•	•	one-centrally	Early integrated palliative care

#	PMID	Author, Year	D	S	T	B	C	E	Depth	Inductively derived framework categories
		2017								
67	26884557	Corre R 2016	A	A	C	●	●	—	one-centrally	Geriatric-assessment-driven oncology
68	26768772	Grudzen CR 2016	A	C	A	—	●	●	one-centrally	Trigger-based palliative-care integration; Advance care planning and goals-of-care communication
69	27821313	Maltoni M 2016	M	C	M	●	●	●	all-three	Early integrated palliative care; Trigger-based palliative-care integration
70	26646758	Strasser F 2016	A	C	A	●	●	—	one-centrally	Digital / ePRO symptom monitoring and decision support
71	26559912	Clarke G 2015	M	A	C	●	●	●	two-of-three	Prognosis-informed shared decision-making
72	25872950	do Carmo TM 2015	A	C	A	—	●	—	one-centrally	Early integrated palliative care; Multidisciplinary supportive care intervention
73	25173586	Rugno FC 2014	A	C	A	—	—	—	one-centrally	Early integrated palliative care; Advance care planning and goals-of-care communication
74	24654196	Colombet I 2012	M	C	C	●	●	●	all-three	Prognosis-informed shared decision-making
75	22831160	Vigano A 2012	A	C	A	●	●	●	one-centrally	Prognostic staging / risk-stratification framework
76	21779232	Gaertner J 2011	M	C	A	●	●	●	two-of-three	Early integrated palliative care; Trigger-based palliative-care integration
77	21483008	Leighl NB 2011	C	A	C	●	●	●	two-of-three	Prognosis-informed shared decision-making
78	22158486	Magarotto R 2011	A	C	A	—	●	●	one-centrally	Early integrated palliative care; Advance care planning and goals-of-care communication
79	21320319	Oostendorp LJ 2011	C	A	C	●	●	●	two-of-three	Prognosis-informed shared decision-making
80	21542415	Smith TJ 2011	C	M	C	●	●	●	all-three	Prognosis-informed shared decision-making
81	21555700	Temel JS 2011	C	C	M	—	●	●	all-three	Early integrated palliative care; Prognosis-informed shared decision-making
82	20597705	Gaertner J 2010	A	C	A	—	●	—	one-centrally	Early integrated palliative care; Trigger-based palliative-care integration
