# Supplementary material for: Mapping Integrative Decision-Making Frameworks in Advanced Cancer Care: A Scoping Review of Diagnostic, Supportive-Care, and Therapeutic Integration
Source: Cancers (Basel). 2026 Oct 8;18(19):3245. doi: 10.3390/cancers18193245 (PMC13664350; doi:10.3390/cancers18193245)
Supplement: Supplementary file 1 [file cancers-18-03245-s001.zip › Table_S6_Tumour_type_representation_v4_n82.pdf]

Supplementary Table S6

Tumour-type representation of the included studies (n = 82)

Tumour type was charted as a single free-text description of each study population, so two measures can be taken from it and they are not interchangeable. Table S6a assigns one predominant type per record and sums to 82; Table S6b counts the records in which a type forms part of the population, so a mixed-tumour cohort contributes to more than one row and the percentages do not sum to 100%.

Derivation. Neither panel is a pre-existing per-record classification; both were derived from the charted tumour field under the rules stated here, and both are published per record in Supplementary File S8 — the charted text, the derived predominant type and the derived list of types represented anywhere — so every assignment can be checked. For Table S6a a record takes a named type when the charted text gives it an explicit majority adjacent to its name, names it as the only type, or leads with it; otherwise the record is a mixed-tumour population. For Table S6b a type named only to record its exclusion, or appearing only in an example table of a consensus document, is not counted, which affects three records.

Counts in Table S6a are mutually exclusive and sum to 82. Counts in Table S6b are not mutually exclusive, because a study population may include more than one tumour type; therefore the percentages do not sum to 100%. Lung is the most frequently represented single predominant tumour type, but mixed-tumour populations are the largest group overall. A blank derived-list field in Supplementary File S8 indicates that no named tumour type was counted for that record under the Table S6b derivation rules; it is not a missing value.

**Tumour type**	**n**	**% of 82**
**Table S6a. Predominant tumour type (mutually exclusive; sums to 82)**		
Mixed tumour-type population	35	42.7
Lung	17	20.7
Breast	5	6.1
Haematological	4	4.9
Gastro-oesophageal	3	3.7
Hepatopancreatobiliary	3	3.7
Gynaecological	3	3.7
Sarcoma	2	2.4
Prostate	2	2.4
Gastrointestinal, site unspecified	2	2.4
Melanoma	2	2.4
Colorectal	2	2.4
Genitourinary	1	1.2
Head and neck	1	1.2
**Total**	**82**	**100.0**
**Table S6b. Tumour types represented anywhere in the study population (not mutually exclusive)**		
Lung	39	47.6
Breast	27	32.9
Gastrointestinal, site unspecified	24	29.3
Gynaecological	21	25.6
Genitourinary	17	20.7
Colorectal	17	20.7
Haematological	11	13.4
Gastro-oesophageal	11	13.4
Hepatopancreatobiliary	11	13.4
Prostate	10	12.2
Head and neck	6	7.3
Melanoma	6	7.3
Sarcoma	4	4.9
Brain/CNS	2	2.4
